# Functional Classification of Zn^2+^-Dependent Histone Deacetylase Homologs and Variants Using Chemical Profiling

**DOI:** 10.3390/ijms27219595

**Published:** 2026-10-27

**Authors:** Aleksandra Kopranovic, Hannah Münster, Aaron Rosowski, Louis Groll, Florian Almstätter, Eva Attenhauser, Franz-Josef Meyer-Almes

**Affiliations:** 1 Department of Chemical Engineering and Biotechnology, Darmstadt University of Applied Sciences, Haardtring 100, 64295 Darmstadt, Germany; 2 European University of Technology, European Union, 64290 Darmstadt, Germany

**Keywords:** HDAC inhibitors, inhibitor binding profiles, chemical profiling of enzymes, HDAC classification

## Abstract

Zinc-dependent histone deacetylases (HDACs) are found in all species, ranging from bacteria to eukaryotes. They catalyze the removal of acetyl groups or various organic acid residues, including fatty acids, from proteins or polyamines. These enzymes are located in various cellular compartments, and their function is modulated by their ability to form different protein complexes. HDACs are prominent targets in the study of human diseases and infections, but they have also recently been considered as targets for fungicides. The active sites of HDACs are highly conserved, both structurally and sequentially. Therefore, the development of isoenzyme-selective inhibitors poses an exceptionally great challenge. In this study, a chemical profiling approach was used to classify human HDAC isoenzymes, bacterial homologs, and mutant variants by function and to identify specific ligand-binding patterns. While the recognition profiles generally reflected established HDAC classes, HDAC8 exhibited significant differences from HDAC1–3. In contrast, HDAC6 demonstrated substantial similarity with HDAC1–3 despite their low sequence identity, distinguishing itself from the sequentially closely related HDAC10. These findings demonstrate that sequence similarity alone is not a reliable predictor of ligand recognition and underscore the importance of local active-site architecture and physicochemical properties. Zebrafish HDAC10 closely resembles human HDAC10, supporting its use as a functional surrogate, while bacterial homologs display distinct profiles that suggest opportunities for species-selective inhibitor development. A single active-site substitution in class IIa HDAC4 (H976Y) shifted its recognition profile from HDAC class IIa toward class I. Engineered HDAC11 variants showed substantially improved recombinant expression and comparable stability while retaining wild-type-like ligand recognition. This supports their use in screening, biophysical, and structural studies. Together, these results establish ligand recognition profiling as a practical approach for functionally classifying HDACs and guiding the development of selective inhibitors.

## 1. Introduction

Zn^2+^-dependent histone deacetylases (HDACs) and acetylpolyamine amidohydrolases (APAHs) belong to the deacetylase superfamily of enzymes (EC 3.5.1.98). The structures of Zn^2+^-dependent HDACs exhibit a highly conserved α/β-arginase/deacetylase fold. Based on sequence homology, HDACs have been classified into four classes. In humans, class I consists of HDACs 1, 2, 3, and 8, although HDAC8 differs significantly from the other members of class I in terms of its exceptionally high structural flexibility around the active site and its distinct substrate specificity. Furthermore, unlike the other class I members, HDAC8 does not occur in multi-enzyme complexes. Class IIa includes HDACs 4, 5, 7, and 9, which, in addition to the catalytic deacetylase domain, contain a large N-terminal domain that binds additional proteins, allowing them to form large multiprotein complexes. Class IIa HDACs can shuttle between the nucleus and the cytosol. Class IIb consists of HDACs 6 and 10. These enzymes are characterized by two deacetylase domains, although the second domain in HDAC10 is truncated and inactive. Class III contains so-called sirtuins, which are NAD+-dependent and are excluded from this study. Class IV consists of only one enzyme, HDAC11, which differs significantly from the other HDACs in terms of sequence and substrate specificity. HDACs are found not only in vertebrates and simple eukaryotic species but also in all biological kingdoms, down to single-celled bacteria. This is somewhat surprising, since bacteria do not have histones. However, DNA binding by polyamines appears to be comparable to DNA binding by histones in higher organisms, and the acetylation status of polyamines could also result in different accessibility of transcription factors to bacterial DNA. Therefore, Leipe et al. hypothesized that eukaryotic HDACs originated from a prokaryotic acetylpolyamine amidohydrolase (APAH) to achieve a gene regulatory effect [1]. The close functional relationship between HDACs and APAHs is evident in the capacity of APAHs to deacetylate common HDAC substrates and even eukaryotic histones [2]. HDACs and APAHs with catalytic zinc ions are structurally closely related and are both inhibited by canonical HDACs, such as SAHA [3,4]. Beyond histones, numerous additional substrates are now known, where HDACs remove acetyl groups from lysines or other nuclear or cytosolic proteins. Consequently, HDACs are now often referred to as K-deacetylases or KDACs [5]. It was later recognized that some KDACs are capable of cleaving larger acyl groups from lysines, such as formyl, butyl, propionyl, succinyl, malonyl, myristoyl, glutaryl, crotonyl, as well as long-chain fatty acid modifications [6,7,8]. For example, human HDAC8 can cleave not only acetyl groups but also acyl groups with 2–16 carbons from lysine 9 of histone H3 peptide. Moreover, the catalytic efficiencies of HDAC8 in terms of k_cat_/K_M_ on octanoylated, dodecanoylated or myristoylated lysine are even higher than that of acetylated lysine [8]. Similarly, the catalytic efficiency of HDAC11 on H3 acyl peptides with myristoyl modification at a lysine residue is approximately four orders of magnitude higher than on the corresponding acetylated lysine [7]. In general, HDACs catalyze both deacylation and acylation of lysine-containing substrates. An interesting recent example of this reversed reaction is HDAC6, which can also act as a lactyltransferase to catalyze lactylation of α-tubulin, thereby enhancing microtubule dynamics in neurons [9]. In contrast to most other Zn^2+^-dependent HDACs, class IIa HDACs exhibit a thousandfold lower deacetylation efficiency compared to class I HDACs, which is why they are physiologically attributed primarily a role as scaffolding proteins for recruiting other effector proteins into multiprotein complexes. This is primarily due to the replacement of a catalytically important tyrosine by histidine. Despite its very low catalytic efficiency, HDAC4 has been reported to control muscle homeostasis by deacetylating three physiological substrate proteins: myosin heavy chain, peroxisome proliferator-activated receptor gamma coactivator 1-alpha (PGC-1α), and heat shock cognate 71 kDa protein (Hsc70) [10]. The preferred substrates for human HDAC10, a class IIb enzyme, are acetylated polyamines [11], whereas the only other second-class IIb HDAC, HDAC6, has been shown to possess a distinct substrate recognition scheme and has been observed to preferentially deacetylate acetylated cellular proteins, including α-tubulin [12]. Some bacterial HDAC homologs, for example, PA0321 and PA1409, exhibit amidohydrolase functionality and recognize acetylated polyamines, such as N^1^-acetylspermine, N^1^-acetylspermidine, N-acetylcadaverine and N-acetylputrescine [13]. In addition, bacterial HDACs have been shown to partially deacetylate artificial (trifluoro)acetylated lysine substrates and eukaryotic histones in vitro, despite the fact that these histones are not their native substrates. Other bacterial homologs, such as the HDAC-like amidohydrolase (HDAH) found in *Bordetella* or *Alcaligenes* strain FB188, also accept eukaryotic acetylated histones as substrates [2]. A more detailed investigation of the substrate specificity of HDAH using a library of tripeptide fluorogenic substrates containing an acetylated lysine revealed remarkable specificity for basic amino acids at the P2 position and serines—or rather, small aliphatic and hydrophobic amino acids—at the P1 position [14]. This peptidic substrate library also revealed that HDAC1, HDAC3, and HDAC6 have very similar substrate specificities, while HDAC8 and HDAH exhibit distinct substrate profiles. In a recent study, Lammers et al. provided a comprehensive overview on the diversity of Zn^2+^-dependent HDACs in bacteria and classified them into five clusters [15]. An extensive analysis of functional assays and crystal structures of representative enzymes suggests that bacterial HDACs have evolved into deacylases with different substrate specificities and lysine-acyl-chain preferences. Interestingly, almost all representative cluster members can be inhibited by canonical mammalian HDACis, such as SAHA and TSA. Based on this, the authors hypothesized that new antibacterial therapeutics could be developed using known HDACis as a starting point. There are now five approved HDACis (vorinostat [16], romidepsin [17], belinostat [18], panobinostat [19] and tucidinostat [20]) for treating various types of blood cancer. The most recently approved drug, givinostat, is intended for the treatment of Duchenne muscular dystrophy [21]. Many other HDACis are currently in active clinical trials or seeking study participants [22]. Flufenoxadiazam, a fungicidal HDACi that is effective in controlling Asian soybean rust, demonstrates that HDACs can also be targeted in other organisms [23].

This study aims to establish a ligand recognition-based approach to HDAC classification, with three main objectives: homolog classification, variant characterization, and HDAC11 engineering. First, we seek to classify HDAC homologs according to their chemical inhibition profiles rather than their sequences, potentially facilitating the adaptation of established HDAC inhibitors to target previously uncharacterized bacterial and fungal homologs. Second, we aim to characterize how individual mutations affect ligand recognition, providing functional insight into the binding pocket surrounding the active site and assessing whether mutations can shift inhibition profiles toward those of other HDAC classes. Third, we aim to engineer HDAC11 variants with improved expression and stability comparable to that of the wild-type enzyme, using chemical profiling to assess whether these variants retain the wild-type ligand recognition profile.

## 2. Results and Discussion

Based on the canonical classification of HDACs according to sequence similarity, we extensively investigate the effects of a panel of structurally diverse compounds on different HDACs from various species, as well as on mutation variants, to achieve a functional clustering of HDACs with similar protein–ligand recognition patterns.

### 2.1. Selection of Chemical Structures for Chemical Profiling

**Figure 1:**
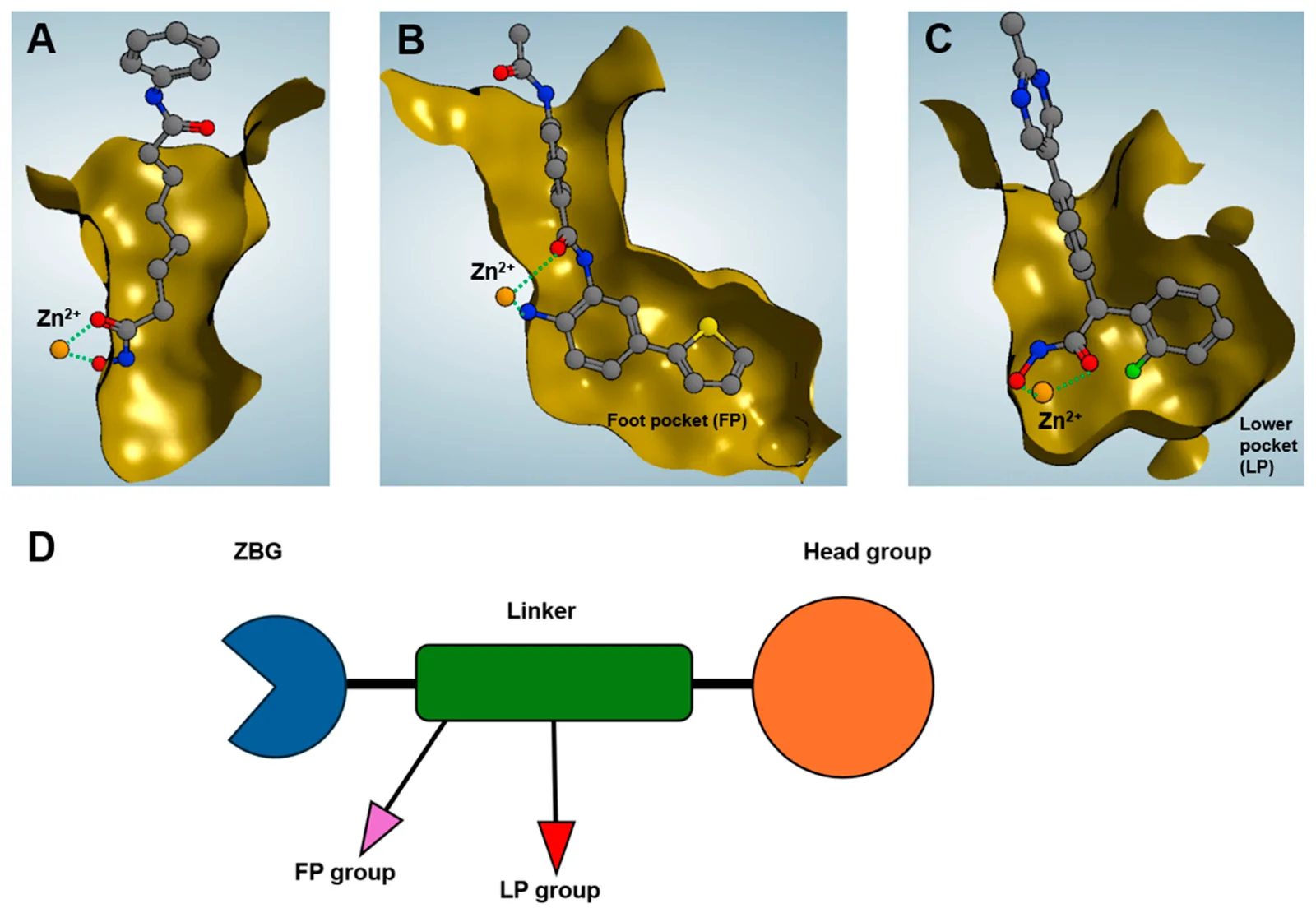
Pharmacophore concept for HDACis. (**A**) SAHA bound to HDAC8 (PDB-ID: 1T69) according to the classical pharmacophore model consisting only of the ZBG, linker and head group. (**B**) A p-thienyl-anilinobenzamide analog bound to HDAC2 (PDB-ID: 4LY1). The thienyl group occupies a specific foot pocket (FP) located at the bottom of the active site. (**C**) A diphenylmethyl-hydroxamic acid analog bound to HDAC4 (PDB-ID 6FYZ). This branched HDACi occupies a lower pocket (LP) which is characteristic for class IIa HDACs. (**D**) Slightly modified, extended pharmacophore model based on Sippl et al. [24]. This model considers the crystal structures of HDAC-ligand complexes, where the inhibitors fill characteristic subpockets, such as the foot pocket (FP) in HDAC2 or the lower pocket (LP) in HDAC4, thereby creating isoenzyme selectivity. The binding pocket is shown as clipped brown surface and zinc bonds are marked with dotted green lines.

**Figure 2:**
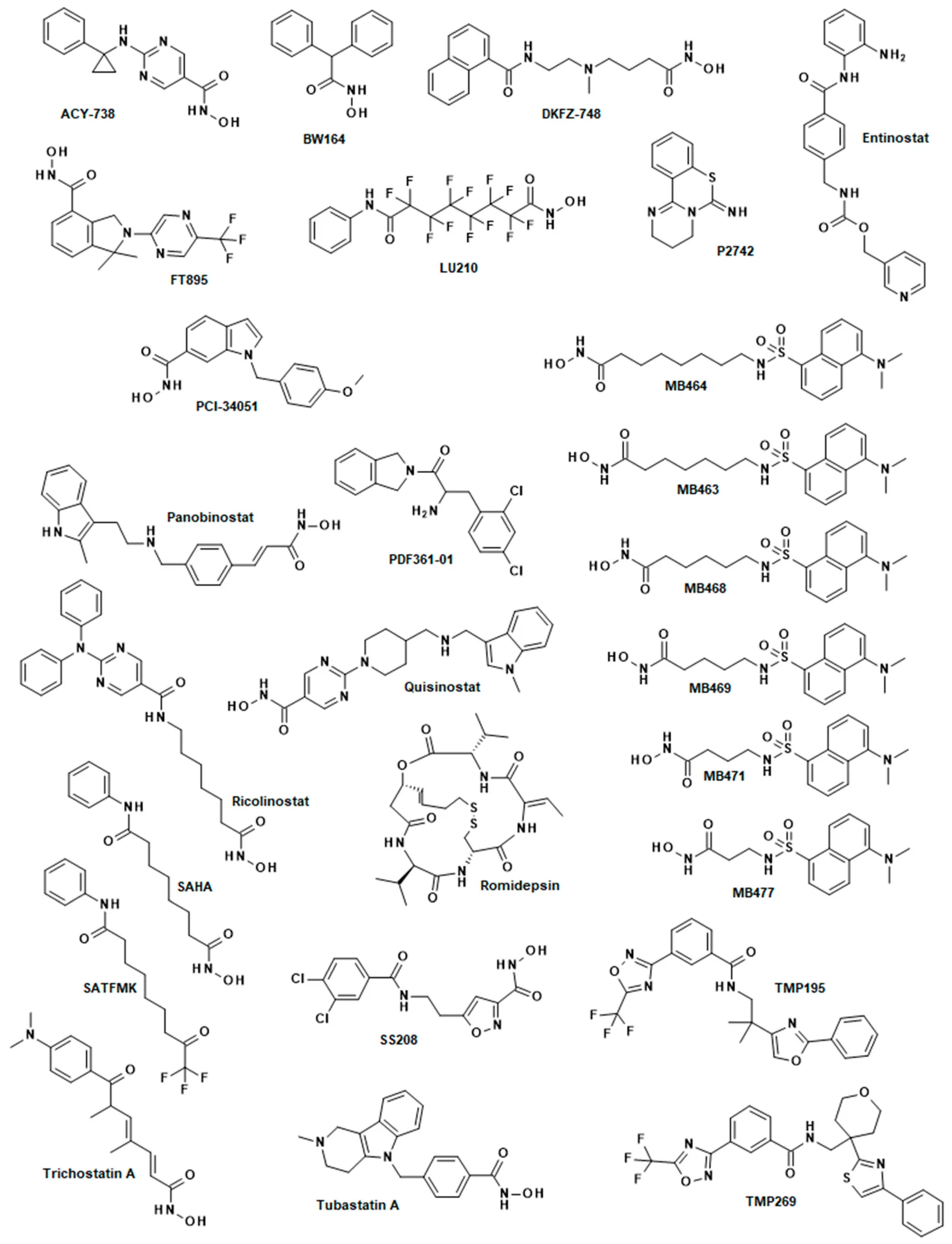
Chemical structures of the set of HDACi profile compounds applied in this study.

The classical pharmacophore concept of an HDAC inhibitor simply consists of a zinc-binding group (ZBG), a linker, and a head group (Figure 1). The extended pharmacophore model, according to Sippl et al., also considers subpocket differences among different HDAC classes, for example, the foot pocket in HDAC2 (PDB-ID: 4LY1) or the lower selectivity pocket in class IIa HDACs (PDB-ID: 6FYZ) (Figure 1B–D) [24]. We aimed to select structurally diverse HDACis with distinct isoenzyme selectivities (Figure 2). The compounds include those with varying linker chain lengths and bulkiness, such as MB463, MB464, MB468, MB469, MB471, and MB477. They also include compounds that differ only in their ZBGs, such as SAHA and SATFMK; those that differ only in their linkers, such as SAHA and LU210; and those with different combinations of ZBGs, linkers, and head groups. Variations in the ZBG and linker can occupy specific subpockets to create selectivity, while head groups enable specific interactions with the less conserved protein surface at the rim of the active site. The analysis of compound similarity using the Tanimoto coefficient and SkelSpheres descriptors, as calculated by DataWarrior software v06.06.04, revealed only one large cluster of related structures consisting of the MB series containing the dansyl head group with Tanimoto similarities ≥ 0.92, and two small clusters, each consisting of only two members (Figure S1). One of these clusters contains TMP195 and TMP269 (Tanimoto coefficient = 0.849), whereas the second, smaller cluster contains SAHA and SATFMK (Tanimoto coefficient = 0.845). All other substances are dissimilar to one another, exhibiting Tanimoto similarities of less than 0.8, thus demonstrating the significant structural diversity of these profile compounds. The deliberate selection of these profile compounds allowed for the systematic exploration of the electronic and steric conditions within the active-site binding pockets of the HDACs of interest.

### 2.2. Chemical Profiling of HDAC Enzymes

**Figure 3:**
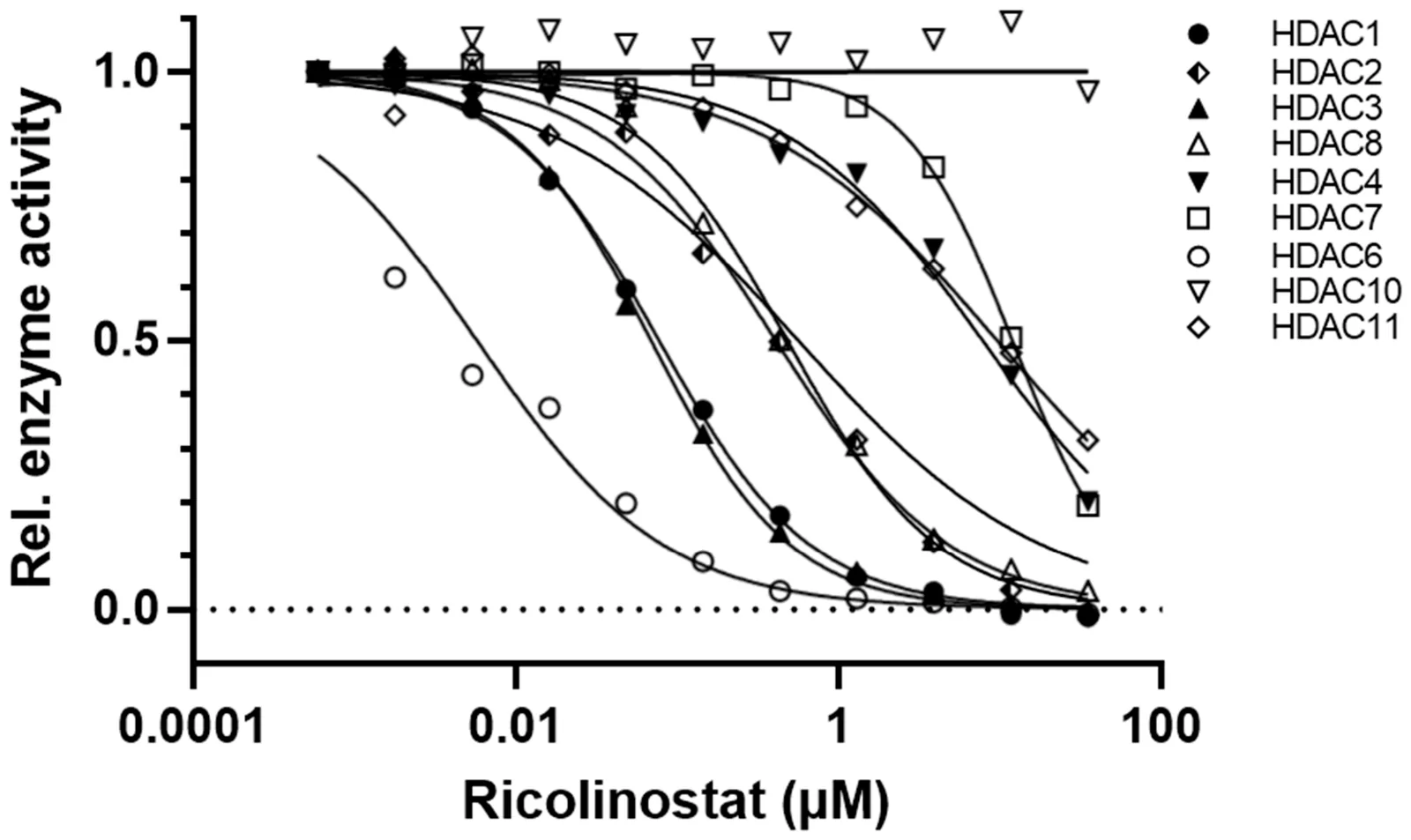
Representative dose–response curves for ricolinostat against indicated human HDACs.

For chemical profiling, we determined the effects of the selected 26 structurally diverse HDACis on various HDACs (Figure 2) with different isoenzyme selectivity using a standardized enzyme activity assay. Given the high sequence identities (75–81%) and the highly conserved structures of the catalytic domains of all human class II HDACs, as evidenced by crystal structures of apo-proteins and ligand–enzyme complexes (for example, PDB-IDs 2VQM, 2VQJ, 6FYZ, 3C0Y, 3C0Z, 3ZNR), only three of the four class IIa HDACs (HDACs 4, 5, and 7) were examined in this study, with HDAC9 being omitted. High-resolution dose–response curves were measured for each compound at 10 different concentrations, and IC_50_ values were determined via nonlinear regression (Figure 3 and Figure S2). A total of 548 IC_50_ values were determined to obtain highly comparable activity data under identical conditions. In the interest of ensuring the robustness of the data analysis, the negative decadic logarithm of the IC_50_ values was employed. It is noteworthy that a considerable range of IC_50_ values has been observed in the literature for a given compound when tested against specific HDAC enzymes. For instance, the IC_50_ values of SAHA against HDAC1 vary about 300-fold, according to the Binding DB database [25]. The substantial inter-laboratory variations can be primarily attributed to the varying assay conditions, including enzyme concentration, substrate structure and concentration, buffer composition, and pH, which represent the most significant influencing factors. Therefore, it is imperative to acknowledge that the assay conditions were kept constant to ensure a valid comparison between the various compounds. Table 1 shows the negative decadic logarithms of the IC_50_ values, or pIC_50_, of all profile compounds against human wild-type HDACs. The whole set of IC_50_ values, including citation of references and determined 95% confidence intervals, is summarized in Table S1.

The selected compounds exhibit distinct inhibition profiles against the panel of human HDACs, ranging from isoenzyme-selective to class-specific to multitarget-selective (Table 1, Figure 3 and Figure S2). For example, the inhibitors PCI-34051 (HDAC8), ACY-738, SS208, ricolinostat (HDAC6), and FT895 (HDAC11) are significantly monoselective; i.e., they preferentially inhibit only one HDAC isozyme each with a pIC_50_ value > 7.5. Some profile compounds exhibit class specificity, such as entinostat (class I), TMP195 and TMP269 (class IIa). And other compounds exhibit cross-class, multitarget selectivity, such as trichostatin A (class I, but not HDAC8, and HDAC6, but not HDAC10) or SATFMK (class I, class IIa, and class IIb, but not HDAC11). The activity profiles of all inhibitors across all human HDACs were correlated, and the resulting Pearson coefficients, r, provided a measure of similar isoenzyme recognition and, consequently, selectivity. Ranking the compounds by their Pearson’s coefficient to SAHA revealed a large group of compounds with similar active-site recognition across all the tested human HDAC isozymes (Figure S3). These compounds include SAHA, trichostatin A, compounds from the MB series with linker-chain length longer than two methylene groups, panobinostat, ricolinostat, and SS208. These HDACis conform to the classical pharmacophore model and contain an alkyl- or aryl-linker with at least four carbon atoms and show preferences for class I HDACs and HDAC6. Shorter linkers, such as those found in MB471 and MB477, make it difficult for the ZBG to bind to the catalytic zinc ion at the bottom of the narrow binding channels of class I HDACs and HDAC6 due to steric hindrance. The more rigid linker in trichostatin A still enables the inhibition of class I HDACs and HDAC6, but it also targets class IIa HDACs. A different ZBG, such as trifluoroketone instead of hydroxamic acid, significantly alters the inhibition profile, as demonstrated by SATFMK and SAHA (Pearson’s correlation coefficient r = 0.02). SATFMK strongly inhibits HDACs of all classes, including HDAC10, but not class IV HDAC11. ACY-738 shares a similar HDAC inhibition profile to the other class I/HDAC6-specific HDACis, but it is clearly more selective for HDAC6. ACY-738 is also somewhat correlated with the HDAC6-selective inhibitor tubastatin A. This inhibitor’s high selectivity is attributed to its para-substituted benzene linker with a bulky, branched head group. As expected, the related trifluoromethyloxadiazoles TMP195 and TMP269 show highly correlated HDAC inhibition profiles with strong preference for class IIa HDACs (r = 0.884). Interestingly, the inhibition profiles of the TMP compounds share some similarity with BW164 (r = 0.661 and r = 0.349, respectively), although the chemical structures and ZBGs are clearly distinct. However, an examination of the crystal structures of TMP269 and an analog of BW164 in complex with class IIa HDACs reveals that both compounds occupy the lower-selectivity pocket of HDAC4. This is achieved through one of the branched phenyl groups of the BW164 analog (PDB-ID 6FYZ) or by folding the head group back onto the linker, as observed in the TMP269-HDAC7 complex (PDB-ID 3ZNR). It has been demonstrated that both inhibitors exhibit optimal overlay in the binding pocket. However, it is imperative to note that the bulkier backfolded structure of TMP269 results in more substantial structural adaptations compared to the complex with the BW164 analog. The inhibition profiles of most compounds are either uncorrelated or negatively correlated and, thus, have strongly dissimilar binding patterns (Figure S3). This wide range of distinct chemical profiles, regarding the inhibition of various HDAC isoenzymes, provides a solid foundation for analyzing similarities in ligand recognition by specific HDAC homologs and subsequently classifying them.

### 2.3. Sequence Similarity Relationships Between Human HDACs

**Figure 4:**
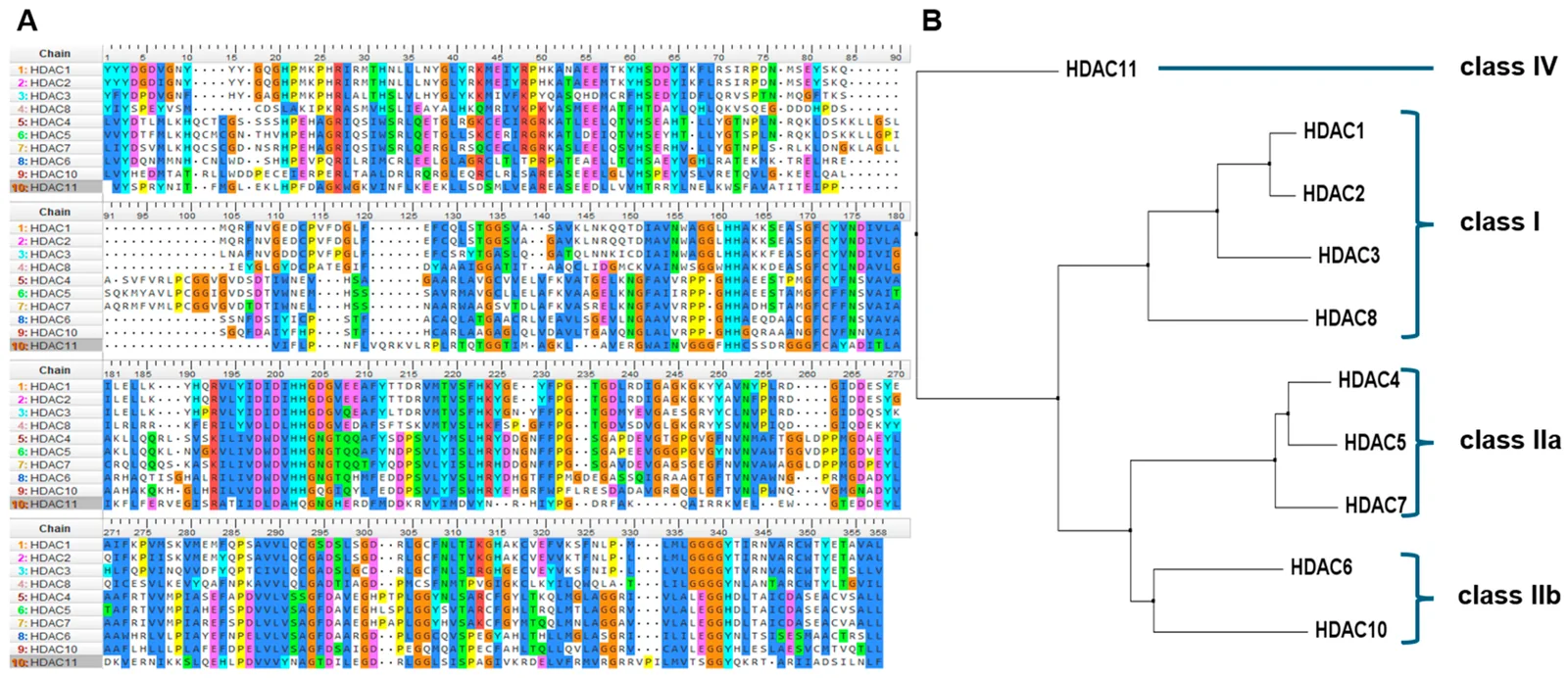
Multiple sequence alignment of the indicated human HDACs (over 358 amino acids) to classify them based on the sequences of their catalytic domains. (**A**) The letters in the Multiple sequence alignment indicate amino acids, which are colored according to similarity (Clustal-X). (**B**) Corresponding guide tree, which can be used to classify HDAC isozymes based on sequence similarities.

Historically, members of the HDAC superfamily have been classified into classes I–IV based on sequence similarities. First, we sought to verify this classification by performing a multiple alignment of the largest possible overlapping region of the catalytic deacetylase domains. The multiple sequence alignment of these regions and the corresponding guide tree revealed sequence similarities that aligned with the canonical classification of HDAC classes (Figure 4). This is also reflected in the heatmap, showing percent sequence identity over 358 amino acids (Figure S4). HDACs 1, 2, and 3 group together in class I in terms of sequence identity, while HDAC8 is a member of class I but is more distantly related to the other class members. Similarly, the sequences of class IIa HDACs 4, 5 and 7 have a high degree of identity. The catalytic domains of HDAC6 (Uniprot Q9UBN7, 483-799) and HDAC10 (Uniprot Q969S8, 5-322) in class IIb are also similar to each other and, to a lesser degree, to class IIa members. In contrast, HDAC11 is unique and the only class IV member, because it shares less than 24% sequence identity with any other HDAC.

### 2.4. Ligand-Binding Profiles of Human HDACs

**Figure 5:**
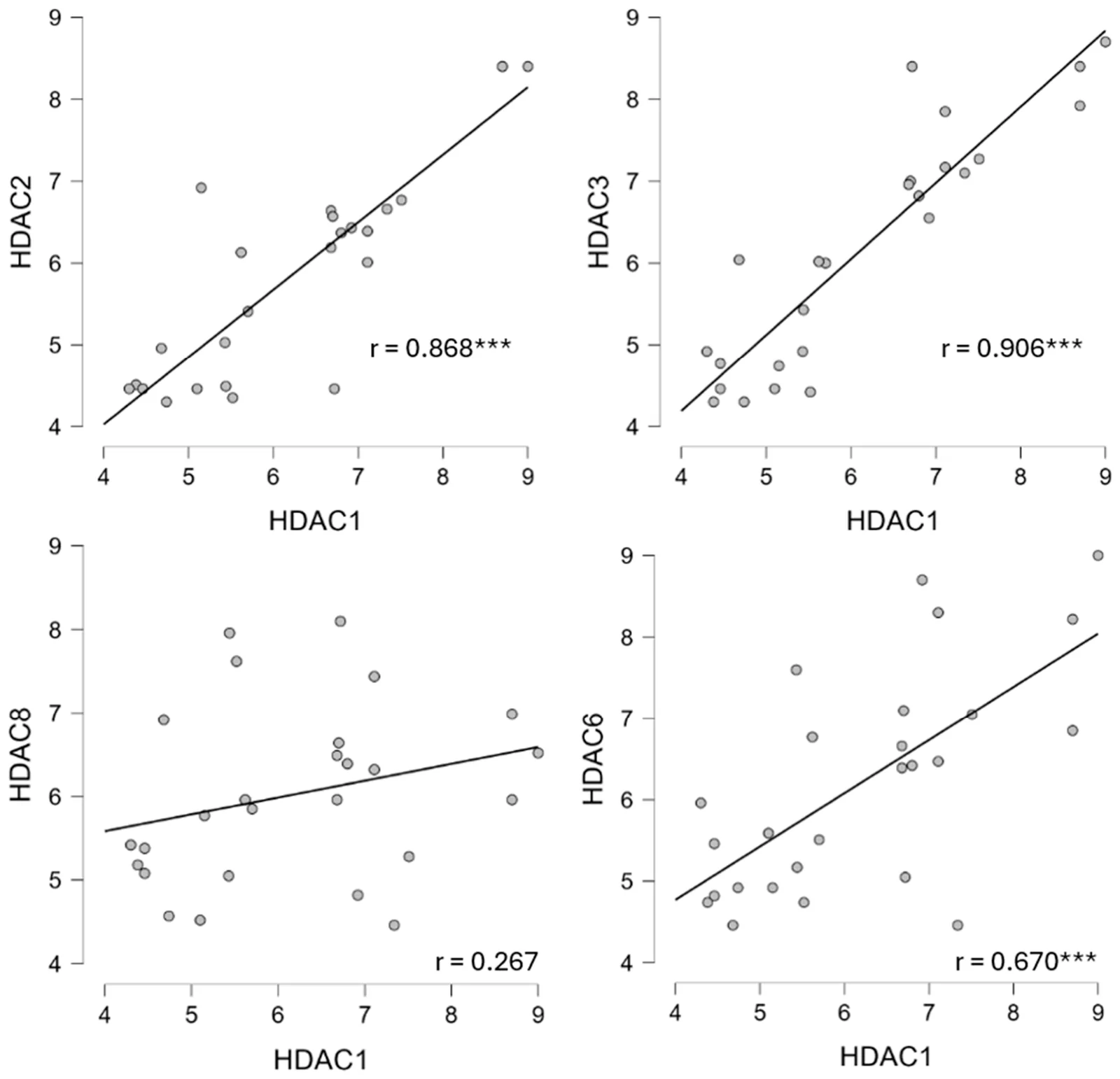
Exemplary plots: Pairs of pIC_50_ values for the profile compounds are plotted against each other for the indicated HDAC isozymes. Each dot represents an HDACi. r is the Pearson’s correlation coefficient. A high correlation between the pIC_50_ values indicates similar ligand binding of the respective HDACs. *** *p* < 0.001. The starred *p*-values are nominal and exploratory. A Benjamini–Hochberg correction was applied to the data. The adjusted *p*-values and significance for each pair are summarized in Table S4.

**Figure 6:**
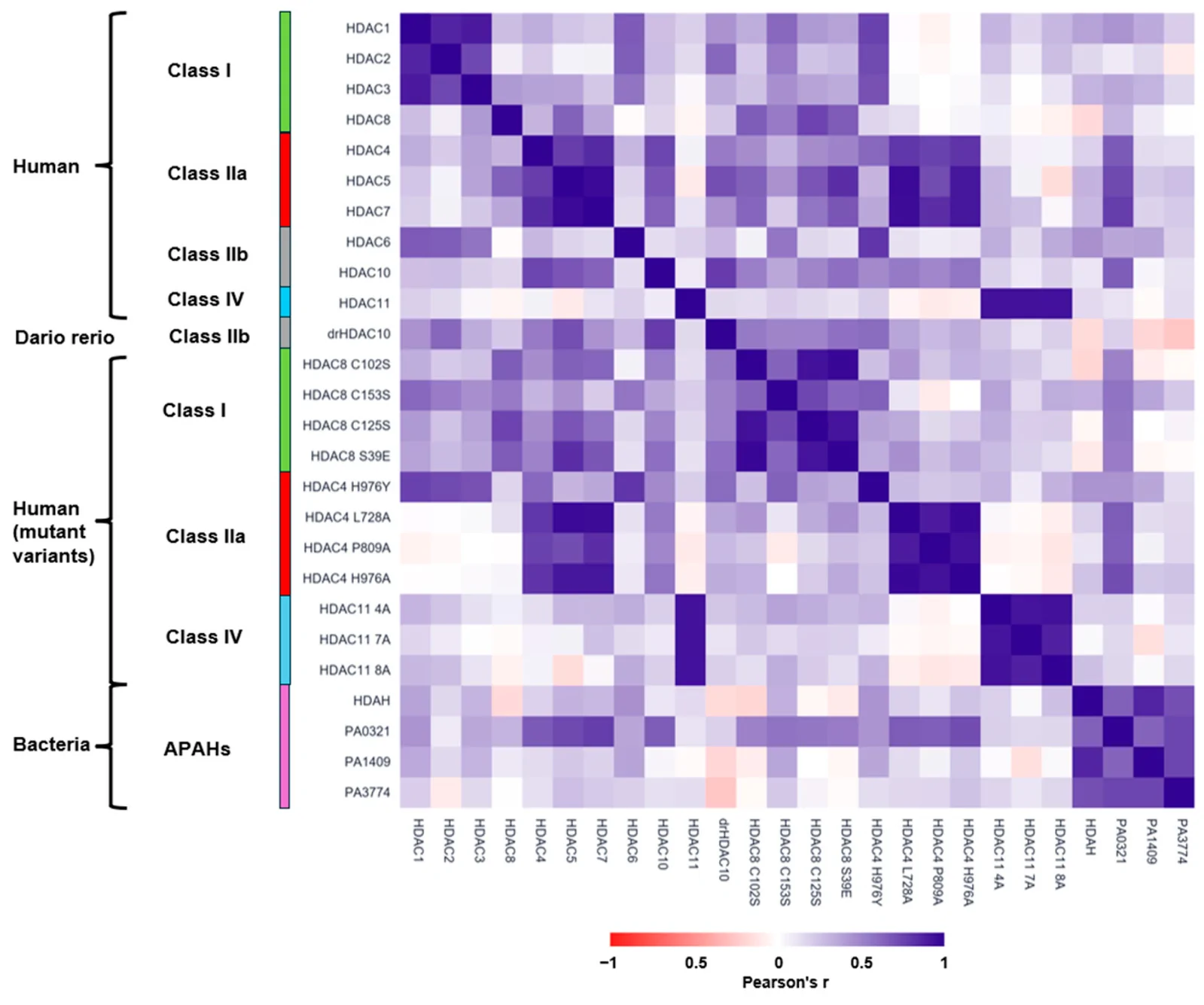
Heatmap of Pearson’s correlation coefficients for chemical profiles of all pairs of HDAC homologs from different species investigated in this study. Blue color means high correlation, i.e., similar inhibition pattern, white indicates no correlation, while red color indicates negative correlation. The exact values of Pearson’s coefficients, starred *p*-values and 95% confidence intervals are provided in Table S2.

Next, we plotted the pIC_50_ values of the profile compounds against each other for every pair of HDAC isozymes. Some exemplary plots are shown in Figure 5. The complete set of correlated pIC_50_ values for all HDAC pairs is presented in the Supplementary Data (Figure S5). We expected enzymes with similar binding pockets to exhibit similar affinities to the different structurally diverse substances. The data demonstrate a clear correlation between the pIC_50_ values of the reference compounds against HDAC1 and HDAC2 and against HDAC1 and HDAC3, respectively (r = 0.868 and 0.906, respectively). Therefore, HDACs 1, 2, and 3 from class I are not only sequence-related but also show high correlation of pIC_50_ values of profile compounds, indicating very similar recognition of structurally diverse ligands and, thus, very similar binding pockets. In contrast, the binding scheme to HDAC8 is clearly different, suggesting that the binding pocket of HDAC8 is distinct from those of HDAC1, HDAC2, and HDAC3. Interestingly, the pIC_50_ values for HDAC1 and HDAC6, which are members of different classes (based on multiple sequence alignments), show a significant correlation (r = 0.670), while there is no significant correlation between HDAC1 and HDAC8 (*p* = 0.187), despite both being members of the same class I HDAC. However, Steiger’s test revealed that the correlations of HDAC1/HDAC6 (r = 0.670) and HDAC1/HDAC8 (r = 0.267) did not differ significantly (Steiger’s z = 1.72, *p* = 0.085). Pearson’s correlation coefficients for the pairwise comparison of pIC_50_ values between all combinations of HDAC enzymes are summarized and provided in Table S2 and displayed in a heatmap (Figure 6).

HDACs 1, 2, and 3 also share significant correlation of ligand recognition with class IIb member HDAC6 (r = 0.670, r = 0.661, and r = 0.571, respectively) but not with HDAC10 (r < 0.277). Interestingly, the similarity in the ligand recognition patterns between class I HDACs and HDAC6 is not reflected in the sequence homology of their entire catalytic domains (sequence identities across 358 amino acids = 22.7–26.6%). However, this finding shows that sequence similarities do not necessarily result in similar ligand recognition patterns. Rather, they depend on the conformational structure and chemical microenvironment of the active-site pocket. This fact is important, for example, for the development of HDAC6-selective compounds with low preference for class I HDACs. Although class IIb HDACs 6 and 10 share about 54% sequence identity, their ligand recognition patterns are completely unrelated, with a Pearson’s coefficient of 0.146. This indicates opportunities for the development of intra-class-selective HDACis. This is also reflected by the fact that HDAC6 and HDAC10 process different substrates. HDAC6 is a lysine deacetylase, while HDAC10 is an acetylpolyamine amidohydrolase. Consequently, one of the best monoselective HDAC10 inhibitors is DKFZ-748, which contains a methylated nitrogen in its alkyl linker chain that mimics acetylpolyamine substrates and thereby creates selectivity [26]. The ligand recognition profiles of class IIa HDACs are significantly correlated, with Pearson’s coefficients above 0.776. This is attributed to the characteristic lower pocket of these HDACs, created by an outward-facing histidine that replaces the canonical tyrosine found in all other HDACs [24]. HDAC11 shares no significant sequence similarity with any other HDAC (sequence identities < 24%). It is a fatty acid deacylase rather than a deacetylase [27]. In accordance with this observation and its classification into a separate HDAC class, the ligand recognition pattern of HDAC11, as indicated by Pearson’s coefficients around zero, is also unique.

### 2.5. A Comparison of Human and Zebrafish HDAC10

The expression of the functional catalytic domain of human HDAC10 in *E. coli* provides enzyme batches with low and varying activities. Also, crystallization has proven to be difficult, yielding low-quality crystals and poor diffraction patterns. Consequently, HDAC10 from zebrafish, drHDAC10, has been established as an alternative, which can be recombinantly produced in high yields and with high activity in *E. coli* [11]. All 35 known 3D structures of HDAC10 have been crystallized using the HDAC10 ortholog from zebrafish. The catalytic domains of drHDAC10 and human HDAC10 share a sequence identity of 65% (and a similarity of 79%), which is much higher than the identity between human class IIb members HDACs 6 and 10 (54%). Therefore, we asked ourselves whether drHDAC10 would be a good functional substitute for human HDAC10 regarding the recognition of structurally diverse HDACis. Indeed, the ligand recognition pattern of drHDAC10 is highly and significantly correlated with that of human HDAC10 (r = 0.779, see Figure 6 and Table S2). Furthermore, a substantial correlation has been observed between the ligand-binding profiles of both HDAC10 and drHDAC10 and those of class IIa HDACs. However, there is some indication of a similarity between the binding pockets of HDAC1 and HDAC2 and drHDAC10, which is not observed for human HDAC10. It is evident that the structurally diverse profile compounds for human and zebrafish HDAC10 exhibit remarkably similar binding patterns. The close relationship between the two enzymes is confirmed by hierarchical clustering of all HDAC homologs. The fact that several well-known HDAC10 inhibitors have now been crystallized in complex with drHDAC10 provides further evidence that the zebrafish enzyme can be used as a surrogate for human HDAC10. Moreover, drHDAC10 has been demonstrated to be more stable and can be produced in greater quantities. However, the similarity of drHDAC10 to HDAC1 and HDAC2 in an HTS could lead to the enrichment of hits that do not inhibit the human enzyme. These false-positive hits can be easily weeded out in a follow-up assay using human HDAC10. Therefore, it is hypothesized that drHDAC10 may serve as a valid functional substitute for human HDAC10 and yield useful hit compounds in high-throughput screening (HTS) campaigns, provided that the number of false-positive results does not increase to a level that would compromise the management of compounds for confirmation testing.

### 2.6. A Comparison of Ligand-Binding Profiles of Bacterial Homologs with Respect to Canonical HDAC Classes

We were also interested in the functional relationship between human HDACs and their homologs from bacteria, which are often classified as APAHs based on multiple sequence alignments. APAHs sometimes do not deacetylate polyamines but rather substrates with acetylated lysines. For example, we have shown that two of the bacterial HDAC homologs from *Pseudomonas aeruginosa* (*P. aeruginosa*), PA0321 and PA1409, are true polyamine deacetylases, while PA3774 is not able to deacetylate acetylated polyamines, although all three APAHs from P. aeruginosa can hydrolyze chemically activated trifluoroacetylated lysine derivatives [13]. HDAH from *Bordetella or Alcaligenes* strain FB188 (DSM 11172) also belongs to the APAH subfamily based on sequence analysis but is only moderately active in catalyzing the deacetylation of acetylpolyamines [2]. On the contrary, HDAH shows significantly higher activity on standard HDAC substrates containing acetylated lysine residues. These substrate specificities translate into distinct recognition patterns for structurally divergent HDACis. In fact, the pIC_50_ values are significantly correlated among the bacterial HDAC homologs (Figure 6). Overall, APAHs show clearly different ligand-binding profiles compared to their human homologs. However, the findings regarding APAHs must be interpreted with caution because only 44 out of 104 IC_50_ values were available. PA0321 shows significant ligand-binding similarity to all class IIa HDACs and HDAC10. Despite these partial similarities, the differences in ligand recognition can be exploited to develop selective inhibitors against bacterial APAHs by starting with known HDACis against human HDACs. Our efforts to develop selective inhibitors against *P. aeruginosa*-derived APAHs resulted in a patent for perfluorinated SAHA analogues, such as LU210 [28,29]. LU210 showed an IC_50_ of 0.52 ± 0.02 µM against HDAH and was only weakly active against the human homologs HDAC1 (IC_50_ = 18 ± 3 µM), HDAC6 (IC_50_ = 12 ± 2 µM), HDAC7 (IC_50_ = 15 ± 2 µM) and HDAC8 (IC_50_ = 5.3 ± 0.8 µM) [30]. Moreover, LU210 was also active against the PA3774, PA0321, and PA1409 from *P. aeruginosa* with IC_50_ values of 0.022 ± 5.3, 0.7 ± 0.2, and 2.6 ± 0.1 µM, respectively [31].

### 2.7. Ligand-Binding Profiles of Single Mutant Variants of Human HDACs

Given the high sequence homology within an HDAC class and the very high conservation of amino acids in the active site, we wondered how the substitution of one or more amino acids would affect ligand recognition and, consequently, alter the binding pocket. In particular, we wanted to analyze whether single amino acid substitutions could cause the corresponding mutant variants to be classified according to the ligand-binding pattern of a different HDAC class. There are prominent examples, such as a gain-of-function variant of HDAC4, in which a histidine residue near the active site, H976, is replaced by a tyrosine. At this position, class IIa HDACs differ significantly from class I enzymes. Wild-type HDAC4 is essentially inactive on acetylated lysine substrates. However, the HDAC4_H976Y_ variant is highly active on these substrates, hence the name “gain-of-function” for this variant [32]. Furthermore, it was shown that this variant binds a hydroxamic acid approximately 10-times more strongly than wild-type HDAC4, suggesting that the binding behavior has also changed significantly due to the substitution of a single amino acid [32]. We analyzed the pivotal role of histidine 976 in HDAC4 for enzyme function and ligand recognition previously in more detail and determined binding constants for five different compounds against histidine exchange mutants with every essential amino acid at this position [33]. We confirmed that binding of HDACis with trifluoromethylketone ZBGs and TMP195, which binds to the lower-selectivity pocket of wild-type HDAC4, is decreased in HDAC4_H976Y_, while binding of hydroxamic acids like SAHA and TSA is enhanced by about 2 orders of magnitude. We also observed that HDAC4 variants in which H976 was exchanged for basic, polar, or small hydrophobic amino acids bound more strongly to BW164, which protrudes into the lower foot pocket [33]. Based on these findings, we proposed using HDAC4_H976A_, which has a constitutively open lower-selectivity pocket and enhanced affinity to subpocket binders, as a tool to enrich for selective lower-pocket-targeting HDAC4 inhibitors in a high-throughput screening setup. In this analysis, which used the full set of profile compounds, we demonstrate that the H976Y variant of HDAC4 significantly alters its binding behavior compared to the wild-type and other class IIa HDACs. We demonstrate that the ligand-binding behavior of HDAC4_H976Y_ is comparable to that of class I HDACs 1, 2 and 3 (r = 0.762, 0.732, and 0.704, respectively; Figure 6). Interestingly, the ligand binding of HDAC4_H976Y_ has become strikingly similar to that of HDAC6 (r = 0.806, *p* < 0.001), which is also highly correlated with HDACs 1, 2, and 3. The other HDAC4 mutant variants, HDAC4_H976A_, HDAC4_L728A_, and HDAC4_P809A_, exhibited a highly significant correlation with wild-type HDAC4 (r = 0.808, 0.802, and 0.758, respectively). The application of the Steiger test reveals a substantial discrepancy between the correlations observed for HDAC1, 2, 3, and HDAC4_H976Y_, and those evidenced for class I HDACs 1, 2, 3, and other HDAC4 variants (Table S3). This finding provides further evidence that supports the altered class I-like binding profile exhibited by HDAC4_H976Y_.

This is in contrast to the non-significant correlation observed between wild-type HDAC4 and HDAC6, with a Pearson’s coefficient of 0.297. Additionally, the ligand recognition pattern of HDAC4_H976Y_ resembles that of HDAC8_C153S_ (r = 0.643) but not of wild-type HDAC8. The other HDAC4 variants demonstrate a high degree of similarity to HDAC4_wt_ and the other class IIa HDACs with regard to their ligand-binding profiles, exhibiting Pearson’s coefficients ranging from 0.758 to 0.963. All HDAC8 variants and the HDAC8_wt_ protein exhibit highly similar ligand-binding patterns (r = 0.545–0.751). However, there are subtle differences. For instance, the binding of the ligand to HDAC8_C153S_ is significantly correlated with that of HDAC1, 2, and 6 (r = 0.617, 0.539, and 0.564, respectively). In contrast, the remaining HDAC8 variants demonstrate no significant correlation with class I HDACs and HDAC6. These variants exhibit similarity to the wild-type HDAC8 and a distinct pattern of ligand recognition.

### 2.8. Identification of High-Yield and High-Stability HDAC11 Variants

The half-life of HDAC11 is brief in mammalian cells. In BEAS-2B cells, this was found to be approximately 4 h [34]. The degradation of HDAC11 is predominantly facilitated by the proteasomal pathway in BEAS-2B cells, as opposed to the lysosomal pathway. However, it was also observed that HDAC11 from BV-2 cell lysate exhibited surprising thermal stability in the CETSA assay [30]. Recombinant HDAC11, expressed in *Sf9* (*Spodoptera frugiperda 9*) *cells* and purified for HTS purposes, demonstrated a high degree of instability, exhibiting a 40% loss of activity after three hours at 37 degrees Celsius [35]. It is noteworthy that no foreign protein, such as BSA, was employed for the purpose of stabilization in the assay. In addition to the conflicting information from the literature regarding HDAC11 stability, we have our own experience with the recombinant production and purification of HDAC11 in *E. coli*. In comparison to the recombinant production of other HDACs, such as HDAC8 and the catalytic domain of HDAC4, we could only obtain relatively small amounts of the target protein—approximately 0.1 milligrams per liter of culture broth. These amounts were also contaminated with other proteins that could not be purified. However, we require substantial amounts of stable HDAC11 to screen extensive drug libraries and conduct biophysical studies on ligand binding. These requirements motivated our research efforts to identify mutant variants of HDAC11 that offer higher production yields and increased stability while retaining the binding site characteristics and binding profile with respect to the reference inhibitors. To this end, we used the PROSS server at the Weizmann Institute to generate predictions for more stable HDAC11 variants based on the wild-type HDAC11 sequence (https://pross.weizmann.ac.il/step/pross-terms/, accessed on 2 February 2026). PROSS is a protein stability design method. The structure AF-Q96DB2-F1 of HDAC11 from the AlphaFold protein structure database served as the input. PROSS suggested nine mutant variants containing an increasing number of amino acid substitutions, ranging from 1 to 43. In accordance with the PROSS User Guide recommendations, we selected three variants, HDAC11 4A, HDAC11 7A, and HDAC11 8A, and produced them recombinantly in *E. coli*. HDAC11 4A had 8 amino acid substitutions, HDAC11 7A had 19, and HDAC11 8A had 29 compared to the wild-type sequence. The sequence information can be found in Figure S6’s mutation map.

**Figure 7:**
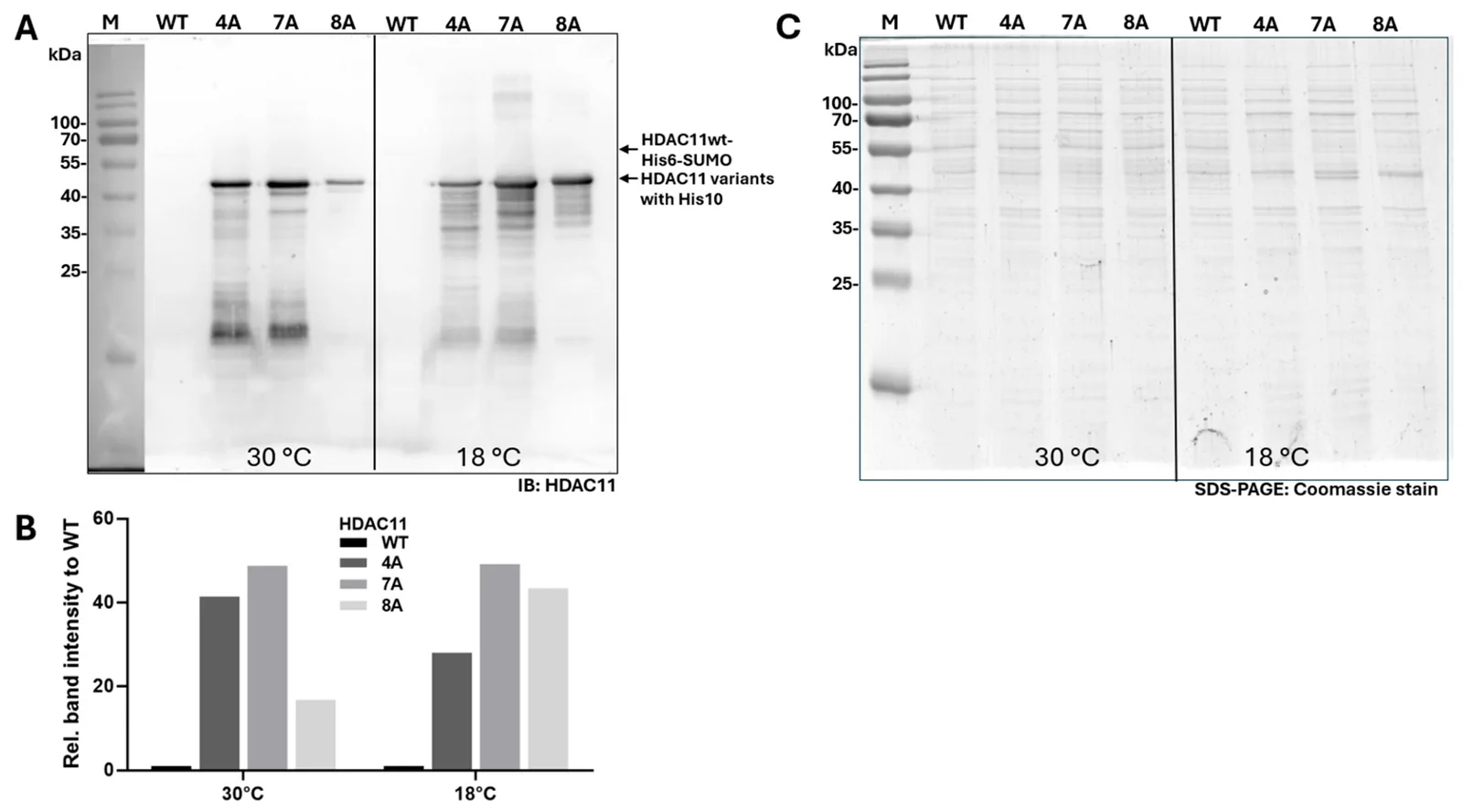
Producibility of HDAC11 variants. (**A**) Western blot of HDAC11_wt_ and its variants produced in *E. coli.* HDAC11_wt_ and variants 4A, 7A, and 8A were produced overnight at 30 °C and 18 °C, respectively. 2 µg of total protein from lysates were loaded per lane. Western blot was probed with a primary antibody against HDAC11. M: Protein molecular weight standard. Arrows point out HDAC11 WT (with His6-SUMO-tag) and mutation variants (with His10-tag). (**B**) Relative HDAC11 band intensities compared to HDAC11_wt_ for 30 and 18 °C determined from (**A**). (**C**) The same gel Coomassie stained to demonstrate the loading of 2 µg of total protein per lane.

**Figure 8:**
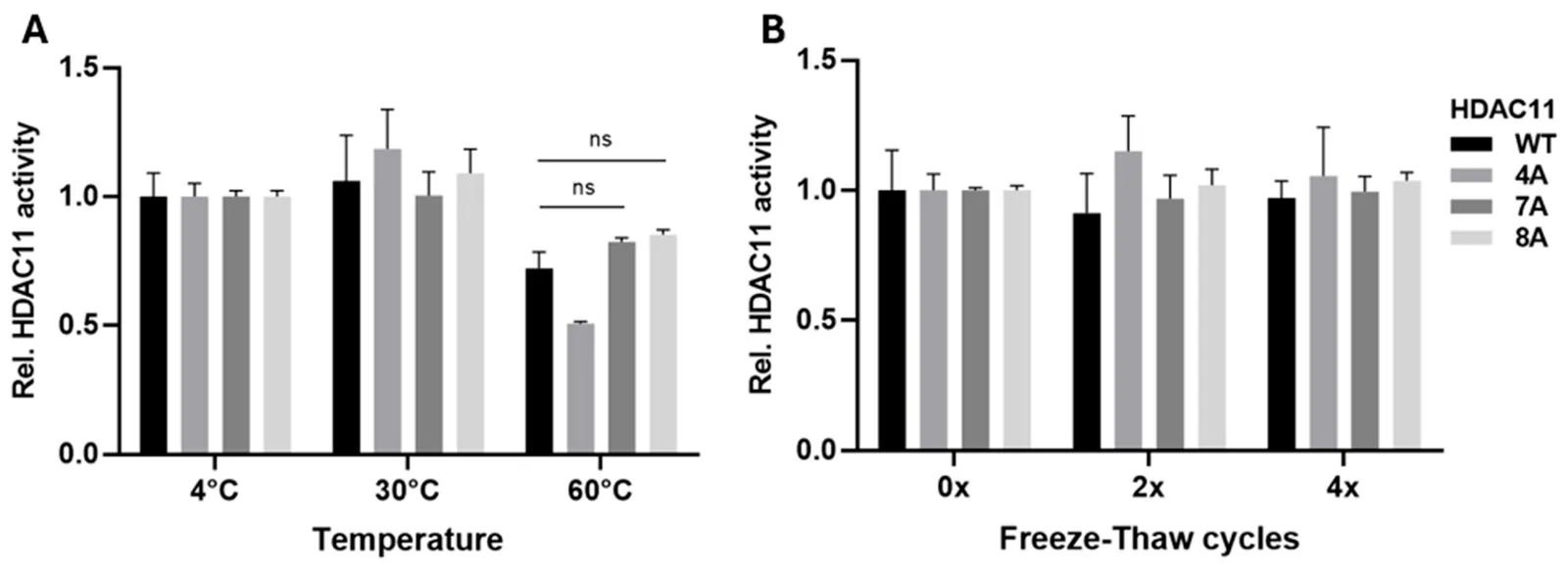
Stability of recombinantly produced indicated HDAC11 variants. (**A**) Thermal stability after incubation for 1 h at indicated temperatures in a buffer containing 150 mM KCl, 50 mM Tris-HCl, 5% glycerol, pH 8.0. ns means no significant difference in relative enzyme activity using 3-way ANOVA. (**B**) Preservation of enzyme activity after indicated number of freeze–thaw cycles. Data points represent means and standard deviations for *n* = 3.

The expression level of HDAC11_wt_ was barely detectable in the cell lysate, when assessed via Western blot, whereas all HDAC11 mutant variants exhibited distinct bands (Figure 7). Due to the presence of faint, barely visible HDAC11_wt_ bands, only approximate estimates could be made regarding the relative excess amounts of the HDAC11 variants relative to the wild-type enzyme. The relative band intensities of the HDAC11 variants in Figure 7B were at least 17-times greater than those of HDAC11_wt_. However, this estimation of the HDAC11 yield relative to total protein was rather conservative and was likely to be higher. Subsequently, the thermal stability of the HDAC11 variants was examined, as well as the effect of multiple freeze–thaw cycles on enzyme activity. Both wild-type HDAC11 and the HDAC11 mutant variants remained fully stable following one hour of incubation at 4 and 30 °C. Following a one-hour incubation at 60 °C, a slight decrease in enzyme activity was observed, with HDAC11_wt_ exhibiting approximately 70% residual activity. In contrast, HDAC11 7A and 8A demonstrated slightly higher residual activities, exceeding 80%. This difference was not statistically significant compared to the enzyme activity of HDAC11_wt_ (Figure 8A). Notably, all HDAC11 variants demonstrated remarkable stability after undergoing up to four freeze–thaw cycles. During these cycles, the enzymes were flash-frozen using liquid nitrogen and then thawed at 30 °C prior to testing (Figure 8B).

**Figure 9:**
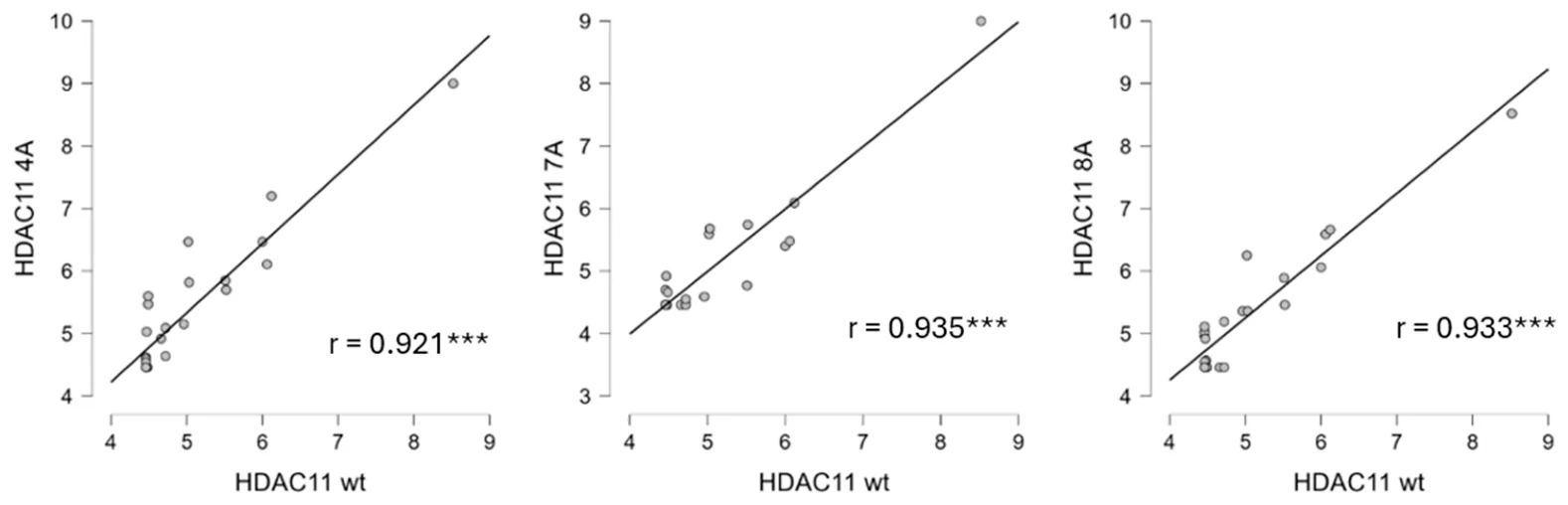
Pairs of pIC_50_ values for the profile compounds are plotted against each other for the indicated HDAC11 variants. Each dot represents an HDACi. r is the Pearson’s correlation coefficient. The starred *p*-values are nominal and exploratory. A Benjamini–Hochberg correction was applied to the data. The adjusted *p*-values and significance for each pair are summarized in Table S4. *** *p* < 0.001.

The observed discrepancy between the high instability of recombinant HDAC11 described by Tian et al. [36], albeit produced in Sf9 insect cells, and the remarkable stability we observed with regard to temperature and freeze–thaw cycles was unexpected. This discrepancy may be explained by the fact that no foreign protein, such as BSA, was added to the activity assay by Tian et al., which could have had a stabilizing effect. We successfully produced HDAC11 recombinantly in *E. coli*; however, we were unable to purify out all foreign proteins. The stabilizing effect of bacterial chaperones and other proteins on a variety of enzymes in solution has been reported in many other studies [37,38,39]. Consequently, we hypothesize that these proteins contributed to the surprising stability of our HDAC11 preparations. Subsequently, an analysis was conducted to ascertain whether the ligand-binding profile of the HDAC11 mutant variants remained consistent with that of the wild-type enzyme. To accomplish this objective, we conducted a comprehensive evaluation of the pIC_50_ values of all reference inhibitors against the HDAC11 variants. We then correlated the obtained data with the pIC_50_ values against the other human zinc-dependent HDACs (Figure 6). It is noteworthy that the Pearson’s correlation coefficients between HDAC11_wt_ and the HDAC11 mutant variants 4A, 7A, and 8A are the highest observed for all correlated HDACs (Figure 9; r = 0.937, r = 0.953, and r = 0.933, respectively). Consequently, the ligand-binding properties of the mutant variants are also very similar to one another. Furthermore, the HDAC11 mutant variants, similar to HDAC11_wt_, exhibit substantial differences in their ligand-binding profiles when compared to all other HDACs. This finding indicates that the binding pocket and active site of all mutant variants must exhibit a high degree of similarity to those of the wild-type enzyme. The HDAC11 7A and HDAC11 8A variants can be produced with greater ease at approximately 1.4 mg and 0.6 mg per liter of culture broth, respectively, compared to HDAC11_wt_ at 0.1 mg per liter of culture broth. Both HDAC11 variants exhibit stability comparable to that of the wild-type enzyme. These characteristics render them particularly well-suited for high-throughput screening campaigns and the biophysical investigation of HDAC11 ligand interactions. However, further refinement is necessary for the purification of the HDAC11 variants in the context of biophysical studies. It is hypothesized that these mutant variants will facilitate protein crystallization and, consequently, the determination of the first experimental structure of HDAC11.

### 2.9. Functional Classification of Zinc-Dependent HDAC Homologs Based on Their Ligand-Binding Profiles

**Figure 10:**
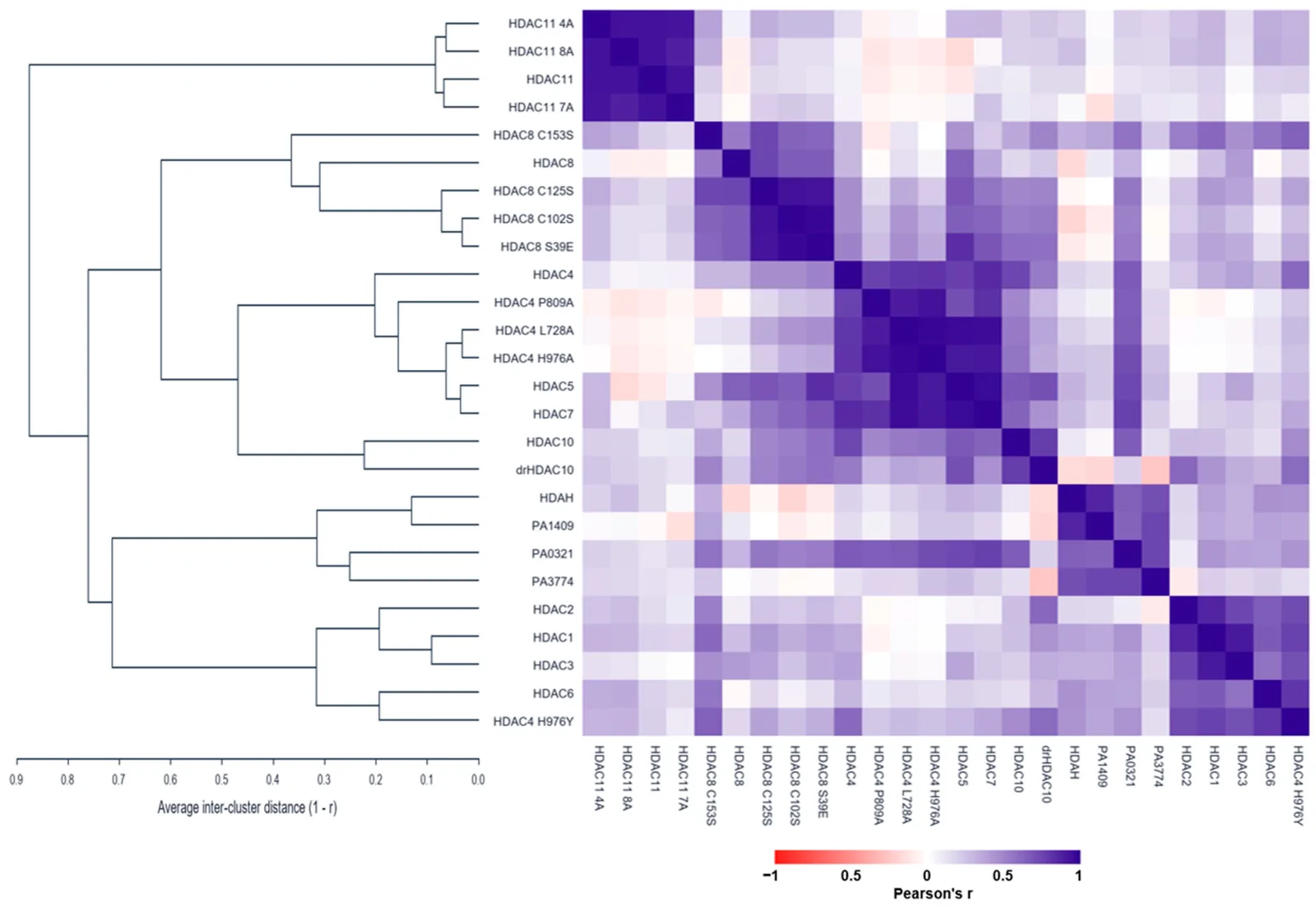
Functional hierarchical clustering of zinc-dependent HDAC homologs, mutant variants and APAHs using their ligand-binding profile. Average linkage was applied using 1-Pearson’s r as pairwise distance. Left panel shows the dendrogram of the corresponding clustered heatmap, which is colored according to Pearson’s r.

Next, we investigated whether all zinc-dependent HDAC homologs could be reliably classified into functional categories based on their ligand-binding profiles. To this end, we performed hierarchical clustering with average linkage and chose 1-Pearson’s r as the distance measure (Figure 10). Hierarchical clustering based on ligand recognition yielded a significantly different functional classification of HDACs than the traditional classification based on sequence similarities. The classification of HDAC homologs, mutant variants, and APAHs is indicative of the similarities in ligand recognition among the various enzymes previously discussed. By grouping HDACs with similar binding behavior together, the classification system reflects the similarities in ligand recognition among the various enzymes. This finding further substantiates the conclusion that HDAC6 and HDAC4_H976Y_ are members of a group that encompasses class I HDACs 1, 2, and 3. In contrast, class I HDAC8 exhibits a substantial divergence from the other class I HDACs. However, in conjunction with the HDAC8 mutant variants, it constitutes a sizable group that is distinct from the other groups. In a similar manner, the bacterial APAHs form a distinct cluster that is different from the other HDACs.

It has previously been established that the ligand-binding profiles of class IIb, 6, and 10 HDACs are uncorrelated. This phenomenon is further substantiated by the functional classification, wherein the two enzymes are observed to be dispersed across distinct clusters. In contrast, human and zebrafish drHDAC10 demonstrate a high degree of similarity in terms of ligand binding, resulting in their classification in the same group. It is also evident that all HDAC11 variants cluster very closely together and differ very clearly from all other HDAC homologs in their ligand-binding behavior.

**Figure 11:**
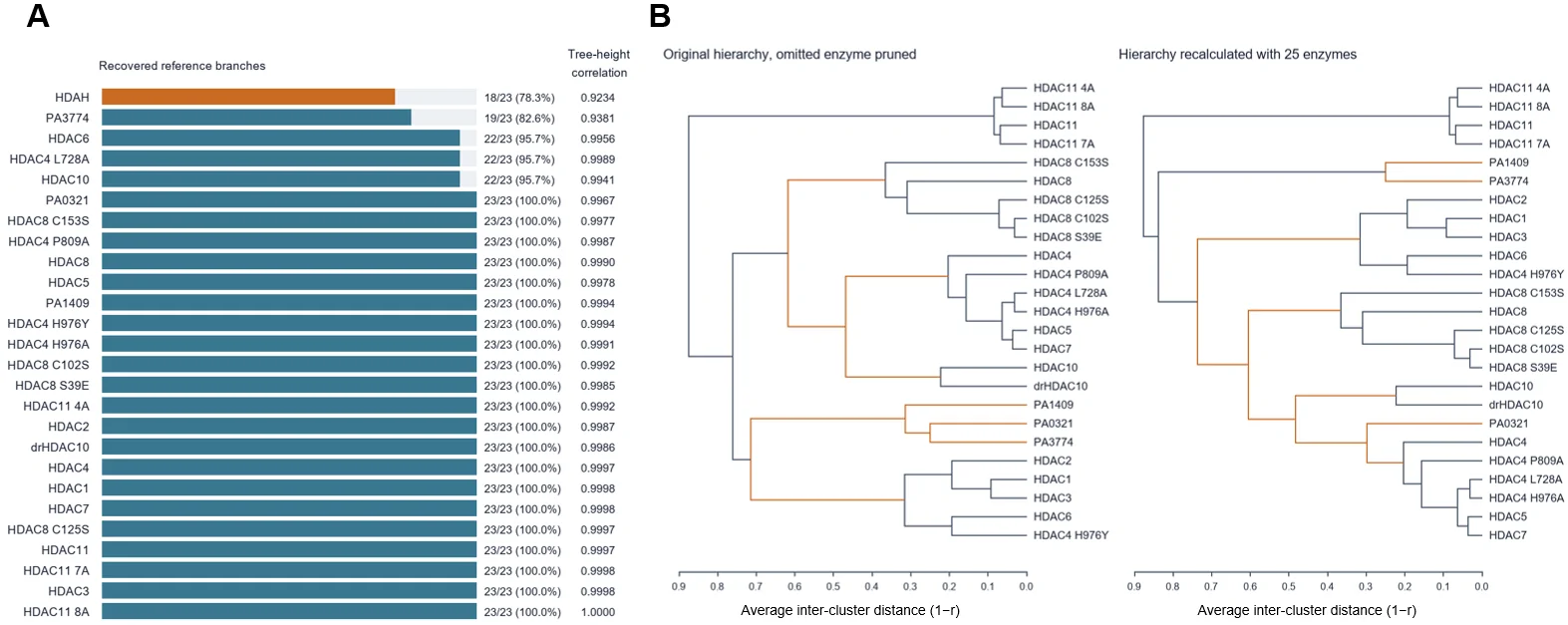
Leave-one-enzyme-out clustering stability. (**A**) Recovered reference branches in the original tree after removing the omitted enzyme. Tree-height correlation compares all surviving enzyme pairs. These are sensitivity measures. (**B**) Largest topology change by omitted HDAH. Recovered branches (18/23), tree-height correlation 0.9234. Orange marks groups absent from the other tree. Black marks shared groups or trivial branches. Each tree uses its own valid leaf order. Branch rotations do not affect the comparison.

The sensitivity of hierarchical clustering to enzyme composition was assessed in 26 leave-one-enzyme-out runs using average linkage and 1-r as the distance measure. Each resulting dendrogram was compared with the original dendrogram pruned to the same 25 remaining enzymes (Figure S8). In 21 of the 26 runs, all reference branches were preserved. Branch recovery ranged from 78% to 100%, while Pearson correlations between the two trees’ pairwise cophenetic heights ranged from 0.9234 to 0.9999 (Figure 11). Omission of HDAH produced the largest topological change, retaining 18 of 23 reference branches (78%) and yielding a between-tree cophenetic correlation of 0.9234 (Figure 11). However, even in this instance, characterized by the least successful recovery of reference branches, the clusters from the original tree are largely maintained. The HDAC11 variants, the HDAC8 variants, and HDAC10 from humans and zebrafish once again demonstrate clear distinctions, forming distinct groups. In a similar manner, the class IIa HDACs persist in their tendency to congregate with all HDAC4 variants, with the exception of HDAC4_H976Y_. As observed in the original tree, the class I HDACs 1, 2, and 3, along with HDAC6 and HDAC4_H976Y_, form a single cluster. It is noteworthy that the bacterial enzyme PA0321 is no longer grouped with the other APAHs; rather, it is assigned to the class IIa cluster, albeit at a slightly greater distance. The data demonstrate the efficacy of classifying HDACs, particularly new enzymes and mutant variants, based on their ligand-binding profiles.

## 3. Materials and Methods

Profile compounds: ACY-738, DKFZ-748, entinostat, FT895, panobinostat, PCI-34051, quisinostat, ricolinostat, romidepsin, SAHA(vorinostat), SS208, TMP195, TMP269, trichostatin A, and tubastatin A were purchased from MedChemExpress (Monmouth Junction, NJ, USA). LU210 [28], P2742 [40], SATFMK [41] and the MB series (MB_643, MB_464, MB_468, MB_469, MB_471, and MB_477) [42] were synthesized in our group, and the synthesis was described in the earlier studies mentioned. PDF361-01 was kindly provided by Dr. Matt Fuchter (University of Oxford, UK). All compounds were at least 95% pure.

Site-directed mutagenesis: SOE-PCR was used to create variants of HDAC4 and HDAC8 with single amino acid mutations, as previously described [43,44].

HDAC11 variants: Human HDAC11 variants were synthesized by Biocat (Heidelberg, Germany) and codon-optimized for *E. coli*. We used the PROSS server from the Weizmann Institute (https://pross.weizmann.ac.il/step/pross-terms/, accessed on 2 February 2026) to generate predictions for more stable HDAC11 variants based on the wild-type HDAC11 sequence (Uniprot Q96DB2) [45]. PROSS is a protein stability design method [45]. The structure AF-Q96DB2-F1 of HDAC11 from the AlphaFold protein structure database served as the input. Nine different HDAC11 designs were generated, each with a different number of mutations, which are expected to be more stable due to the mutated sequences. Three of these nine variants were chosen for synthesis: 4A, which has 8 mutations; 7A, which has 19 mutations; and 8A, which has 29 mutations and has had about 10% of its amino acids mutated (Figure S6).

Enzyme preparation: HDACs 1 (Cat#500551), 2 (Cat#50002), 3 (Cat#50003), 5 (Cat#50005), 6 (Cat#50006), and 7 (Cat#50007) were purchased from BPS Bioscience (San Diego, CA, USA). Human HDAC8, the C-terminal catalytic domain of HDAC4 (residues T648-T1057), HDAC10, HDAC11, and their respective variants were recombinantly expressed in *Escherichia coli* BL21(DE3) cells. HDAC8 and HDAC4 constructs were cloned into pET14b vectors encoding N-terminal His_6_-SUMO fusion proteins and were produced as previously described [33,43,44]. Briefly, for HDAC8 and HDAC4 production, overnight cultures were used to inoculate sterile autoinduction medium containing 3.08 g L^−1^ KH_2_PO_4_, 3.10 g L^−1^ Na_2_HPO_4_, 0.44 g L^−1^ MgSO_4_, 20 g L^−1^ Lennox LB medium, 4.6 g L^−1^ glycerol, 0.45 g L^−1^ glucose, 1.2 g L^−1^ lactose, and 100 μg mL^−1^ ampicillin. Cultures were incubated overnight at 30 °C with shaking. HDAC10 and HDAC11 constructs were produced in LB medium supplemented with 100 μg mL^−1^ ampicillin and induced with IPTG at an OD_600_ of 0.6–0.8 (using 100 μM IPTG for HDAC10 or 200 μM IPTG for HDAC11), followed by overnight incubation at 16 °C. HDAC10 (Uniprot human Q969S8; Uniprot zebrafish F1QCV2) constructs were synthesized as codon-optimized genes and cloned into pET14b vectors encoding N-terminal His_6_-MBP (human HDAC10) or His_6_-SUMO (zebrafish HDAC10) fusion tags. Human HDAC11 and its variants were synthesized as codon-optimized genes. Wild-type HDAC11 was cloned into a pET14b vector containing an N-terminal His_6_-SUMO tag, whereas variants were cloned into pET19b vectors encoding an N-terminal His_10_ tag. Cells were harvested by centrifugation (6000× *g*, 10 min, 4 °C) and resuspended in the appropriate lysis buffer. HDAC8 and HDAC4 samples were lysed in IMAC buffer containing 50 mM Tris-HCl, 150 mM KCl, pH 8.0, supplemented with 5 mM imidazole, 3 μg mL^−1^ DNase I, 5 mM dithiothreitol (DTT), and 0.1 mg mL^−1^ lysozyme. HDAC10 samples were lysed in 50 mM K_2_HPO_4_, 300 mM NaCl, 10 mM MgCl_2_, 10% glycerol, pH 8.0, containing 0.1 mg mL^−1^ lysozyme, 50 μg mL^−1^ DNase I, and HALT protease inhibitor cocktail. HDAC11 samples were lysed similar to HDAC4 and HDAC8 in 50 mM Tris-HCl, 150 mM KCl, pH 8.0, supplemented with 20 mM imidazole, 3 μg mL^−1^ DNase I, and 0.1 mg mL^−1^ lysozyme. Cell disruption was performed by sonication on ice, followed by clarification of lysates by centrifugation at 18,000× *g* for 30 min at 4 °C and filtration through 0.45 μm membranes. All proteins were initially purified by immobilized metal affinity chromatography (IMAC) using Ni^2+^-IDA (hHDAC10, drHDAC10, HDAC11, HDAC11 variants) or cOmplete His-Tag purification column (HDAC8 variants and HDAC4 variants). Bound proteins were washed with the corresponding low-imidazole buffers and eluted with buffers containing up to 500 mM imidazole. For HDAC8 and HDAC4, the His_6_-SUMO fusion tag was removed by incubation with SUMO protease during overnight dialysis at 4 °C against IMAC buffer supplemented with 5 mM DTT. Cleaved His_6_-SUMO tags and remaining contaminants were subsequently removed by reverse IMAC and anion-exchange chromatography (MiniChrom Toyopearl GigaCap Q-650M, Tosoh Bioscience, Griesheim, Germany) for HDAC8 or by hydrophobic interaction chromatography (HiTrap Phenyl HP, Cytiva, Dreieich, Germany) for HDAC4. Final purification and buffer exchange of HDAC8 and HDAC4 were performed by size-exclusion chromatography (SEC) using a HiLoad Superdex 16/600 75 pg column (Cytiva, Dreieich, Germany) equilibrated with SEC buffer consisting of 150 mM KCl, 50 mM Tris-HCl, 1 mM TCEP, and 5% glycerol, pH 8.0. Protein-containing fractions were concentrated using ultrafiltration devices (Vivaspin 2, 30 kDa MWCO, Sartorius, Göttingen, Germany or Amicon Ultra-15, Merck, Darmstadt, Germany). HDAC11 and HDAC10 preparations were concentrated and buffer-exchanged into SEC buffer or buffer C (50 mM HEPES (pH 7.5), 300 mM KCl, 1 mM TCEP, 5% glycerol) for HDAC10 using ultrafiltration (Amicon Ultra-15 centrifugal filter units, Merck, Darmstadt, Germany). All proteins were flash-frozen in liquid nitrogen and stored at −80 °C until use. All chromatographic purification steps were carried out on an ÄKTA Pure FPLC system (GE Healthcare, München, Germany).

Enzyme activity assays: A fluorescence-based HDAC activity assay was used to evaluate wild-type/mutant variants of HDACs. Enzymes (typically 1–100 nM) were preincubated with a serial dilution of test compounds for 1 h at 30 °C in assay buffer containing Tris-HCl (pH 8.0), KCl, and Pluronic F-68 (Sigma Aldrich, Darmstadt, Germany), except for HDAC11 and variants where MAL buffer (50 mM Tris (pH 8.0), 137 mM NaCl, 2.7 mM KCl, 1 mM MgCl_2_, 0.5 mg/ml BSA) was used. Reactions were initiated by adding the appropriate fluorogenic substrate incubated for 60 min and terminated with a stop solution to inhibit HDAC activity. The substrate conversion after 60 min was under 10%, ranging between 1% and 10%, depending on the HDAC used. Specifically, HDAC4 WT/variants, HDAC5, HDAC7, and HDAC8 WT/variants were assayed with 20 µM Boc-Lys(TFA)-AMC (Bachem, Bubendorf, Switzerland) and stopped with 1.67 µM SATFMK; hHDAC10 and drHDAC10 with 20 µM Boc-Lys(TFA)-AMC and stopped with 3.33 µM Quisinostat; HDAC11 WT/variants with 80 µM Boc-Lys(TFA)-AMC and stopped with 3.33 µM FT895 (Sigma-Aldrich, Darmstadt, Germany); and HDAC1-3 (BPS Bioscience (San Diego, CA, USA)), HDAC4 H976Y, and HDAC6 with 50 µM Boc-Lys(Ac)-AMC (Bachem, Bubendorf, Switzerland) and stopped with 4.17 µM SAHA (Cayman Chemical, Ann Arbor, MI, USA). Simultaneously, treatment with 0.417 mg/ml trypsin (AppliChem, Darmstadt, Germany) released the fluorescent AMC reporter, which was quantified using a PHERAstar fluorescence plate reader (excitation 350–360 nm, emission 450–460 nm). Blank controls lacking enzyme were included and subtracted from all measurements. For IC_50_ determination, enzyme activity was measured across inhibitor concentration, normalized to uninhibited controls, and analyzed by fitting dose–response curves to a four-parameter logistic model in GraphPad Prism 8.0.2 (GraphPad Software, San Diego, CA, USA).

Western blot of HDAC11 variants: HDAC11 variants and wild-type HDAC11 were expressed in *E. coli* and lysed as described under enzyme preparation. Protein concentrations in the lysates were determined using a BCA assay kit (Thermo Fisher Scientific, Dreieich, Germany). Samples were mixed with loading buffer (31.5 mM Tris-HCl, pH 6.8; 10% glycerol; 1% SDS; 50 mM DTT; 0.005% bromophenol blue) and denatured at 95 °C for 5 min. For each sample, 2 µg of protein was loaded per lane for Western blotting and 1 µg for the control SDS-PAGE. Samples were separated on 10% acrylamide gels at 200 V for 40 min. Proteins were transferred to a 0.45 µm PVDF membrane (Merck, Darmstadt, Germany) using the Trans-Blot Turbo Transfer System (Bio-Rad Laboratories, Inc., Dreieich, Germany) at 25 V for 30 min. The membrane was then blocked with 3% BSA in PBS for 30 min at room temperature and incubated overnight at 4 °C with an HDAC11 antibody (D5I8E rabbit monoclonal antibody; Cell Signaling Technology, Danvers, MA, USA) diluted 1:1000 in 3% BSA in PBST. After four 5 min washes with PBST, the membrane was incubated for 30 min at room temperature with an HRP-conjugated secondary antibody (donkey anti-rabbit IgG; Thermo Fisher Scientific, Dreieich, Germany) diluted 1:10,000. The membrane was subsequently washed four times with PBST and twice with PBS. Protein bands were visualized using an Amersham™ ECL™ Start substrate (Cytiva, Dreieich, Germany) and a Biostep CELVIN^®^ chemiluminescence imager (Burkhardtsdorf, Germany). A series of images was taken, with the luminescence signal of each image being amplified by addition. The colorimetric marker was captured as a white-light image and subsequently transformed and applied to the chemiluminescence image. The Western blot was semi-quantified using the ImageJ software v. 1.54, with the image being converted to grayscale and the mean grey value of the bands and background being measured. The background value was then subtracted from the mean grey value of the bands and compared to the wild-type measurement.

Thermal stability testing of HDAC11 Variants: Variants were diluted in buffer (150 mM KCl, 50 mM Tris-HCl, 5% glycerol, pH 8.0), transferred to 0.2 mL PCR tubes, and incubated for 1 h at the indicated temperatures in a PCR thermocycler (LabCycler Gradient, SensoQuest, Göttingen, Germany). Afterwards, enzyme activity was measured.

Compound similarity and activity cliff analysis: The similarity of chemical structures of the profile compounds was analyzed using DataWarrior v06.06.04 (https://openmolecules.org/). The program calculates pairwise Tanimoto distances of fingerprints based on SkelSpheres descriptors. Structurally related structures are connected by lines and grouped together in clusters.

Multiple sequence alignment and calculation of sequence identities: Sequences of HDAC isozymes were downloaded from the Uniprot database and aligned using T-Coffee and default parameters [46]. Then, the overhanging sequence fragments of the aligned sequences were removed and the remaining overlapping 358 positions were analyzed further. For this purpose, they were loaded into MOE version 2024.0601 (Chemical Computing Group, Montreal, QC, Canada), and sequence identities were calculated and displayed as a heatmap.

Statistical analysis: IC_50_ values were converted into pIC_50_ values, i.e., the negative decadic logarithm of IC_50_. The pIC_50_ values were loaded into JASP statistical software version 0.96 (University of Amsterdam, Netherlands) to calculate Pearson’s correlation coefficients, *p*-values, frequency distributions, and display correlation plots and heatmaps [47].

Benjamini–Hochberg (BH) Method [48]: The false discovery rates (FDRs) of Pearson’s correlation coefficients were calculated using the p.adjust function in R 4.6.1 statistical software at an FDR threshold of 0.05.

Steiger’s test [49]: The “cocor” package, which is an acronym for “Correlation Robust and Non-parametric” and is a statistical tool available in R 4.6.1, was utilized as a statistical significance test to compare two Pearson’s correlation coefficients.

Hierarchical clustering: Pairwise Pearson’s correlation coefficients were converted to dissimilarities using d_ij_ = 1 − r_ij_. Agglomerative hierarchical clustering was performed using average linkage (unweighted pair-group method with arithmetic mean, UPGMA), whereby the distance between two clusters was defined as the arithmetic mean of all pairwise dissimilarities between their constituent enzymes. Clustering was applied directly to the precomputed dissimilarity matrix using the numerical precision of the supplied coefficients. The resulting dendrogram determined the enzyme order on both axes of the correlation heatmap. Hierarchical clustering was performed using a custom Python 3.14 implementation of average linkage (UPGMA), with NumPy for numerical calculations and openpyxl for importing the Pearson correlation coefficients from Excel.

## 4. Conclusions

This study demonstrates that systematic ligand recognition profiling provides a functional approach for characterizing zinc-dependent HDACs that complements conventional sequence-based classification. Using a chemically diverse inhibitor panel, distinct recognition patterns were identified across human HDAC isoenzymes, bacterial homologs, and mutant variants. While ligand-binding profiles generally reflected established HDAC classes, several important differences emerged. In particular, HDAC1–3 showed highly similar profiles, whereas HDAC8 was distinct, despite belonging to the same class. Conversely, HDAC6 shared substantial ligand recognition similarity with HDAC1–3, despite relatively low sequence identity, while the closely related HDAC6 and HDAC10 displayed markedly different profiles. Thus, sequence similarity alone does not reliably predict ligand recognition, which is strongly influenced by the local architecture and physicochemical properties of the active site.

The applicability of this approach was further demonstrated for HDAC homologs and variants. Zebrafish HDAC10 showed a highly similar ligand recognition profile to human HDAC10, supporting its use as a functional surrogate in structural and screening studies. Bacterial HDAC homologs exhibited distinct profiles compared with human HDACs, highlighting opportunities for the development of species-selective inhibitors. Furthermore, substitution of a single active-site residue in class IIa HDAC4 substantially shifted its ligand recognition profile toward HDAC class I, demonstrating the critical influence of individual residues on inhibitor recognition. Finally, engineered HDAC11 variants exhibited a combination of enhanced recombinant expression and comparable stability with respect to the wild-type enzyme, along with ligand-binding profiles that closely resembled those of the wild-type HDAC11. Consequently, these findings suggest that they have the potential to be valuable tools for future screening, biophysical, and structural studies.

Overall, correlation-based analysis of ligand recognition profiles provides a practical framework for identifying functional relationships among HDACs, evaluating homologs and engineered variants, and revealing differences that may be exploited for selective inhibitor design. Integration of ligand profiling with sequence and structural information may, therefore, facilitate the development of isoenzyme- and species-selective HDAC inhibitors.

## Figures and Tables

**Table 1:** Inhibitory activities in terms of pIC_50_ values of profile compounds against indicated HDAC isozymes. High activities (pIC_50_ ≥ 8) are highlighted in red, medium activities (6.5 ≤ pIC_50_ < 8) in orange, and low activities (6.0 < pIC_50_ < 6.5) in yellow. Lower-activity pIC_50_ values (pIC_50_ ≤ 6.0) are not colored. * Values with an IC_50_ > 35 µM, ** values with an IC_50_ > 50 µM.

	pIC_50_									
	Class I				Class IIa			Class IIb		Class IV
Compound	HDAC1	HDAC2	HDAC3	HDAC8	HDAC4	HDAC5	HDAC7	HDAC6	HDAC10	HDAC11
ACY-738	6.9	6.4	6.6	4.8	6.2		6.0	8.7	4.5 *	4.7
BW164	4.5 *	4.5 *	4.5 *	5.4	5.9			4.8	6.5	4.5 *
DKFZ-748	5.1	6.9	4.7	5.8	4.5 *		4.5 *	4.9		4.5 *
Entinostat	7.3	6.7	7.1	4.5 *	4.5 *			4.5 *	4.5 *	4.5 *
FT895	5.1	4.5 *	4.5 *	4.5 *	4.5 *			5.6	4.5 *	8.5
LU210	4.7	4.3 **	4.3 **	4.6	4.3 **	4.6	4.8	4.9	4.5 *	4.5 *
MB_463	6.7	6.6	7.0	6.5	5.0	5.1	4.9	6.7	4.5 *	5.5
MB_464	6.7	6.6	7.0	6.6	5.3	5.3	5.1	7.1	4.8	6.0
MB_468	6.8	6.4	6.8	6.4	4.6	4.8	4.9	6.4	4.5 *	4.5 *
MB_469	6.7	6.2	7.0	6.0	4.3 **	4.7	4.8	6.4	4.5 *	4.7
MB_471	5.7	5.4	6.0	5.9	4.3 **	4.3	4.3 **	5.5	4.5 *	4.5 *
MB_477	4.4	4.5 *	4.3 **	5.2	4.3 **	4.4	4.8	4.7	4.5 *	4.5 *
P2742	5.4	4.5 *	5.4	8.0	5.1	7.0	6.7	5.2	4.5 *	4.5 *
Panobinostat	8.7	8.4	8.4	6.0	6.6		5.3	8.2	5.6	6.1
PCI-34051	5.5	4.3 **	4.4	7.6	5.0	4.3 **	5.0	4.7	4.5 *	5.5
PDF361-01	4.7	5.0	6.0	6.9	4.5 *			4.5 *	4.5 *	4.5 *
Quisinostat	8.7	8.4	7.9	7.0	8.3		7.3	6.9	8.4	6.1
Ricolinostat	7.1	6.4	7.2	6.3	5.1		4.9	8.3	4.5 *	5.0
Romidepsin	6.7	4.5 *	8.4	8.1	6.8			5.0	4.5 *	4.7
SAHA	7.5	6.8	7.3	5.3	4.6	4.6	4.9	7.0	4.5 *	4.5 *
SATFMK	7.1	6.0	7.9	7.4	8.0	7.2	6.9	6.5	7.9	4.5 *
SS208	5.6	6.1	6.0	6.0	4.5 *	5.2	5.1	6.8	4.5 *	5.0
TMP195	4.5 *	4.5 *	4.8	5.1	7.0			5.5	5.3	4.5 *
TMP269	4.3 **	4.5 *	4.9	5.4	6.7		6.9	6.0	4.5 *	4.5 *
TrichostatinA	9.0	8.4	8.7	6.5	5.9			9.0	4.7	5.0
TubastatinA	5.4	5.0	4.9	5.0	5.6		5.4	7.6	6.3	4.9

## Data Availability

The original contributions presented in this study are included in the article/Supplementary Materials. Further inquiries can be directed to the corresponding author(s).

## Supplementary Materials

The following supporting information can be downloaded at: https://www.mdpi.com/article/10.3390/ijms27219595/s1. References [30,33,40,50,51] are cited in the supplementary materials.
