# Supplementary material for: Functional Classification of Zn2+-Dependent Histone Deacetylase Homologs and Variants Using Chemical Profiling
Source: Int J Mol Sci. 2026 Oct 27;27(21):9595. doi: 10.3390/ijms27219595 (PMC13763078; doi:10.3390/ijms27219595)
Supplement: Supplementary file 1 [file ijms-27-09595-s001.zip › ijms-4628913-supplementary.pdf]

#Supporting Information

Functional classification of Zn^{2+}-dependent histone deacetylase homologs and variants using chemical profiling

Aleksandra Kopranovic[12], Hannah Münster[12], Aaron Rosowski[12], Louis Groll[12], Florian Almstätter[12], Eva Attenhauser[12], Franz-Josef Meyer-Almes[12*]

[1] Department of Chemical Engineering and Biotechnologiy, Darmstadt University of Applied Sciences, Haardtring 100, 64295 Darmstadt, Germany;
[2] European University of Technology, European Union
• Correspondence: franz-josef.meyer-almes@h-da.de

Content:

Figure S1: Structural clustering of profile compounds
Figure S2: Overlay of dose-response curves for all reference compounds
Table S1: Complete set of IC_{50}-values used for analysis
Figure S3: Heatmap of Pearson's coefficients for the correlation of inhibition profiles
Figure S4: Heatmap of sequence identities between HDAC isozymes
Figure S5: Correlation of pIC_{50} values of profile compounds for all pairs of HDACs
Figure S6: Mutation map of HDAC11 variants
Figure S7: Comparison of heatmaps of Pearson's r and Spearman' rho coefficients
Table S2: Pearson's r and Spearman's rho coefficients, and 95% confidential intervals of chemical profiles for all pairs of HDACs, HDAC variants and APAHs
Table S3: Steiger Tests of correlations between class I HDACs and HDAC4 variants
Table S4: Benjamini-Hochberg correction across all unique correlation pairs at FDR = 0.05
Figure S8: Leave-one-enzyme-out trees resulting from hierarchical clustering
Table S5: Descriptive statistics of pIC50 data
Table S6: Docking scores for the indicated HDACs and their respective ligands.
Figure S9: Predicted binding modes for trichostatin A, panobinostat, and MB-463
Table S7: K_M-values and substrate concentrations used in the enzyme activity assays
Figure S10: Korrelation of Pearson's r from pIC_{50} and pK_i (Cheng-Prusoff corrected)
Figure S11: Protein gels and Western Blots of self-produced HDACs

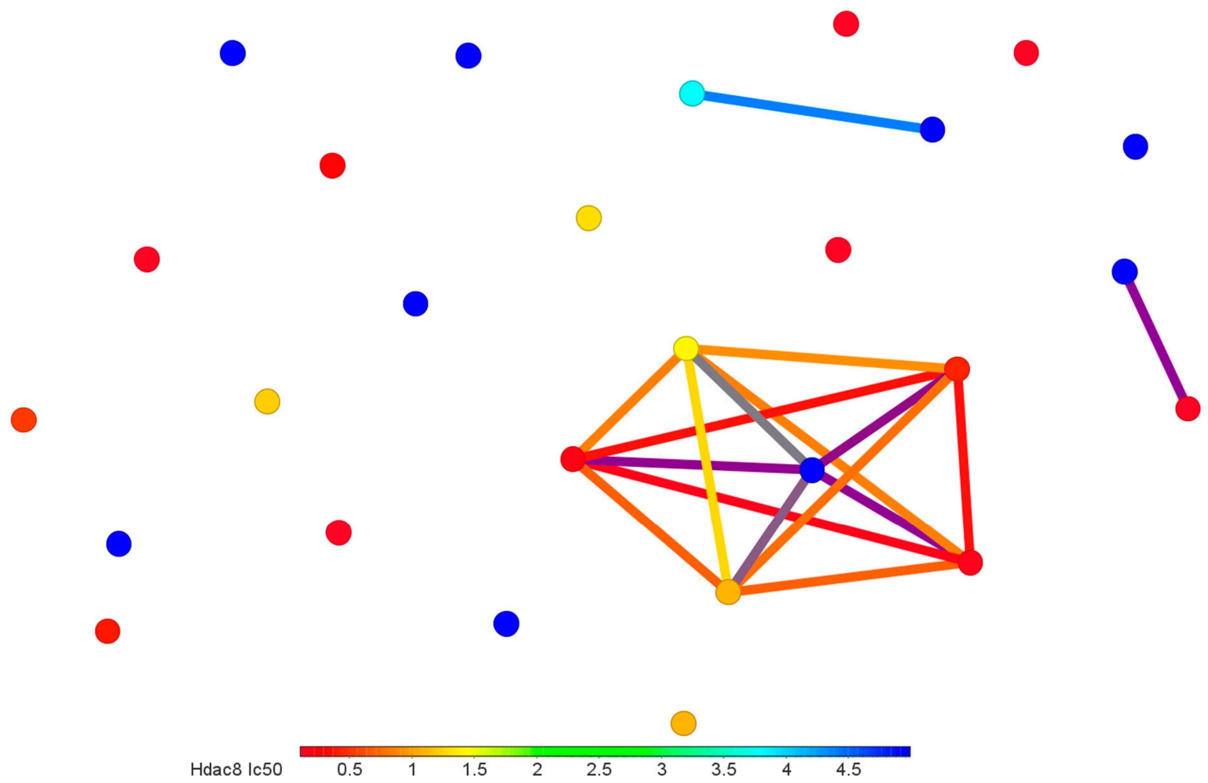

Figure S1: Structural clustering of profile compounds using the Tanimoto similarity based on SkelSpheres fingerprints calculated from DataWarrior software. Each circle represents one profile compound. The colors indicate IC50-values against HDAC8 in µM. Similar structures are connected by lines forming 1 large cluster containing the MB-series compounds and two clusters with two members each. Most compound structures are dissimilar and have Tanimoto coefficients < 0.8.

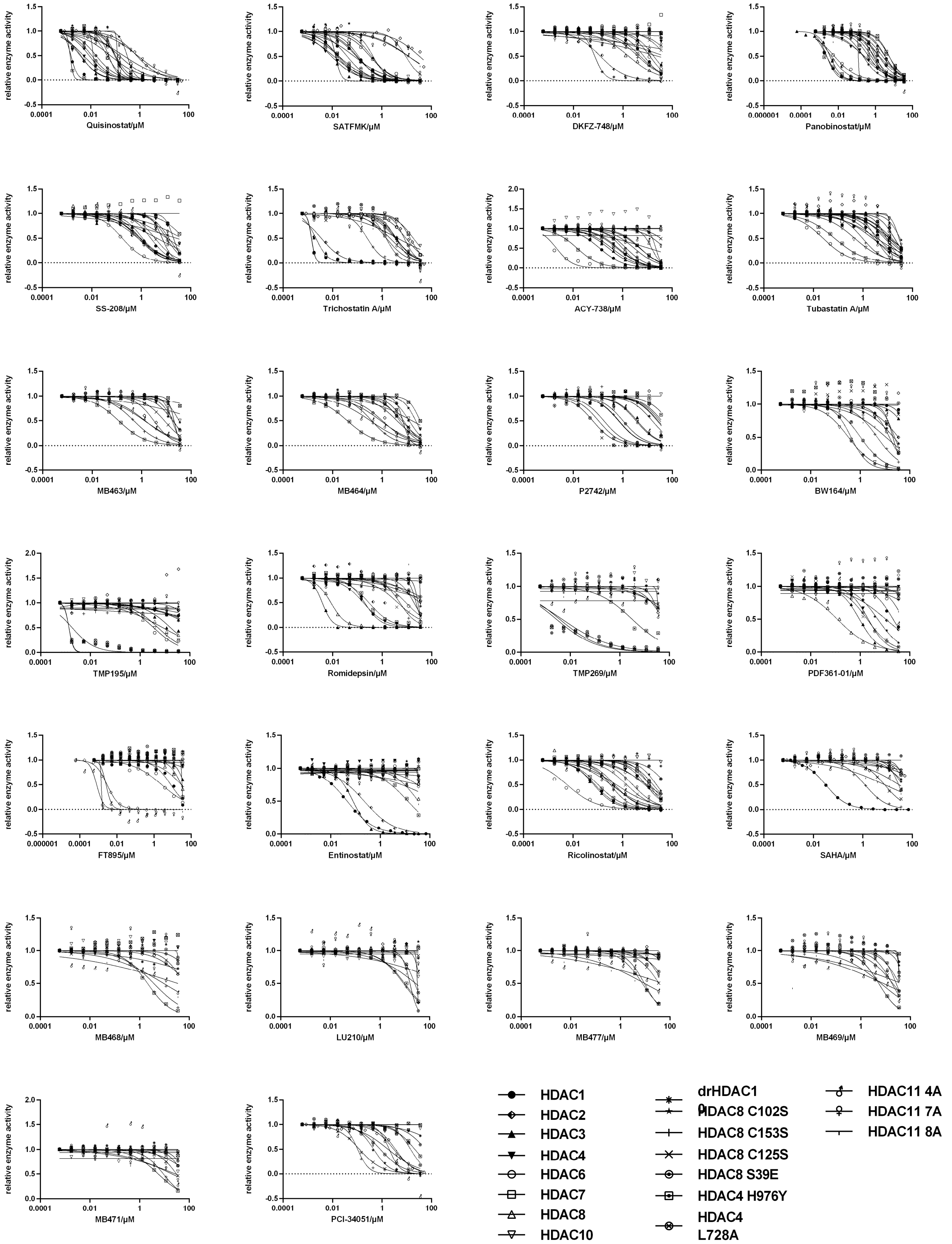

Figure S2: Overlay of dose-response curves for all reference compounds against all investigated HDACs. The figure is characterized by its high resolution of 600 dots per inch (dpi), which allows for significant enlargement to facilitate the observation of minute details.

Table S1: Complete set of IC50-values used for analysis

A) Human HDACs

pIC50 [95%CI]	HDAC1	HDAC2	HDAC3	HDAC8	HDAC4	HDAC5	HDAC7	HDAC6	HDAC10	drHDAC10	HDAC11
**ACY-738**	6.92 [6.96; 6.89]*	6.43 [6.48; 6.38]*	6.55 [6.60; 6.51]*	4.82 [4.89; 4.74]**	6.17 [6.23; 6.15]**	n.d.	6.00 [6.05; 5.92]*	8.70 [9.00; 8.52]*	<4.4	5.30 [5.37; 5.23]**	4.66 [4.72; 4.59]**
**BW164**	<4.4	<4.4	<4.4	5.38[e]	5.89[d]	n.d.	n.d.	4.82 [4.89; 4.74]*	6.47 [6.59; 6.37]*	4.66 [4.80; 4.44]**	<4.4
**DKFZ-748**	5.15 [5.22; 5.08]*	6.92 [7.00; 6.82]*	4.74 [4.89; 4.60]*	5.77 [5.92; 5.62]*	<4.4	n.d.	<4.4	4.92 [5.00; 4.82]*	n.d.	7.14 [7.19; 7.09]*	<4.4
**Entinostat**	7.34 [7.38; 7.30]**	6.66 [6.75; 6.55]*	7.10 [7.15; 7.06]*	<4.4	<4.4	n.d.	n.d.	<4.4	<4.4	<4.4	<4.4
**FT895**	5.10 [5.32; 4.89]*	<4.4	<4.4	4.52 [4.74; <4.4]*	<4.4	n.d.	n.d.	5.59 [5.82; 5.35]*	<4.4	<4.4	8.52 [8.52; 8.52]**
**LU210**	4.74[a]	4.3[a]	4.30[a]	4.57[a]	4.30[a]	4.58[a]	4.83[a]	4.92[a]	<4.4	<4.4	<4.4
**MB_463**	6.68[a]	6.40[a]	6.96[a]	6.49[a]	5.00[a]	5.13[a]	4.92[a]	6.66[a]	<4.4	4.74 [4.82; 4.66]**	5.51 [5.70; 5.31]**
**MB_464**	6.70[a]	6.57[a]	7.00[a]	6.64[a]	5.30[a]	5.34[a]	5.08[a]	7.10[a]	4.80 [5.02; <4.4]**	4.92 [5.00; 4.80]**	6.00 [6.22; 5.80]**
**MB_468**	6.80[a]	6.37[a]	6.82[a]	6.39[a]	4.55[a]	4.82[a]	4.85[a]	6.42[a]	<4.4	<4.4	<4.4
**MB_469**	6.68[a]	6.19[a]	6.96[a]	5.96[a]	4.30[a]	4.66[a]	4.77[a]	6.39[a]	<4.4	<4.4	4.72 [4.82; 4.55]**
**MB_471**	5.70[a]	5.41[a]	6.00[a]	5.85[a]	4.30[a]	4.30[a]	4.30[a]	5.51[a]	<4.4	<4.4	<4.4
**MB_477**	4.38[a]	4.51[a]	4.30[a]	5.18[a]	4.30[a]	4.44[a]	4.80[a]	4.74[a]	<4.4	<4.4	<4.4
**P2742**	5.44[f]	4.5[f]	5.43 [5.66; 5.22]*	7.96[f]	5.07[f]	6.96[f]	6.66[f]	5.17[f]	<4.4	4.82 [4.92; 4.74]**	4.49 [4.52; <4.4]**
**Panobinostat**	8.70 [8.70; 8.52]**	8.4 [8.53; 8.20]*	8.40 [8.52; 8.40]*	5.96 [6.05; 5.92]**	6.64 [6.70; 6.55]**	n.d.	5.26 [5.30; 5.22]*	8.22 [8.40; 8.00]*	5.62 [5.72; 5.52]*	6.66 [6.77; 6.54]**	6.06 [6.21; 5.92]**
**PCI-34051**	5.52[b]	4.35[b]	4.42[b]	7.62[b]	5.00[b]	4.30[b]	5.01[b]	4.74[b]	<4.4	<4.4	5.52 [5.62; 5.43]
**PDF361-01**	4.68 [4.77; 4.55]*	4.96 [5.11; 4.83]*	6.04 [6.11; 5.96]*	6.92 [7.05; 6.80]*	<4.4	n.d.	n.d.	<4.4	<4.4	<4.4	<4.4
**Quisinostat**	8.70 [9.00; 8.61]*	8.40 [8.50; 8.31]*	7.92 [8.40; 7.64]*	6.99 [7.04; 6.95]**	8.30 [8.40; 8.22]**	n.d.	7.33 [7.41; 7.25]*	6.85 [6.89; 6.80]*	8.40 [8.52; 8.30]*	8.05 [8.10; 8.00]*	6.12 [6.25; 6.01]**
**Ricolinostat**	7.11 [7.15; 7.08]*	6.39 [6.49; 6.30]*	7.17 [7.22; 7.11]*	6.32 [6.51; 6.13]*	5.11 [5.21; 5.01]**	n.d.	4.92 [4.96; 4.89]*	8.30 [8.40; 8.05]*	<4.4	<4.4	5.02 [5.30; 4.66]**
**Romidepsin**	6.72 [6.85; 6.59]*	<4.4	8.40 [8.40; 8.40]*	8.10 [8.15; 8.00]*	6.77 [6.82; 6.70]*	n.d.	n.d.	5.05 [5.18; 4.89]*	4.49 [4.66; <4.4]**	<4.4	4.72 [4.82; 4.60]**
**SAHA**	7.51 [7.54; 7.47]**	6.77[a]	7.27[a]	5.28[a]	4.57[a]	4.62[a]	4.92[a]	7.05[a]	<4.4	<4.4	<4.4
**SATFMK**	7.11 [7.26; 6.96]*	6.01[a]	7.85 [8.00; 7.74]*	7.44[a]	8.00 [8.10; 7.92]*	7.23[a]	6.92 [7.00; 6.89]*	6.47 [6.59; 6.35]*	7.92 [8.05; 7.82]**	7.02 [7.12; 6.92]**	4.47 [4.62; <4.4]**
**SS208**	5.62 [5.72; 5.51]*	6.13 [6.23; 6.03]*	6.02 [6.24; 5.81]*	5.96 [6.12; 5.77]*	<4.4	n.d.	n.d.	6.77 [6.85; 6.68]*	<4.4	6.04 [6.08; 6.00]**	4.96 [5.15; 4.74]**
**TMP195**	<4.4	<4.4	4.77 [4.96; 4.54]*	5.08 [5.24; 4.89]*	7.00[d]	n.d.	n.d.	5.46 [5.62; 5.27]*	5.28 [5.42; 5.12]**	<4.4	<4.4
**TMP269**	4.30[c]	<4.4	4.92[c]	5.42[c]	6.66[c]	n.d.	6.92[c]	5.96[c]	<4.4	<4.4	<4.4
**Trichostatin A**	9.00 [9.00; 8.70]*	8.4 [8.66; 8.24]*	8.70 [8.70; 8.52]*	6.52[e]	5.85[d]	n.d.	n.d.	9.00 [9.00; 8.52]*	4.72 [4.85; 4.60]**	5.44 [5.59; 5.31]**	5.03 [5.17; 4.89]**
**Tubastatin A**	5.43 [5.66; 5.19]*	5.03 [5.23; 4.80]*	4.92 [5.14; 4.64]*	5.05 [5.10; 5.00]**	5.55 [5.62; 5.48]**	n.d.	5.38 [5.51; 5.24]*	7.60 [7.70; 7.51]*	6.33 [6.49; 6.16]*	4.96 [5.00; 4.85]**	4.89 [4.96; 4.59]**

a. Meyners, C., and Meyer-Almes, F. J. (2017) Impact of binding mechanism on selective inhibition of histone deacetylase isoforms, *Chemical biology & drug design 90*, 1215–1225.

b. Kleinschek, A., Meyners, C., Digiorgio, E., Brancolini, C., and Meyer-Almes, F. J. (2016) Potent and Selective Non-hydroxamate Histone Deacetylase 8 Inhibitors, *ChemMedChem 11*, 2598–2606.

c. Tilekar, K., Hess, J. D., Upadhyay, N., Schweipert, M., Flath, F., Gutierrez, D. A., Loiodice, F., Lavecchia, A., Meyer-Almes, F.-J., Aguilera, R. J., and Ramaa, C. S. (2021) HDAC4 Inhibitors with Cyclic Linker and Non-hydroxamate Zinc Binding Group: Design, Synthesis, HDAC Screening and in vitro Cytotoxicity evaluation, *ChemistrySelect* 6, 6748–6763.

d. Schweipert, M., Nehls, T., Wurster, E., Boltner, J., Anton, K., Lammer, P., Lermyte, F., and Meyer-Almes, F. J. (2024) The pivotal role of histidine 976 in human histone deacetylase 4 for enzyme function and ligand recognition, *Bioorg. Chem. 153*, 107883.

e. Schweipert, M., Amurthavasan, A., and Meyer-Almes, F. J. (2023) Continuous enzyme activity assay for high-throughput classification of histone deacetylase 8 inhibitors, Explor Target Antitumor Ther 4, 447–459.

B) HDAC mutant variants

pIC50 [95%CI]	HDAC8 C102S	HDAC8 C153S	HDAC8 C125S	HDAC8 S39E	HDAC4 H976Y	HDAC4 L728A	HDAC4 P809A	HDAC4 H976A	HDAC11 4A	HDAC11 7A	HDAC11 8A
**ACY-738**	<4.4	5.52 [5.59; 5.46]*	<4.4	<4.4	7.82 [7.89; 7.80]*	6.22 [6.26; 6.19]*	5.28 [5.34; 5.22]*	5.85 [6.00; 5.70]*	4.92 [5.64; <4.4]*	<4.4	<4.4
**BW164**	<4.4	5.31 [5.40; 5.22]*	<4.4	<4.4	<4.4	6.00 [6.05; 5.92]*	4.68 [5.11; <4.4]*	6.37 [6.48; 6.25]*	4.62 [4.77; <4.4]*	<4.4	4.96 [5.24; 4.60]*
**DKFZ-748**	<4.4	5.39 [5.48; 5.30]*	<4.4	<4.4	5.29 [5.33; 5.25]*	<4.4	<4.4	<4.4	4.59 [5.19; <4.4]*	<4.4	<4.4
**Entinostat**	<4.4	<4.4	<4.4	<4.4	4.89 [5.04; 4.70]*	<4.4	<4.4	<4.4	<4.4	<4.4	<4.4
**FT895**	<4.4	4.52 [4.66; <4.4]*	<4.4	<4.4	4.49 [4.66; <4.4]*	<4.4	<4.4	4.49 [4.60; <4.4]*	9.00 [9.00; 9.00]*	9.00 [9.00; 8.70]*	8.52 [8.70; 8.40]*
**LU210**	<4.4	5.06 [5.12; 5.00]*	<4.4	<4.4	5.00 [5.08; 4.92]*	4.60 [4.64; 4.55]*	<4.4	4.80 [4.85; 4.72]*	<4.4	<4.4	4.57 [4.85; <4.4]*
**MB_463**	4.49 [4.55; <4.4]*	6.32 [6.37; 6.27]*	5.08 [5.17; 5.00]*	4.72 [4.77; 4.64]*	6.92 [6.96; 6.89]*	4.80 [4.80; 4.60]*	<4.4	4.70 [4.80; 4.60]*	5.85 [6.10; 5.64]*	4.77 [4.92; 4.62]*	5.89 [6.08; 5.70]*
**MB_464**	4.64 [4.70; 4.57]*	6.42 [6.46; 6.39]*	5.20 [5.28; 5.13]*	5.13 [5.19; 5.07]*	7.41 [7.48; 7.34]*	5.28 [5.38; 5.19]*	4.47 [4.59; <4.4]*	5.16 [5.21; 5.11]*	6.47 [6.77; 6.19]*	5.40 [5.62; 5.19]*	6.06 [6.24; 5.85]*
**MB_468**	<4.4	5.43 [5.64; 5.19]*	<4.4	<4.4	5.68 [5.77; 5.60]*	<4.4	<4.4	<4.4	<4.4	<4.4	5.03 [5.25; 4.74]*
**MB_469**	<4.4	5.28 [5.37; 5.19]*	4.74 [4.80; 4.70]*	4.57 [4.62; 4.51]*	5.28 [5.41; 5.14]*	<4.4	<4.4	<4.4	5.09 [5.64; <4.4]*	<4.4	5.19 [5.89; <4.4]*
**MB_471**	<4.4	5.04 [5.10; 4.96]*	<4.4	<4.4	5.24 [5.33; 5.17]*	<4.4	<4.4	<4.4	<4.4	4.70 [5.11; <4.4]*	4.55 [5.10; <4.4]*
**MB_477**	<4.4	5.03 [5.12; 4.96]*	<4.4	<4.4	5.11 [5.19; 5.03]*	<4.4	<4.4	<4.4	<4.4	<4.4	5.02 [5.37; 4.51]*
**P2742**	6.18 [6.47; 5.82]*	6.33 [6.42; 6.09]*	6.89 [7.30; 6.64]*	6.70 [7.10; 6.42]*	<4.4	7.21 [7.27; 7.14]*	4.68 [4.85; <4.4]*	5.96 [6.02; 5.92]*	5.47 [5.70; 5.24]*	4.66 [4.80; 4.52]*	<4.4
**Panobinostat**	5.48 [5.55; 5.41]*	6.92 [7.15; 6.89]*	6.21 [6.28; 6.15]*	5.80 [5.89; 5.60]*	8.70 [8.70; 8.52]*	6.36 [6.43; 6.28]*	5.26 [5.34; 5.17]*	5.82 [5.89; 5.74]*	6.11 [6.34; 5.89]*	5.48 [5.70; 5.24]*	6.59 [6.80; 6.39]*
**PCI-34051**	6.34 [6.39; 6.28]*	7.00 [7.05; 6.89]*	6.92 [6.96; 6.85]*	6.07 [6.16; 6.00]*	4.67 [4.74; 4.59]*	4.85 [4.96; 4.72]*	<4.4	<4.4	5.70 [6.15; 5.48]*	5.74 [6.05; 5.48]*	5.46 [5.68; 5.25]*
**PDF361-01**	5.29 [5.39; 5.20]*	<4.4	5.72 [5.89; 5.57]*	5.85 [5.96; 5.42]*	<4.4	<4.4	<4.4	<4.4	<4.4	<4.4	<4.4
**Quisinostat**	6.52 [6.62; 6.43]*	6.92 [7.40; 6.62]*	7.28 [7.30; 7.24]*	7.03 [7.09; 6.97]*	9.00 [9.00; 8.70]*	7.96 [8.00; 7.92]*	7.00 [7.10; 6.85]*	7.77 [7.82; 7.74]*	7.20 [7.55; 6.85]*	6.09 [6.27; 5.92]*	6.66 [6.74; 6.59]*
**Ricolinostat**	4.64 [4.68; 4.57]*	6.49 [6.59; 6.40]*	5.82 [5.92; 5.74]*	4.77 [4.92; 4.59]*	7.00 [7.05; 6.92]*	5.07 [5.19; 4.92]*	4.89 [5.00; 4.77]*	<4.4	6.47 [6.66; 6.28]*	5.59 [5.72; 5.47]*	6.25 [6.48; 6.03]*
**Romidepsin**	<4.4	5.29 [5.38; 5.20]*	5.38 [5.52; 5.21]*	<4.4	6.54 [6.66; 6.42]*	<4.4	<4.4	<4.4	4.64 [4.96; <4.4]*	4.55 [4.72; <4.4]*	<4.4
**SAHA**	<4.4	5.92 [5.96; 5.85]*	5.12 [5.21; 5.02]*	4.55 [4.64; 4.44]*	6.34[d]	<4.4	<4.4	<4.4	4.54 [4.89; <4.4]*	<4.4	5.11 [5.28; 4.92]*
**SATFMK**	6.36 [6.54; 6.14]*	6.41 [6.46; 6.36]*	7.00 [7.05; 6.92]*	7.39 [7.51; 7.27]*	7.31[d]	7.80 [7.82; 7.74]*	7.72 [7.82; 7.64]*	7.72 [7.77; 7.70]*	5.03 [5.40; 4.72]*	4.92 [5.33; <4.4]*	4.92 [5.06; 4.72]*
**SS208**	4.89 [4.96; 4.80]*	6.12 [6.19; 6.06]*	5.44 [5.59; 5.31]*	4.96 [5.02; 4.92]*	6.07 [6.10; 6.03]*	4.74 [4.77; 4.62]*	<4.4	4.70 [4.77; 4.62]*	5.15 [5.44; 4.89]*	4.59 [4.96; <4.4]*	5.36 [5.54; 5.17]*
**TMP195**	<4.4	<4.4	<4.4	<4.4	5.42[d]	8.70 [9.00; 8.22]*	9.00 [9.00; 8.52]*	9.00 [9.00; 8.52]*	<4.4	<4.4	<4.4
**TMP269**	<4.4	<4.4	<4.4	<4.4	5.49 [5.68; 5.30]*	8.30 [9.00; 7.82]*	8.40 [9.00; 7.96]*	8.52 [9.00; 8.05]*	<4.4	<4.4	<4.4
**Trichostatin A**	5.42 [5.54; 5.30]*	6.60 [6.68; 6.51]*	5.44 [5.59; 5.31]*	5.82 [5.92; 5.74]*	7.46[d]	5.10 [5.36; 4.85]*	4.85 [4.96; 4.77]*	5.47 [5.57; 5.37]*	5.82 [6.30; 5.40]*	5.68 [6.10; 5.34]*	5.36 [5.54; 5.17]*
**Tubastatin A**	4.49 [4.57; <4.4]*	6.01 [6.07; 5.96]*	5.12 [5.20; 5.04]*	4.72 [4.82; 4.62]*	7.14 [7.20; 7.09]*	4.72 [4.82; 4.62]*	4.82 [4.92; 4.74]*	4.72 [4.82; 4.62]*	5.47 [5.68; 5.26]*	4.54 [5.00; <4.4]*	5.60 [5.77; 5.44]*

Data are pIC_{50}-values determined from dose-response curves with 10 different concentrations. Data in square brackets define the 95% confidence interval of the corresponding pIC_{50}-value determined either from one experiment * (n=1) or from three independent experiments ** (N=3).

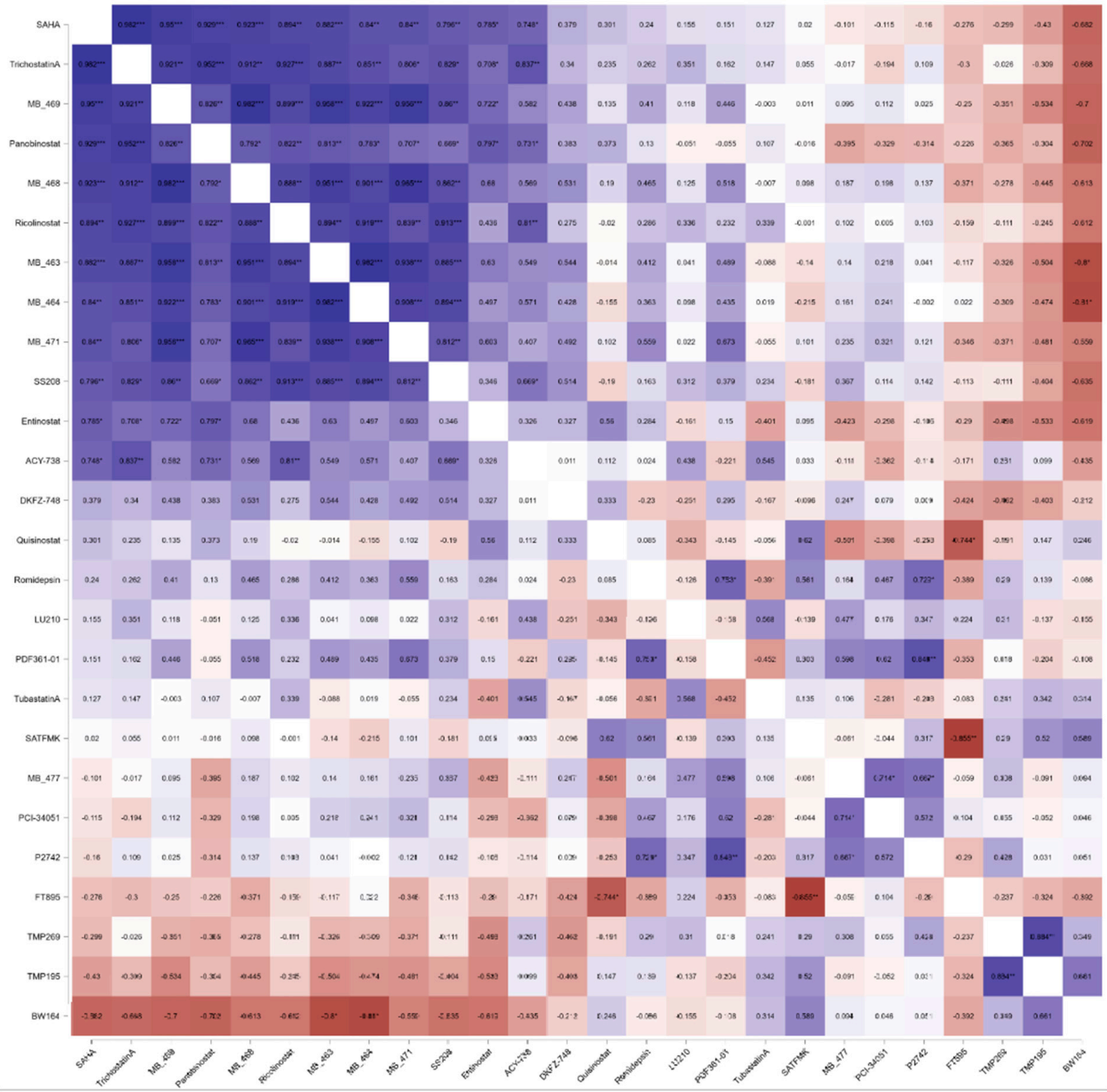

Figure S3: Heatmap of Pearson's coefficients for the correlation of inhibition profiles. To this end pIC50-values of indicated compounds against all human HDAC isozymes were correlated. High Pearson's coefficient in blue indicate similar ligand-recognition patterns by the entire panel of HDACs. Blue color means high correlation, i.e. similar inhibition pattern, while red color indicates negative Pearson's coefficients. * p < .05, ** p < .01, *** p < .001.

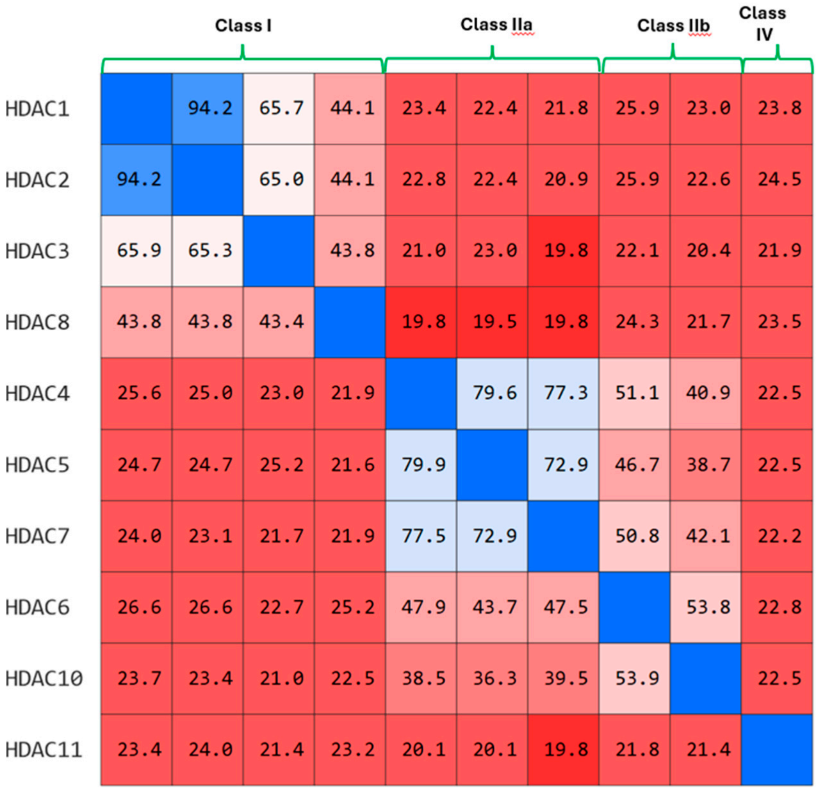

Figure S4: Heatmap of sequence identities between HDAC isozymes based on multiple sequence alignment of the catalytic domain over 358 positions (see also Fig. 4. The sequences were assigned to the corresponding HDAC classes.

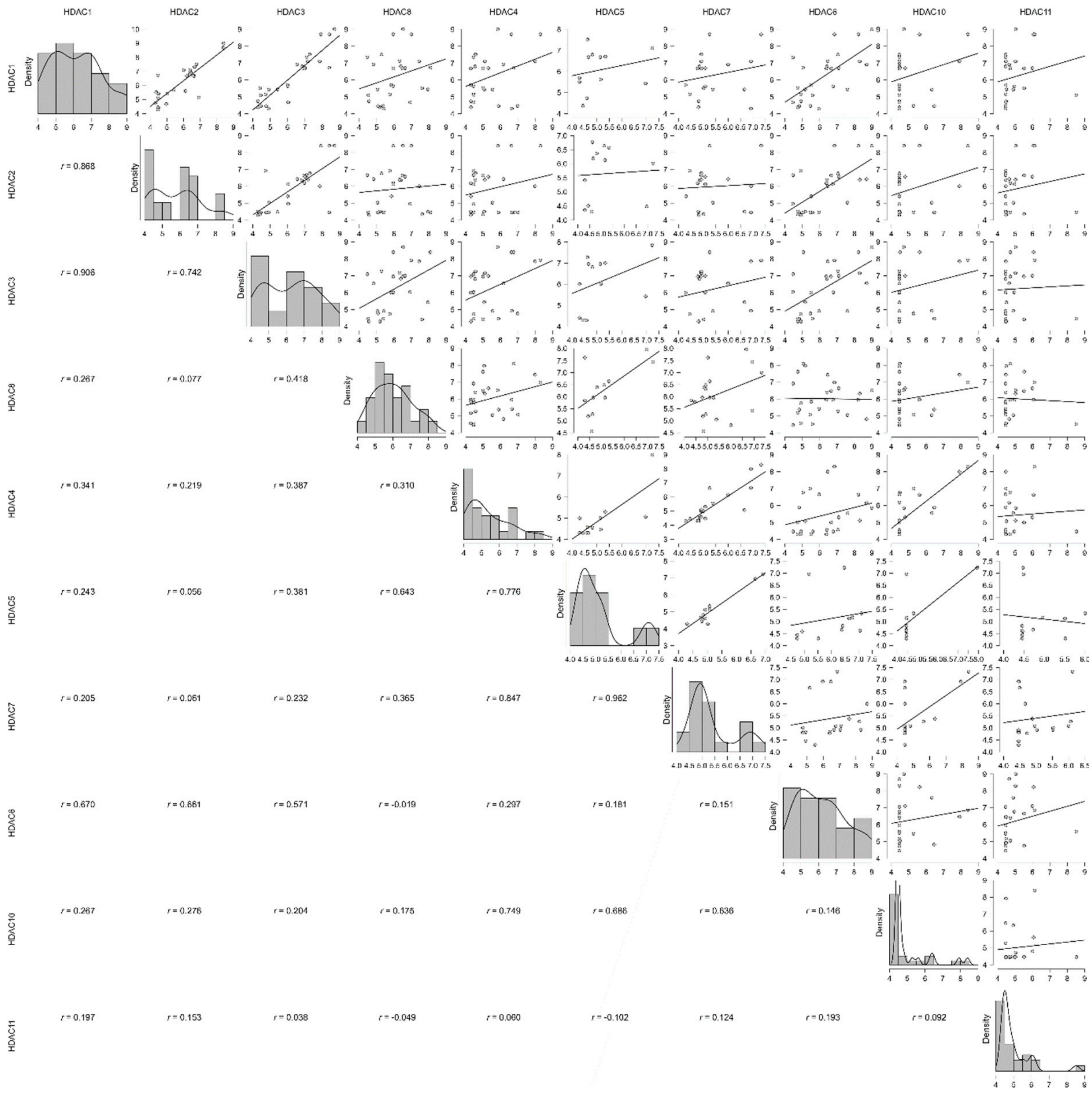

Figure S5: Correlation of pIC50 values of profile compounds for all pairs of HDACs. Each dot represents an HDACi. Correlation plots are shown above the diagonal. Corresponding Pearson's coefficients, r, are shown below the diagonal. Distribution plots of pIC50-values for every HDAC are shown on the diagonal. * p < .05, ** p < .01, *** p < .001. High correlation between the pIC50 values indicates similar binding pockets.

A)

B)

Name	Number of mutations	Mutation positions
HDAC11 4A	8	R15Q, A88V, M123L, T176L, S245A, L250R, A276E, V294I
HDAC11 7A	19	T4E, E13P, R15Q, V45I, K53G, A88V, T91R, M123L, G125A, S146A, T176L, Q217R. S245A, Q247E, L250K, A276E, G291A, R293N, V294I
HDAC11 8A	29	T4Q, H10D, E13P, R15Q, V45I, K53G, S58D, M59T, R77P, E82S, A88V, T91R, M123L, G125A, S146A, T176L, R213E, Q217R, S245A, Q247E, H249F, L250K, V253I, A276E, V279I, G291A, R293N, V294I, G321S

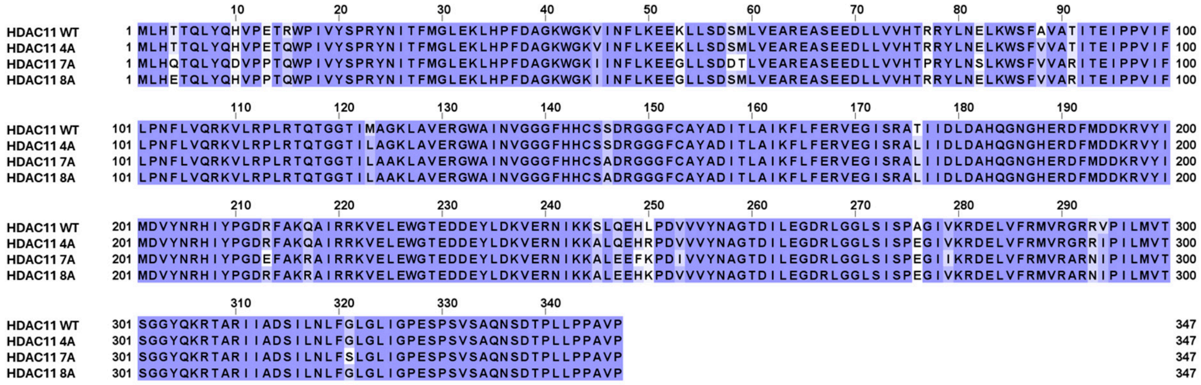

Figure S6: Mutation map of HDAC11 variants. A) Exact positions of indicated amino acid exchanges. B) Graphical representation of amino acid exchanges in a multiple sequence alignment with the wild type sequence of HDAC11. Mutated positions range between white and light blue), while unchanged poFsitions are colored blue.

Pearson's r

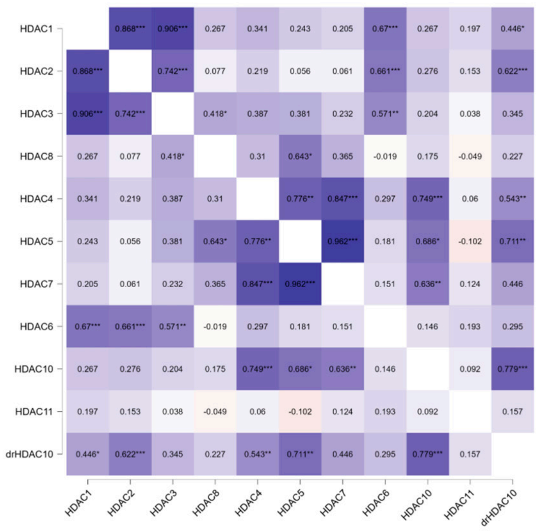

Spearman's rho

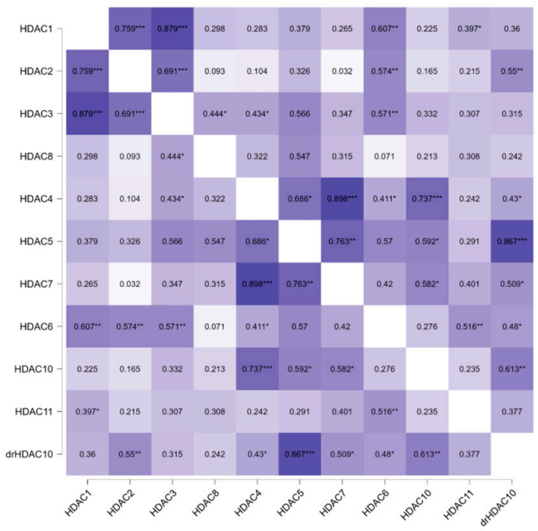

Pearson's r

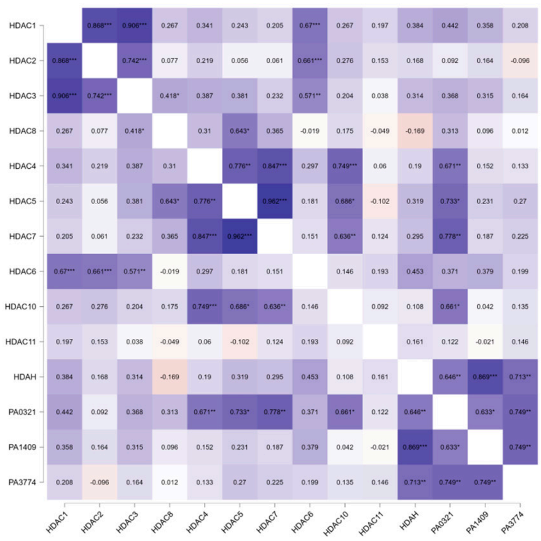

Spearman's rho

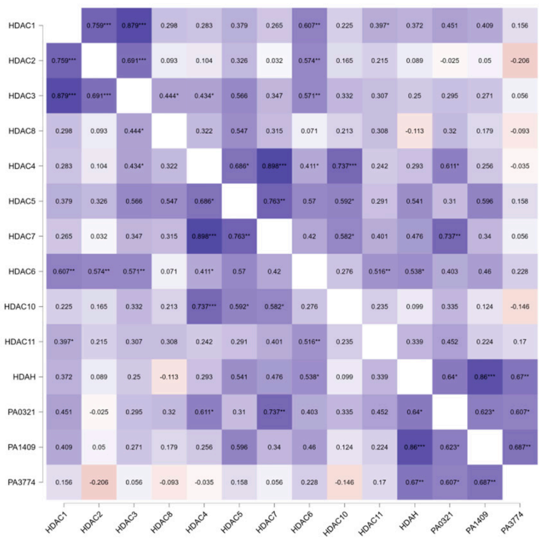

Pearson's r for Single Mutant variants of HDAC4/8:

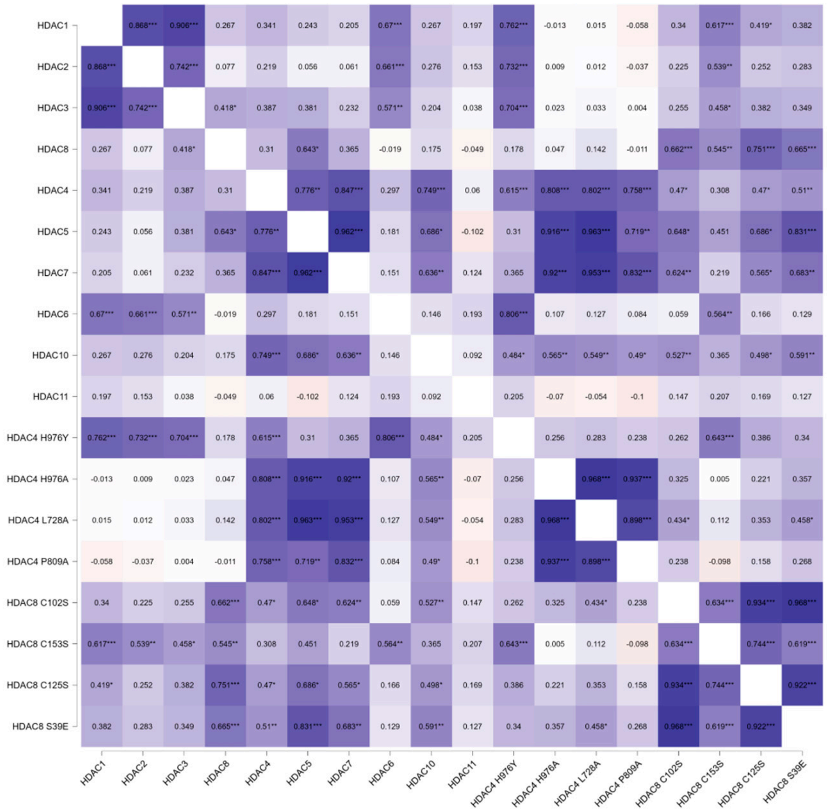

Spearman's rho for Single Mutant variants of HDAC4/8:

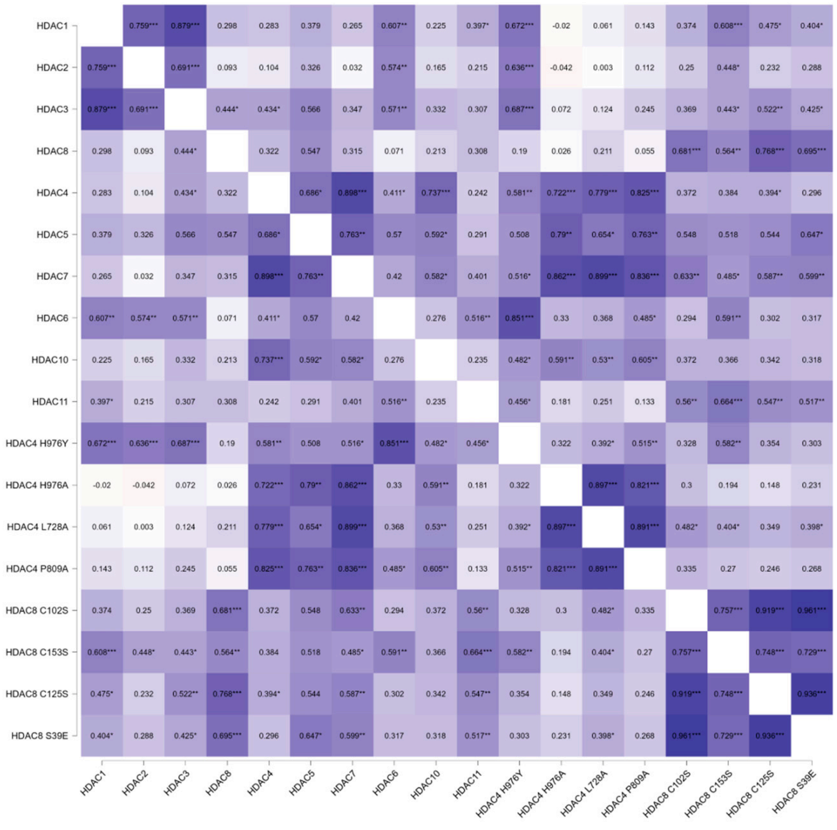

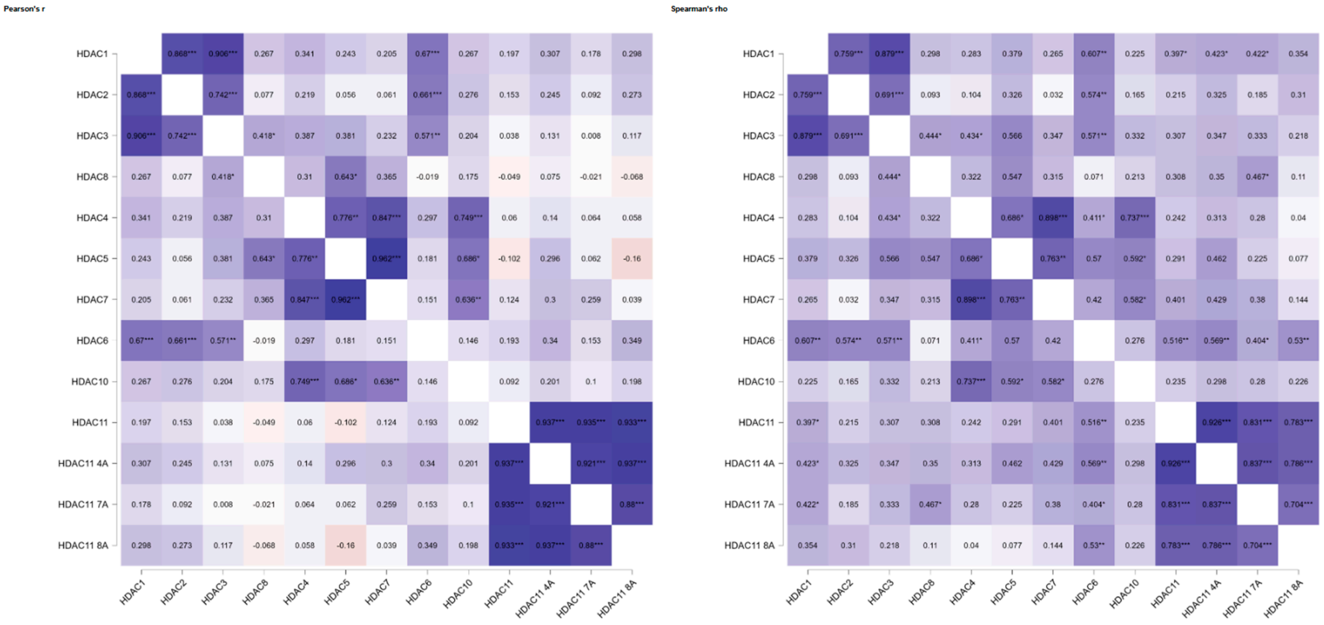

Figure S7: Comparison of heatmaps of Pearson's r and Spearman' rho correlation coefficients for chemical profiles of indicated pairs of HDAC isozymes. Blue color means high correlation, i.e. similar inhibition pattern, while red color indicates negative Pearson's coefficients. * $p < .05$, ** $p < .01$, *** $p < .001$.

Table S2: Pearson's r and Spearman's rho coefficients, and 95% confidential intervals of chemical profiles for all pairs of HDACs, HDAC variants and APAHs. * p < .05, ** p < .01, *** p < .001. The starred p-values are nominal and exploratory. A Benjamini-Hochberg correction was applied to the data. The adjusted p-values and significance for each pair are summarized in Table S4.

			Pearson				Spearman			
			r	p	Lower 95% CI	Upper 95% CI	rho	p	Lower 95% CI	Upper 95% CI
HDAC1	-	HDAC2	0.8680***	< .001	0.7241	0.9395	0.7585***	< .001	0.5256	0.8856
HDAC1	-	HDAC3	0.9059***	< .001	0.7989	0.9573	0.8793***	< .001	0.7461	0.9448
HDAC1	-	HDAC8	0.2673	.187	−0.1340	0.5932	0.2982	.139	−0.1008	0.6146
HDAC1	-	HDAC4	0.3406	.089	−0.05382	0.6431	0.2834	.161	−0.1168	0.6044
HDAC1	-	HDAC5	0.2427	.447	−0.3848	0.7168	0.3789	.224	−0.2491	0.7826
HDAC1	-	HDAC7	0.2052	.399	−0.2746	0.6032	0.2646	.274	−0.2155	0.6417
HDAC1	-	HDAC6	0.6702***	< .001	0.3820	0.8396	0.6069**	.001	0.2870	0.8050
HDAC1	-	HDAC10	0.2666	.198	−0.1436	0.5987	0.2251	.279	−0.1867	0.5696
HDAC1	-	HDAC11	0.1974	.334	−0.2057	0.5432	0.3968*	.045	0.01118	0.6797
HDAC1	-	drHDAC10	0.4456*	.023	0.07036	0.7103	0.3602	.071	−0.03156	0.6560
HDAC1	-	HDAC8 C102S	0.3402	.089	−0.05428	0.6429	0.3741	.060	−0.01547	0.6651
HDAC1	-	HDAC8 C153S	0.6168***	< .001	0.3014	0.8105	0.6079***	< .001	0.2885	0.8056
HDAC1	-	HDAC8 C125S	0.4191*	.033	0.03787	0.6938	0.4748*	.014	0.1071	0.7282
HDAC1	-	HDAC8 S39E	0.3820	.054	−0.006294	0.6702	0.4040*	.041	0.01975	0.6843
HDAC1	-	HDAC4 H976Y	0.7619***	< .001	0.5314	0.8874	0.6724***	< .001	0.3854	0.8408
HDAC1	-	HDAC4 L728A	0.01457	.944	−0.3749	0.3997	0.06075	.768	−0.3345	0.4378
HDAC1	-	HDAC4 P809A	−0.05763	.780	−0.4353	0.3372	0.1428	.486	−0.2589	0.5024
HDAC1	-	HDAC4 H976A	−0.01337	.948	−0.3987	0.3759	−0.01955	.924	−0.4038	0.3706
HDAC1	-	HDAC11 4A	0.3075	.127	−0.09067	0.6209	0.4230*	.031	0.04258	0.6962
HDAC1	-	HDAC11 7A	0.1783	.384	−0.2246	0.5291	0.4219*	.032	0.04135	0.6956
HDAC1	-	HDAC11 8A	0.2979	.139	−0.1011	0.6144	0.3543	.076	−0.03829	0.6522
HDAC1	-	HDAH	0.3835	.158	−0.1602	0.7487	0.3718	.172	−0.1736	0.7426
HDAC1	-	PA0321	0.4418	.099	−0.09107	0.7780	0.4506	.092	−0.08013	0.7823
HDAC1	-	PA1409	0.3576	.191	−0.1894	0.7352	0.4090	.130	−0.1307	0.7617
HDAC1	-	PA3774	0.2083	.456	−0.3403	0.6511	0.1561	.579	−0.3872	0.6188
HDAC2	-	HDAC3	0.7415***	< .001	0.4969	0.8770	0.6906***	< .001	0.4140	0.8505
HDAC2	-	HDAC8	0.07678	.709	−0.3201	0.4507	0.09310	.651	−0.3053	0.4637
HDAC2	-	HDAC4	0.2190	.282	−0.1839	0.5589	0.1035	.615	−0.2957	0.4720
HDAC2	-	HDAC5	0.05586	.863	−0.5352	0.6102	0.3257	.301	−0.3052	0.7580
HDAC2	-	HDAC7	0.06079	.805	−0.4046	0.5012	0.03168	.898	−0.4287	0.4790
HDAC2	-	HDAC6	0.6605***	< .001	0.3671	0.8344	0.5739**	.002	0.2399	0.7864
HDAC2	-	HDAC10	0.2761	.182	−0.1336	0.6052	0.1650	.431	−0.2462	0.5259
HDAC2	-	HDAC11	0.1533	.455	−0.2488	0.5103	0.2153	.291	−0.1877	0.5562
HDAC2	-	drHDAC10	0.6224***	< .001	0.3097	0.8136	0.5499**	.004	0.2065	0.7727
HDAC2	-	HDAC8 C102S	0.2249	.269	−0.1779	0.5632	0.2504	.217	−0.1517	0.5814
HDAC2	-	HDAC8 C153S	0.5394**	.004	0.1922	0.7666	0.4485*	.022	0.07400	0.7121
HDAC2	-	HDAC8 C125S	0.2517	.215	−0.1503	0.5823	0.2318	.255	−0.1709	0.5681
HDAC2	-	HDAC8 S39E	0.2835	.161	−0.1167	0.6044	0.2881	.154	−0.1117	0.6076
HDAC2	-	HDAC4 H976Y	0.7316***	< .001	0.4804	0.8719	0.6359***	< .001	0.3297	0.8210
HDAC2	-	HDAC4 L728A	0.01248	.952	−0.3767	0.3979	0.003181	.988	−0.3846	0.3901
HDAC2	-	HDAC4 P809A	−0.03717	.857	−0.4185	0.3553	0.1117	.587	−0.2881	0.4784
HDAC2	-	HDAC4 H976A	0.008546	.967	−0.3801	0.3946	−0.04242	.837	−0.4228	0.3507
HDAC2	-	HDAC11 4A	0.2452	.227	−0.1570	0.5777	0.3251	.105	−0.07121	0.6328
HDAC2	-	HDAC11 7A	0.09223	.654	−0.3061	0.4630	0.1847	.366	−0.2183	0.5338
HDAC2	-	HDAC11 8A	0.2733	.177	−0.1275	0.5974	0.3104	.123	−0.08745	0.6229
HDAC2	-	HDAH	0.1683	.549	−0.3764	0.6266	0.08937	.751	−0.4432	0.5753
HDAC2	-	PA0321	0.09197	.744	−0.4411	0.5770	−0.02513	.929	−0.5306	0.4935
HDAC2	-	PA1409	0.1645	.558	−0.3798	0.6241	0.05022	.859	−0.4742	0.5484
HDAC2	-	PA3774	−0.09575	.734	−0.5796	0.4380	−0.2063	.461	−0.6499	0.3421
HDAC3	-	HDAC8	0.4181*	.034	0.03672	0.6932	0.4441*	.023	0.06854	0.7094
HDAC3	-	HDAC4	0.3866	.051	-9.106×10^{-4}	0.6731	0.4341*	.027	0.05623	0.7032
HDAC3	-	HDAC5	0.3814	.221	−0.2464	0.7838	0.5659	.055	−0.01184	0.8604
HDAC3	-	HDAC7	0.2320	.339	−0.2484	0.6208	0.3474	.145	−0.1268	0.6924
HDAC3	-	HDAC6	0.5706**	.002	0.2352	0.7845	0.5710**	.002	0.2359	0.7848

HDAC3	-	HDAC10	0.2042	.327	−0.2077	0.5546	0.3324	.104	−0.07220	0.6431
HDAC3	-	HDAC11	0.03763	.855	−0.3549	0.4189	0.3070	.127	−0.09115	0.6206
HDAC3	-	drHDAC10	0.3455	.084	−0.04834	0.6463	0.3150	.117	−0.08239	0.6260
HDAC3	-	HDAC8 C102S	0.2555	.208	−0.1463	0.5850	0.3693	.063	−0.02102	0.6620
HDAC3	-	HDAC8 C153S	0.4575*	.019	0.08528	0.7177	0.4433*	.023	0.06750	0.7089
HDAC3	-	HDAC8 C125S	0.3817	.054	−0.006642	0.6700	0.5220**	.006	0.1688	0.7564
HDAC3	-	HDAC8 S39E	0.3492	.080	−0.04408	0.6488	0.4248*	.031	0.04482	0.6974
HDAC3	-	HDAC4 H976Y	0.7041***	< .001	0.4356	0.8576	0.6865***	< .001	0.4076	0.8483
HDAC3	-	HDAC4 L728A	0.03274	.874	−0.3592	0.4148	0.1243	.545	−0.2763	0.4882
HDAC3	-	HDAC4 P809A	0.003988	.985	−0.3840	0.3907	0.2452	.227	−0.1570	0.5777
HDAC3	-	HDAC4 H976A	0.02264	.913	−0.3679	0.4064	0.07240	.725	−0.3240	0.4472
HDAC3	-	HDAC11 4A	0.1307	.525	−0.2704	0.4931	0.3466	.083	−0.04705	0.6471
HDAC3	-	HDAC11 7A	0.008257	.968	−0.3803	0.3943	0.3330	.096	−0.06236	0.6381
HDAC3	-	HDAC11 8A	0.1168	.570	−0.2834	0.4823	0.2177	.285	−0.1852	0.5580
HDAC3	-	HDAH	0.3145	.254	−0.2358	0.7120	0.2504	.368	−0.3003	0.6760
HDAC3	-	PA0321	0.3677	.178	−0.1781	0.7405	0.2947	.286	−0.2562	0.7011
HDAC3	-	PA1409	0.3150	.253	−0.2352	0.7123	0.2711	.328	−0.2801	0.6878
HDAC3	-	PA3774	0.1638	.560	−0.3804	0.6237	0.05566	.844	−0.4700	0.5522
HDAC8	-	HDAC4	0.3097	.124	−0.08820	0.6224	0.3223	.108	−0.07436	0.6309
HDAC8	-	HDAC5	0.6432*	.024	0.1098	0.8890	0.5474	.065	−0.03869	0.8532
HDAC8	-	HDAC7	0.3652	.124	−0.1067	0.7028	0.3154	.188	−0.1620	0.6732
HDAC8	-	HDAC6	−0.01882	.927	−0.4032	0.3712	0.07051	.732	−0.3257	0.4457
HDAC8	-	HDAC10	0.1747	.404	−0.2368	0.5330	0.2128	.307	−0.1991	0.5607
HDAC8	-	HDAC11	−0.04918	.811	−0.4284	0.3447	0.3082	.126	−0.08986	0.6214
HDAC8	-	drHDAC10	0.2274	.264	−0.1754	0.5650	0.2420	.234	−0.1604	0.5754
HDAC8	-	HDAC8 C102S	0.6625***	< .001	0.3701	0.8354	0.6808***	< .001	0.3986	0.8453
HDAC8	-	HDAC8 C153S	0.5454**	.004	0.2004	0.7701	0.5638**	.003	0.2258	0.7807
HDAC8	-	HDAC8 C125S	0.7509***	< .001	0.5126	0.8818	0.7676***	< .001	0.5412	0.8903
HDAC8	-	HDAC8 S39E	0.6654***	< .001	0.3746	0.8370	0.6953***	< .001	0.4214	0.8529
HDAC8	-	HDAC4 H976Y	0.1776	.385	−0.2253	0.5286	0.1900	.353	−0.2130	0.5378
HDAC8	-	HDAC4 L728A	0.1417	.490	−0.2599	0.5015	0.2113	.300	−0.1917	0.5534
HDAC8	-	HDAC4 P809A	−0.01131	.956	−0.3969	0.3777	0.05548	.788	−0.3392	0.4335
HDAC8	-	HDAC4 H976A	0.04671	.821	−0.3469	0.4263	0.02571	.901	−0.3653	0.4090
HDAC8	-	HDAC11 4A	0.07518	.715	−0.3215	0.4494	0.3500	.080	−0.04317	0.6493
HDAC8	-	HDAC11 7A	−0.02084	.920	−0.4049	0.3695	0.4668*	.016	0.09697	0.7234
HDAC8	-	HDAC11 8A	−0.06828	.740	−0.4439	0.3277	0.1104	.591	−0.2893	0.4773
HDAC8	-	HDAH	−0.1687	.548	−0.6268	0.3760	−0.1126	.689	−0.5908	0.4241
HDAC8	-	PA0321	0.3134	.255	−0.2369	0.7114	0.3196	.246	−0.2304	0.7148
HDAC8	-	PA1409	0.09594	.734	−0.4378	0.5797	0.1794	.522	−0.3666	0.6334
HDAC8	-	PA3774	0.01181	.967	−0.5035	0.5209	−0.09327	.741	−0.5779	0.4400
HDAC4	-	HDAC5	0.7762**	.003	0.3647	0.9340	0.6858*	.014	0.1846	0.9039
HDAC4	-	HDAC7	0.8471***	< .001	0.6387	0.9398	0.8979***	< .001	0.7492	0.9604
HDAC4	-	HDAC6	0.2968	.141	−0.1024	0.6136	0.4111*	.037	0.02826	0.6888
HDAC4	-	HDAC10	0.7494***	< .001	0.5033	0.8831	0.7366***	< .001	0.4816	0.8766
HDAC4	-	HDAC11	0.06037	.770	−0.3348	0.4375	0.2415	.235	−0.1609	0.5751
HDAC4	-	drHDAC10	0.5432**	.004	0.1974	0.7688	0.4303*	.028	0.05156	0.7008
HDAC4	-	HDAC8 C102S	0.4705*	.015	0.1017	0.7256	0.3721	.061	−0.01780	0.6638
HDAC4	-	HDAC8 C153S	0.3080	.126	−0.09009	0.6212	0.3843	.053	−0.003586	0.6717
HDAC4	-	HDAC8 C125S	0.4704*	.015	0.1016	0.7256	0.3937*	.047	0.007436	0.6777
HDAC4	-	HDAC8 S39E	0.5095**	.008	0.1522	0.7490	0.2964	.141	−0.1027	0.6133
HDAC4	-	HDAC4 H976Y	0.6155***	< .001	0.2995	0.8098	0.5806**	.002	0.2493	0.7902
HDAC4	-	HDAC4 L728A	0.8018***	< .001	0.6012	0.9074	0.7790***	< .001	0.5608	0.8960
HDAC4	-	HDAC4 P809A	0.7577***	< .001	0.5242	0.8852	0.8245***	< .001	0.6423	0.9185
HDAC4	-	HDAC4 H976A	0.8084***	< .001	0.6131	0.9106	0.7219***	< .001	0.4645	0.8669
HDAC4	-	HDAC11 4A	0.1405	.494	−0.2611	0.5006	0.3131	.119	−0.08448	0.6247
HDAC4	-	HDAC11 7A	0.06396	.756	−0.3316	0.4404	0.2803	.166	−0.1201	0.6022
HDAC4	-	HDAC11 8A	0.05774	.779	−0.3372	0.4354	0.04046	.844	−0.3524	0.4212
HDAC4	-	HDAH	0.1899	.498	−0.3571	0.6399	0.2932	.289	−0.2577	0.7003

HDAC4	-	PA0321	0.6707**	.006	0.2414	0.8805	0.6109*	.016	0.1436	0.8555
HDAC4	-	PA1409	0.1522	.588	−0.3905	0.6164	0.2561	.357	−0.2948	0.6793
HDAC4	-	PA3774	0.1334	.636	−0.4067	0.6044	−0.03452	.903	−0.5373	0.4863
HDAC5	-	HDAC7	0.9618***	< .001	0.8659	0.9895	0.7627**	.004	0.3359	0.9297
HDAC5	-	HDAC6	0.1811	.573	−0.4384	0.6839	0.5702	.053	−0.005539	0.8620
HDAC5	-	HDAC10	0.6858*	.014	0.1845	0.9039	0.5923*	.042	0.02795	0.8704
HDAC5	-	HDAC11	−0.1017	.753	−0.6383	0.5015	0.2906	.360	−0.3400	0.7409
HDAC5	-	drHDAC10	0.7112**	.010	0.2319	0.9126	0.8671***	< .001	0.5836	0.9622
HDAC5	-	HDAC8 C102S	0.6480*	.023	0.1179	0.8907	0.5477	.065	−0.03815	0.8534
HDAC5	-	HDAC8 C153S	0.4513	.141	−0.1655	0.8143	0.5184	.084	−0.07902	0.8418
HDAC5	-	HDAC8 C125S	0.6858*	.014	0.1845	0.9039	0.5437	.068	−0.04384	0.8518
HDAC5	-	HDAC8 S39E	0.8313***	< .001	0.4923	0.9513	0.6471*	.023	0.1165	0.8904
HDAC5	-	HDAC4 H976Y	0.3099	.327	−0.3211	0.7504	0.5079	.092	−0.09318	0.8376
HDAC5	-	HDAC4 L728A	0.9632***	< .001	0.8703	0.9899	0.6537*	.021	0.1278	0.8927
HDAC5	-	HDAC4 P809A	0.7193**	.008	0.2476	0.9153	0.7632**	.004	0.3368	0.9298
HDAC5	-	HDAC4 H976A	0.9158***	< .001	0.7207	0.9765	0.7896**	.002	0.3945	0.9383
HDAC5	-	HDAC11 4A	0.2965	.349	−0.3343	0.7438	0.4617	.131	−0.1526	0.8187
HDAC5	-	HDAC11 7A	0.06161	.849	−0.5311	0.6138	0.2252	.482	−0.4005	0.7076
HDAC5	-	HDAC11 8A	−0.1603	.619	−0.6723	0.4555	0.07706	.812	−0.5198	0.6234
HDAC5	-	HDAH	0.3195	.368	−0.3883	0.7902	0.5410	.106	−0.1344	0.8732
HDAC5	-	PA0321	0.7332*	.016	0.1923	0.9324	0.3100	.383	−0.3971	0.7862
HDAC5	-	PA1409	0.2308	.521	−0.4666	0.7513	0.5957	.069	−0.05421	0.8911
HDAC5	-	PA3774	0.2704	.450	−0.4330	0.7691	0.1581	.663	−0.5237	0.7164
HDAC7	-	HDAC6	0.1511	.537	−0.3255	0.5664	0.4199	.073	−0.04240	0.7341
HDAC7	-	HDAC10	0.6362**	.005	0.2408	0.8504	0.5817*	.011	0.1576	0.8246
HDAC7	-	HDAC11	0.1238	.614	−0.3501	0.5472	0.4007	.089	−0.06546	0.7232
HDAC7	-	drHDAC10	0.4458	.056	−0.01060	0.7484	0.5086*	.026	0.07074	0.7821
HDAC7	-	HDAC8 C102S	0.6238**	.004	0.2367	0.8400	0.6334**	.004	0.2515	0.8446
HDAC7	-	HDAC8 C153S	0.2186	.369	−0.2616	0.6120	0.4848*	.035	0.03926	0.7696
HDAC7	-	HDAC8 C125S	0.5654*	.012	0.1496	0.8113	0.5874**	.008	0.1817	0.8223
HDAC7	-	HDAC8 S39E	0.6832**	.001	0.3320	0.8680	0.5992**	.007	0.1992	0.8280
HDAC7	-	HDAC4 H976Y	0.3647	.125	−0.1072	0.7026	0.5163*	.024	0.08108	0.7861
HDAC7	-	HDAC4 L728A	0.9527***	< .001	0.8787	0.9820	0.8987***	< .001	0.7511	0.9607
HDAC7	-	HDAC4 P809A	0.8321***	< .001	0.6076	0.9335	0.8364***	< .001	0.6164	0.9353
HDAC7	-	HDAC4 H976A	0.9200***	< .001	0.8003	0.9692	0.8622***	< .001	0.6706	0.9460
HDAC7	-	HDAC11 4A	0.3005	.211	−0.1780	0.6641	0.4294	.067	−0.03081	0.7394
HDAC7	-	HDAC11 7A	0.2586	.285	−0.2217	0.6379	0.3797	.109	−0.09005	0.7112
HDAC7	-	HDAC11 8A	0.03883	.875	−0.4228	0.4845	0.1444	.555	−0.3315	0.5618
HDAC7	-	HDAH	0.2946	.353	−0.3361	0.7429	0.4764	.117	−0.1342	0.8248
HDAC7	-	PA0321	0.7778**	.003	0.3684	0.9345	0.7368**	.006	0.2823	0.9212
HDAC7	-	PA1409	0.1866	.561	−0.4337	0.6869	0.3404	.279	−0.2902	0.7649
HDAC7	-	PA3774	0.2254	.481	−0.4003	0.7077	0.05614	.862	−0.5350	0.6104
HDAC6	-	HDAC10	0.1455	.488	−0.2648	0.5112	0.2763	.181	−0.1334	0.6053
HDAC6	-	HDAC11	0.1933	.344	−0.2098	0.5402	0.5156**	.007	0.1603	0.7526
HDAC6	-	drHDAC10	0.2949	.144	−0.1044	0.6123	0.4804*	.013	0.1143	0.7316
HDAC6	-	HDAC8 C102S	0.05929	.774	−0.3358	0.4366	0.2942	.145	−0.1052	0.6118
HDAC6	-	HDAC8 C153S	0.5642**	.003	0.2263	0.7809	0.5912**	.001	0.2643	0.7962
HDAC6	-	HDAC8 C125S	0.1665	.416	−0.2361	0.5203	0.3021	.134	−0.09655	0.6172
HDAC6	-	HDAC8 S39E	0.1295	.528	−0.2715	0.4922	0.3170	.115	−0.08024	0.6273
HDAC6	-	HDAC4 H976Y	0.8057***	< .001	0.6082	0.9093	0.8509***	< .001	0.6915	0.9313
HDAC6	-	HDAC4 L728A	0.1272	.536	−0.2736	0.4904	0.3676	.065	−0.02300	0.6609
HDAC6	-	HDAC4 P809A	0.08429	.682	−0.3133	0.4567	0.4851*	.012	0.1204	0.7345
HDAC6	-	HDAC4 H976A	0.1074	.601	−0.2921	0.4750	0.3297	.100	−0.06614	0.6358
HDAC6	-	HDAC11 4A	0.3398	.089	−0.05473	0.6426	0.5694**	.002	0.2336	0.7839
HDAC6	-	HDAC11 7A	0.1528	.456	−0.2493	0.5100	0.4035*	.041	0.01915	0.6840
HDAC6	-	HDAC11 8A	0.3488	.081	−0.04458	0.6485	0.5296**	.005	0.1789	0.7608

HDAC6	-	HDAH	0.4533	.090	−0.07685	0.7836	0.5385*	.038	0.03618	0.8236
HDAC6	-	PA0321	0.3711	.173	−0.1743	0.7423	0.4025	.137	−0.1383	0.7584
HDAC6	-	PA1409	0.3795	.163	−0.1648	0.7466	0.4596	.085	−0.06887	0.7867
HDAC6	-	PA3774	0.1991	.477	−0.3487	0.6455	0.2280	.414	−0.3218	0.6628
HDAC10	-	HDAC11	0.09223	.661	−0.3144	0.4702	0.2354	.257	−0.1761	0.5769
HDAC10	-	drHDAC10	0.7794***	< .001	0.5552	0.8980	0.6130**	.001	0.2875	0.8116
HDAC10	-	HDAC8 C102S	0.5270**	.007	0.1666	0.7632	0.3718	.067	−0.02736	0.6687
HDAC10	-	HDAC8 C153S	0.3648	.073	−0.03548	0.6642	0.3662	.072	−0.03380	0.6651
HDAC10	-	HDAC8 C125S	0.4980*	.011	0.1281	0.7463	0.3419	.094	−0.06152	0.6493
HDAC10	-	HDAC8 S39E	0.5911**	.002	0.2556	0.7995	0.3184	.121	−0.08777	0.6338
HDAC10	-	HDAC4 H976Y	0.4845*	.014	0.1105	0.7383	0.4815*	.015	0.1067	0.7365
HDAC10	-	HDAC4 L728A	0.5487**	.005	0.1961	0.7757	0.5298**	.006	0.1703	0.7648
HDAC10	-	HDAC4 P809A	0.4901*	.013	0.1177	0.7416	0.6046**	.001	0.2753	0.8070
HDAC10	-	HDAC4 H976A	0.5655**	.003	0.2193	0.7852	0.5908**	.002	0.2553	0.7993
HDAC10	-	HDAC11 4A	0.2008	.336	−0.2111	0.5521	0.2979	.148	−0.1102	0.6200
HDAC10	-	HDAC11 7A	0.1003	.633	−0.3070	0.4766	0.2802	.175	−0.1292	0.6080
HDAC10	-	HDAC11 8A	0.1978	.343	−0.2141	0.5499	0.2256	.278	−0.1861	0.5699
HDAC10	-	HDAH	0.1078	.714	−0.4484	0.6038	0.09911	.736	−0.4554	0.5982
HDAC10	-	PA0321	0.6611*	.010	0.2011	0.8822	0.3352	.241	−0.2376	0.7351
HDAC10	-	PA1409	0.04167	.888	−0.5000	0.5599	0.1240	.673	−0.4352	0.6142
HDAC10	-	PA3774	0.1351	.645	−0.4260	0.6212	−0.1461	.618	−0.6280	0.4168
HDAC11	-	drHDAC10	0.1573	.443	−0.2450	0.5134	0.3773	.057	−0.01175	0.6672
HDAC11	-	HDAC8 C102S	0.1474	.472	−0.2545	0.5059	0.5605**	.003	0.2211	0.7788
HDAC11	-	HDAC8 C153S	0.2072	.310	−0.1958	0.5504	0.6641***	< .001	0.3726	0.8363
HDAC11	-	HDAC8 C125S	0.1686	.410	−0.2340	0.5219	0.5465**	.004	0.2019	0.7707
HDAC11	-	HDAC8 S39E	0.1268	.537	−0.2740	0.4901	0.5173**	.007	0.1625	0.7536
HDAC11	-	HDAC4 H976Y	0.2045	.316	−0.1986	0.5484	0.4562*	.019	0.08359	0.7169
HDAC11	-	HDAC4 L728A	−0.05395	.794	−0.4323	0.3405	0.2513	.216	−0.1507	0.5820
HDAC11	-	HDAC4 P809A	−0.1005	.625	−0.4695	0.2985	0.1326	.519	−0.2686	0.4945
HDAC11	-	HDAC4 H976A	−0.06973	.735	−0.4451	0.3264	0.1809	.377	−0.2220	0.5310
HDAC11	-	HDAC11 4A	0.9371***	< .001	0.8631	0.9717	0.9263***	< .001	0.8406	0.9668
HDAC11	-	HDAC11 7A	0.9345***	< .001	0.8577	0.9706	0.8305***	< .001	0.6533	0.9214
HDAC11	-	HDAC11 8A	0.9330***	< .001	0.8544	0.9698	0.7831***	< .001	0.5680	0.8980
HDAC11	-	HDAH	0.1608	.567	−0.3830	0.6219	0.3393	.216	−0.2093	0.7255
HDAC11	-	PA0321	0.1218	.666	−0.4165	0.5968	0.4520	.091	−0.07839	0.7830
HDAC11	-	PA1409	−0.02106	.941	−0.5276	0.4966	0.2240	.422	−0.3257	0.6605
HDAC11	-	PA3774	0.1457	.604	−0.3961	0.6123	0.1703	.544	−0.3747	0.6278
drHDAC10	-	HDAC8 C102S	0.5460**	.004	0.2012	0.7704	0.5516**	.003	0.2089	0.7737
drHDAC10	-	HDAC8 C153S	0.5057**	.008	0.1471	0.7468	0.6109***	< .001	0.2929	0.8072
drHDAC10	-	HDAC8 C125S	0.5038**	.009	0.1446	0.7456	0.4143*	.035	0.03210	0.6908
drHDAC10	-	HDAC8 S39E	0.5844**	.002	0.2547	0.7924	0.4868*	.012	0.1225	0.7354
drHDAC10	-	HDAC4 H976Y	0.6036**	.001	0.2823	0.8032	0.5789**	.002	0.2469	0.7893
drHDAC10	-	HDAC4 L728A	0.3751	.059	−0.01438	0.6657	0.4632*	.017	0.09248	0.7212
drHDAC10	-	HDAC4 P809A	0.2869	.155	−0.1130	0.6068	0.4222*	.032	0.04160	0.6957
drHDAC10	-	HDAC4 H976A	0.3482	.081	−0.04525	0.6481	0.4940*	.010	0.1318	0.7398
drHDAC10	-	HDAC11 4A	0.2397	.238	−0.1628	0.5738	0.4858*	.012	0.1213	0.7349
drHDAC10	-	HDAC11 7A	0.1224	.551	−0.2781	0.4867	0.3382	.091	−0.05654	0.6415
drHDAC10	-	HDAC11 8A	0.1986	.331	−0.2045	0.5441	0.2320	.254	−0.1707	0.5683
drHDAC10	-	HDAH	−0.1673	.551	−0.6259	0.3773	0.01209	.966	−0.5033	0.5211
drHDAC10	-	PA0321	0.1942	.488	−0.3532	0.6425	0.1902	.497	−0.3568	0.6401
drHDAC10	-	PA1409	−0.1861	.507	−0.6376	0.3606	0.02022	.943	−0.4972	0.5270
drHDAC10	-	PA3774	−0.2582	.353	−0.6805	0.2928	−0.1698	.545	−0.6275	0.3751
HDAC8 C102S	-	HDAC8 C153S	0.6335***	< .001	0.3263	0.8197	0.7572***	< .001	0.5233	0.8850
HDAC8 C102S	-	HDAC8 C125S	0.9339***	< .001	0.8562	0.9702	0.9188***	< .001	0.8250	0.9633
HDAC8 C102S	-	HDAC8 S39E	0.9685***	< .001	0.9300	0.9859	0.9614***	< .001	0.9146	0.9828
HDAC8 C102S	-	HDAC4 H976Y	0.2624	.195	−0.1390	0.5898	0.3281	.102	−0.06785	0.6348

HDAC8 C102S	-	HDAC4 L728A	0.4344*	.027	0.05660	0.7034	0.4822*	.013	0.1166	0.7327
HDAC8 C102S	-	HDAC4 P809A	0.2383	.241	−0.1643	0.5727	0.3354	.094	−0.05968	0.6397
HDAC8 C102S	-	HDAC4 H976A	0.3252	.105	−0.07110	0.6328	0.2999	.137	−0.09898	0.6157
HDAC8 C102S	-	HDAC11 4A	0.2850	.158	−0.1150	0.6055	0.5995**	.001	0.2764	0.8009
HDAC8 C102S	-	HDAC11 7A	0.2300	.258	−0.1727	0.5669	0.6816***	< .001	0.3998	0.8457
HDAC8 C102S	-	HDAC11 8A	0.1428	.486	−0.2588	0.5024	0.4170*	.034	0.03536	0.6925
HDAC8 C102S	-	HDAH	−0.1880	.502	−0.6387	0.3588	−0.05617	.842	−0.5525	0.4696
HDAC8 C102S	-	PA0321	0.5146*	.050	0.003155	0.8126	0.5243*	.045	0.01651	0.8171
HDAC8 C102S	-	PA1409	−0.08952	.751	−0.5754	0.4431	0.05832	.836	−0.4679	0.5540
HDAC8 C102S	-	PA3774	−0.02883	.919	−0.5332	0.4907	−0.1225	.664	−0.5973	0.4159
HDAC8 C153S	-	HDAC8 C125S	0.7443***	< .001	0.5015	0.8784	0.7475***	< .001	0.5070	0.8801
HDAC8 C153S	-	HDAC8 S39E	0.6189***	< .001	0.3046	0.8117	0.7291***	< .001	0.4763	0.8706
HDAC8 C153S	-	HDAC4 H976Y	0.6430***	< .001	0.3405	0.8249	0.5817**	.002	0.2508	0.7908
HDAC8 C153S	-	HDAC4 L728A	0.1123	.585	−0.2876	0.4788	0.4044*	.040	0.02027	0.6846
HDAC8 C153S	-	HDAC4 P809A	−0.09811	.634	−0.4677	0.3007	0.2703	.182	−0.1307	0.5953
HDAC8 C153S	-	HDAC4 H976A	0.004591	.982	−0.3834	0.3912	0.1936	.343	−0.2094	0.5404
HDAC8 C153S	-	HDAC11 4A	0.3828	.054	−0.005401	0.6707	0.7378***	< .001	0.4906	0.8750
HDAC8 C153S	-	HDAC11 7A	0.1607	.433	−0.2417	0.5159	0.6797***	< .001	0.3969	0.8447
HDAC8 C153S	-	HDAC11 8A	0.3428	.086	−0.05135	0.6446	0.6038**	.001	0.2825	0.8033
HDAC8 C153S	-	HDAH	0.3279	.233	−0.2216	0.7193	0.2964	.283	−0.2545	0.7021
HDAC8 C153S	-	PA0321	0.5747*	.025	0.08849	0.8397	0.6422**	.010	0.1936	0.8687
HDAC8 C153S	-	PA1409	0.3759	.167	−0.1689	0.7447	0.3871	.154	−0.1561	0.7505
HDAC8 C153S	-	PA3774	0.2261	.418	−0.3237	0.6617	0.04660	.869	−0.4771	0.5458
HDAC8 C125S	-	HDAC8 S39E	0.9223***	< .001	0.8323	0.9649	0.9364***	< .001	0.8616	0.9714
HDAC8 C125S	-	HDAC4 H976Y	0.3858	.052	−0.001814	0.6726	0.3541	.076	−0.03853	0.6520
HDAC8 C125S	-	HDAC4 L728A	0.3528	.077	−0.03997	0.6512	0.3491	.080	−0.04424	0.6487
HDAC8 C125S	-	HDAC4 P809A	0.1575	.442	−0.2447	0.5136	0.2462	.225	−0.1560	0.5784
HDAC8 C125S	-	HDAC4 H976A	0.2213	.277	−0.1816	0.5606	0.1483	.470	−0.2536	0.5066
HDAC8 C125S	-	HDAC11 4A	0.3396	.090	−0.05503	0.6424	0.5918**	.001	0.2653	0.7966
HDAC8 C125S	-	HDAC11 7A	0.2073	.310	−0.1958	0.5504	0.6359***	< .001	0.3299	0.8210
HDAC8 C125S	-	HDAC11 8A	0.2177	.285	−0.1852	0.5580	0.3858	.052	−0.001790	0.6727
HDAC8 C125S	-	HDAH	−0.03922	.890	−0.5406	0.4827	−0.005533	.984	−0.5163	0.5082
HDAC8 C125S	-	PA0321	0.5584*	.030	0.06467	0.8325	0.5141*	.050	0.002477	0.8124
HDAC8 C125S	-	PA1409	0.003260	.991	−0.5099	0.5147	0.07219	.798	−0.4570	0.5636
HDAC8 C125S	-	PA3774	0.04949	.861	−0.4748	0.5479	−0.07774	.783	−0.5674	0.4525
HDAC8 S39E	-	HDAC4 H976Y	0.3399	.089	−0.05470	0.6426	0.3033	.132	−0.09521	0.6181
HDAC8 S39E	-	HDAC4 L728A	0.4585*	.018	0.08647	0.7183	0.3978*	.044	0.01240	0.6803
HDAC8 S39E	-	HDAC4 P809A	0.2678	.186	−0.1333	0.5936	0.2684	.185	−0.1327	0.5940
HDAC8 S39E	-	HDAC4 H976A	0.3569	.073	−0.03534	0.6539	0.2313	.256	−0.1714	0.5678
HDAC8 S39E	-	HDAC11 4A	0.2850	.158	−0.1150	0.6055	0.5825**	.002	0.2520	0.7913
HDAC8 S39E	-	HDAC11 7A	0.1968	.335	−0.2063	0.5428	0.6013**	.001	0.2789	0.8019
HDAC8 S39E	-	HDAC11 8A	0.1425	.487	−0.2591	0.5021	0.4121*	.036	0.02946	0.6894
HDAC8 S39E	-	HDAH	−0.09903	.725	−0.5818	0.4353	−0.05348	.850	−0.5507	0.4717
HDAC8 S39E	-	PA0321	0.5392*	.038	0.03727	0.8239	0.4733	.075	−0.05139	0.7933
HDAC8 S39E	-	PA1409	−0.04052	.886	−0.5415	0.4817	0.08699	.758	−0.4451	0.5737
HDAC8 S39E	-	PA3774	−0.02011	.943	−0.5269	0.4973	−0.07033	.803	−0.5623	0.4584
HDAC4 H976Y	-	HDAC4 L728A	0.2826	.162	−0.1177	0.6038	0.3916*	.048	0.005052	0.6764
HDAC4 H976Y	-	HDAC4 P809A	0.2376	.242	−0.1649	0.5723	0.5150**	.007	0.1594	0.7523
HDAC4 H976Y	-	HDAC4 H976A	0.2562	.207	−0.1456	0.5854	0.3216	.109	−0.07510	0.6304
HDAC4 H976Y	-	HDAC11 4A	0.3138	.119	−0.08376	0.6251	0.4462*	.022	0.07118	0.7107
HDAC4 H976Y	-	HDAC11 7A	0.09344	.650	−0.3050	0.4640	0.3537	.076	−0.03900	0.6518
HDAC4 H976Y	-	HDAC11 8A	0.3183	.113	−0.07877	0.6282	0.3972*	.045	0.01163	0.6799
HDAC4 H976Y	-	HDAH	0.4419	.099	−0.09098	0.7780	0.3821	.161	−0.1618	0.7480
HDAC4 H976Y	-	PA0321	0.4411	.100	−0.09188	0.7777	0.3247	.238	−0.2250	0.7176
HDAC4 H976Y	-	PA1409	0.3678	.177	−0.1780	0.7406	0.3871	.154	−0.1561	0.7505
HDAC4 H976Y	-	PA3774	0.1470	.601	−0.3950	0.6131	0.06452	.819	−0.4630	0.5583

HDAC4 L728A	-	HDAC4 P809A	0.8981***	< .001	0.7833	0.9537	0.8910***	< .001	0.7692	0.9504
HDAC4 L728A	-	HDAC4 H976A	0.9680***	< .001	0.9291	0.9858	0.8973***	< .001	0.7815	0.9533
HDAC4 L728A	-	HDAC11 4A	0.03901	.850	−0.3537	0.4200	0.3103	.123	−0.08762	0.6228
HDAC4 L728A	-	HDAC11 7A	−0.02445	.906	−0.4079	0.3664	0.2799	.166	−0.1205	0.6020
HDAC4 L728A	-	HDAC11 8A	−0.07911	.701	−0.4526	0.3180	0.06679	.746	−0.3291	0.4427
HDAC4 L728A	-	HDAH	0.1961	.484	−0.3515	0.6437	0.3331	.225	−0.2160	0.7222
HDAC4 L728A	-	PA0321	0.6684**	.006	0.2374	0.8795	0.6936**	.004	0.2813	0.8897
HDAC4 L728A	-	PA1409	0.1496	.595	−0.3928	0.6148	0.4063	.133	−0.1339	0.7603
HDAC4 L728A	-	PA3774	0.1690	.547	−0.3758	0.6270	0.1575	.575	−0.3859	0.6197
HDAC4 P809A	-	HDAC4 H976A	0.9368***	< .001	0.8624	0.9716	0.8205***	< .001	0.6350	0.9166
HDAC4 P809A	-	HDAC11 4A	−0.06135	.766	−0.4383	0.3339	0.2268	.265	−0.1760	0.5646
HDAC4 P809A	-	HDAC11 7A	−0.04562	.825	−0.4255	0.3479	0.1843	.367	−0.2186	0.5336
HDAC4 P809A	-	HDAC11 8A	−0.1138	.580	−0.4800	0.2862	0.02002	.923	−0.3702	0.4042
HDAC4 P809A	-	HDAH	0.1129	.689	−0.4239	0.5910	0.1283	.649	−0.4109	0.6011
HDAC4 P809A	-	PA0321	0.6534**	.008	0.2121	0.8733	0.3775	.165	−0.1670	0.7456
HDAC4 P809A	-	PA1409	0.05878	.835	−0.4676	0.5544	0.1610	.567	−0.3828	0.6220
HDAC4 P809A	-	PA3774	0.1736	.536	−0.3717	0.6298	−0.07820	.782	−0.5677	0.4522
HDAC4 H976A	-	HDAC11 4A	−0.01243	.952	−0.3979	0.3767	0.2163	.288	−0.1866	0.5570
HDAC4 H976A	-	HDAC11 7A	−0.03765	.855	−0.4189	0.3549	0.1549	.450	−0.2473	0.5116
HDAC4 H976A	-	HDAC11 8A	−0.1044	.612	−0.4726	0.2949	−0.01197	.954	−0.3975	0.3771
HDAC4 H976A	-	HDAH	0.2515	.366	−0.2993	0.6766	0.4630	.082	−0.06452	0.7883
HDAC4 H976A	-	PA0321	0.7208**	.002	0.3306	0.9006	0.6145*	.015	0.1492	0.8570
HDAC4 H976A	-	PA1409	0.2335	.402	−0.3167	0.6661	0.4534	.090	−0.07670	0.7836
HDAC4 H976A	-	PA3774	0.2557	.358	−0.2952	0.6790	0.2985	.280	−0.2524	0.7032
HDAC11 4A	-	HDAC11 7A	0.9214***	< .001	0.8305	0.9645	0.8368***	< .001	0.6650	0.9245
HDAC11 4A	-	HDAC11 8A	0.9371***	< .001	0.8631	0.9717	0.7855***	< .001	0.5723	0.8993
HDAC11 4A	-	HDAH	0.1952	.486	−0.3523	0.6431	0.2800	.312	−0.2711	0.6929
HDAC11 4A	-	PA0321	0.2001	.475	−0.3478	0.6461	0.3562	.193	−0.1909	0.7344
HDAC11 4A	-	PA1409	0.01914	.946	−0.4980	0.5262	0.1059	.707	−0.4297	0.5863
HDAC11 4A	-	PA3774	0.1800	.521	−0.3660	0.6338	0.07665	.786	−0.4534	0.5667
HDAC11 7A	-	HDAC11 8A	0.8801***	< .001	0.7477	0.9452	0.7043***	< .001	0.4358	0.8576
HDAC11 7A	-	HDAH	0.03444	.903	−0.4864	0.5372	0.06776	.810	−0.4605	0.5606
HDAC11 7A	-	PA0321	0.1630	.562	−0.3811	0.6232	0.4992	.058	−0.01760	0.8055
HDAC11 7A	-	PA1409	−0.1434	.610	−0.6108	0.3981	−0.02267	.936	−0.5288	0.4953
HDAC11 7A	-	PA3774	0.1115	.692	−0.4250	0.5901	0.07934	.779	−0.4513	0.5685
HDAC11 8A	-	HDAH	0.2626	.344	−0.2884	0.6830	0.4486	.093	−0.08264	0.7813
HDAC11 8A	-	PA0321	0.1718	.540	−0.3733	0.6287	0.3878	.153	−0.1553	0.7509
HDAC11 8A	-	PA1409	0.03400	.904	−0.4867	0.5369	0.2368	.396	−0.3135	0.6680
HDAC11 8A	-	PA3774	0.1675	.551	−0.3771	0.6261	0.1256	.656	−0.4133	0.5993
HDAH	-	PA0321	0.6463**	.009	0.2003	0.8704	0.6404*	.010	0.1906	0.8679
HDAH	-	PA1409	0.8694***	< .001	0.6439	0.9559	0.8602***	< .001	0.6221	0.9527
HDAH	-	PA3774	0.7126**	.003	0.3156	0.8973	0.6703**	.006	0.2406	0.8803
PA0321	-	PA1409	0.6325*	.011	0.1779	0.8646	0.6229*	.013	0.1624	0.8606
PA0321	-	PA3774	0.7493**	.001	0.3847	0.9116	0.6067*	.016	0.1370	0.8536
PA1409	-	PA3774	0.7494**	.001	0.3849	0.9117	0.6871**	.005	0.2697	0.8871

* p < .05, ** p < .01, *** p < .001

Table S3: Steiger Tests of correlations between class I HDACs and HDAC4 variants, n=26.

		HDAC1 / $HDAC4_{H976A}$	HDAC1 / $HDAC4_{L728A}$	HDAC1 / $HDAC4_{P809A}$
HDAC1 / $HDAC4_{H976Y}$	Steiger's z	4.07	4.29	4.03
	p	<0.001	<0.001	<0.001

		HDAC2 / $HDAC4_{H976A}$	HDAC2 / $HDAC4_{L728A}$	HDAC2 / $HDAC4_{P809A}$
HDAC2 / $HDAC4_{H976Y}$	Steiger's z	3.726	4.027	3.70
	p	<0.001	<0.001	<0.001

		HDAC3 / $HDAC4_{H976A}$	HDAC3 / $HDAC4_{L728A}$	HDAC3 / $HDAC4_{P809A}$
HDAC3 / $HDAC4_{H976Y}$	Steiger's z	3.44	3.69	3.32
	p	<0.001	<0.001	<0.001

The Steiger method (two-sided) was used as documented in cocor (https://search.r-project.org/CRAN/refmans/cocor/html/cocor.dep.groups.overlap.html)

Table S4: Benjamini-Hochberg correction across all unique correlation pairs at FDR = 0.05. Method reference: https://search.r-project.org/R/refmans/stats/html/p.adjust.html

Variable 1	Variable 2	Pearson r	Reported p	p used / bound	BH adjusted p / bound	Value type	FDR <= .05
HDAC2	HDAC1	0.868000	< .001	0.001000	0.006633	Upper bound	Significant
HDAC3	HDAC1	0.905900	< .001	0.001000	0.006633	Upper bound	Significant
HDAC3	HDAC2	0.741500	< .001	0.001000	0.006633	Upper bound	Significant
HDAC7	HDAC4	0.847100	< .001	0.001000	0.006633	Upper bound	Significant
HDAC7	HDAC5	0.961800	< .001	0.001000	0.006633	Upper bound	Significant
HDAC6	HDAC1	0.670200	< .001	0.001000	0.006633	Upper bound	Significant
HDAC6	HDAC2	0.660500	< .001	0.001000	0.006633	Upper bound	Significant
HDAC10	HDAC4	0.749400	< .001	0.001000	0.006633	Upper bound	Significant
drHDAC10	HDAC2	0.622400	< .001	0.001000	0.006633	Upper bound	Significant
drHDAC10	HDAC10	0.779400	< .001	0.001000	0.006633	Upper bound	Significant
HDAC8 C102S	HDAC8	0.662500	< .001	0.001000	0.006633	Upper bound	Significant
HDAC8 C153S	HDAC1	0.616800	< .001	0.001000	0.006633	Upper bound	Significant
HDAC8 C153S	HDAC8 C102S	0.633500	< .001	0.001000	0.006633	Upper bound	Significant
HDAC8 C125S	HDAC8	0.750900	< .001	0.001000	0.006633	Upper bound	Significant
HDAC8 C125S	HDAC8 C102S	0.933900	< .001	0.001000	0.006633	Upper bound	Significant
HDAC8 C125S	HDAC8 C153S	0.744300	< .001	0.001000	0.006633	Upper bound	Significant
HDAC8 S39E	HDAC8	0.665400	< .001	0.001000	0.006633	Upper bound	Significant
HDAC8 S39E	HDAC5	0.831300	< .001	0.001000	0.006633	Upper bound	Significant
HDAC8 S39E	HDAC7	0.683200	0.001	0.001000	0.006633	From reported p	Significant
HDAC8 S39E	HDAC8 C102S	0.968500	< .001	0.001000	0.006633	Upper bound	Significant
HDAC8 S39E	HDAC8 C153S	0.618900	< .001	0.001000	0.006633	Upper bound	Significant
HDAC8 S39E	HDAC8 C125S	0.922300	< .001	0.001000	0.006633	Upper bound	Significant
HDAC4 H976Y	HDAC1	0.761900	< .001	0.001000	0.006633	Upper bound	Significant
HDAC4 H976Y	HDAC2	0.731600	< .001	0.001000	0.006633	Upper bound	Significant
HDAC4 H976Y	HDAC3	0.704100	< .001	0.001000	0.006633	Upper bound	Significant
HDAC4 H976Y	HDAC4	0.615500	< .001	0.001000	0.006633	Upper bound	Significant
HDAC4 H976Y	HDAC6	0.805700	< .001	0.001000	0.006633	Upper bound	Significant
HDAC4 H976Y	drHDAC10	0.603600	0.001	0.001000	0.006633	From reported p	Significant
HDAC4 H976Y	HDAC8 C153S	0.643000	< .001	0.001000	0.006633	Upper bound	Significant
HDAC4 L728A	HDAC4	0.801800	< .001	0.001000	0.006633	Upper bound	Significant
HDAC4 L728A	HDAC5	0.963200	< .001	0.001000	0.006633	Upper bound	Significant
HDAC4 L728A	HDAC7	0.952700	< .001	0.001000	0.006633	Upper bound	Significant
HDAC4 P809A	HDAC4	0.757700	< .001	0.001000	0.006633	Upper bound	Significant
HDAC4 P809A	HDAC7	0.832100	< .001	0.001000	0.006633	Upper bound	Significant
HDAC4 P809A	HDAC4 L728A	0.898100	< .001	0.001000	0.006633	Upper bound	Significant
HDAC4 H976A	HDAC4	0.808400	< .001	0.001000	0.006633	Upper bound	Significant
HDAC4 H976A	HDAC5	0.915800	< .001	0.001000	0.006633	Upper bound	Significant
HDAC4 H976A	HDAC7	0.920000	< .001	0.001000	0.006633	Upper bound	Significant
HDAC4 H976A	HDAC4 L728A	0.968000	< .001	0.001000	0.006633	Upper bound	Significant
HDAC4 H976A	HDAC4 P809A	0.936800	< .001	0.001000	0.006633	Upper bound	Significant
HDAC11 4A	HDAC11	0.937100	< .001	0.001000	0.006633	Upper bound	Significant
HDAC11 7A	HDAC11	0.934500	< .001	0.001000	0.006633	Upper bound	Significant

HDAC11 7A	HDAC11 4A	0.921400	< .001	0.001000	0.006633	Upper bound	Significant
HDAC11 8A	HDAC11	0.933000	< .001	0.001000	0.006633	Upper bound	Significant
HDAC11 8A	HDAC11 4A	0.937100	< .001	0.001000	0.006633	Upper bound	Significant
HDAC11 8A	HDAC11 7A	0.880100	< .001	0.001000	0.006633	Upper bound	Significant
PA1409	HDAH	0.869400	< .001	0.001000	0.006633	Upper bound	Significant
PA3774	PA0321	0.749300	0.001	0.001000	0.006633	From reported p	Significant
PA3774	PA1409	0.749400	0.001	0.001000	0.006633	From reported p	Significant
HDAC6	HDAC3	0.570600	0.002	0.002000	0.012264	From reported p	Significant
HDAC8 S39E	HDAC10	0.591100	0.002	0.002000	0.012264	From reported p	Significant
HDAC8 S39E	drHDAC10	0.584400	0.002	0.002000	0.012264	From reported p	Significant
PA0321	HDAC4 H976A	0.720800	0.002	0.002000	0.012264	From reported p	Significant
HDAC5	HDAC4	0.776200	0.003	0.003000	0.016810	From reported p	Significant
HDAC8 C153S	HDAC6	0.564200	0.003	0.003000	0.016810	From reported p	Significant
HDAC4 H976A	HDAC10	0.565500	0.003	0.003000	0.016810	From reported p	Significant
PA0321	HDAC7	0.777800	0.003	0.003000	0.016810	From reported p	Significant
PA3774	HDAH	0.712600	0.003	0.003000	0.016810	From reported p	Significant
drHDAC10	HDAC4	0.543200	0.004	0.004000	0.020635	From reported p	Significant
HDAC8 C102S	HDAC7	0.623800	0.004	0.004000	0.020635	From reported p	Significant
HDAC8 C102S	drHDAC10	0.546000	0.004	0.004000	0.020635	From reported p	Significant
HDAC8 C153S	HDAC2	0.539400	0.004	0.004000	0.020635	From reported p	Significant
HDAC8 C153S	HDAC8	0.545400	0.004	0.004000	0.020635	From reported p	Significant
HDAC10	HDAC7	0.636200	0.005	0.005000	0.025000	From reported p	Significant
HDAC4 L728A	HDAC10	0.548700	0.005	0.005000	0.025000	From reported p	Significant
PA0321	HDAC4	0.670700	0.006	0.006000	0.029104	From reported p	Significant
PA0321	HDAC4 L728A	0.668400	0.006	0.006000	0.029104	From reported p	Significant
HDAC8 C102S	HDAC10	0.527000	0.007	0.007000	0.033456	From reported p	Significant
HDAC8 C153S	drHDAC10	0.505700	0.008	0.008000	0.036111	From reported p	Significant
HDAC8 S39E	HDAC4	0.509500	0.008	0.008000	0.036111	From reported p	Significant
HDAC4 P809A	HDAC5	0.719300	0.008	0.008000	0.036111	From reported p	Significant
PA0321	HDAC4 P809A	0.653400	0.008	0.008000	0.036111	From reported p	Significant
HDAC8 C125S	drHDAC10	0.503800	0.009	0.009000	0.039527	From reported p	Significant
PA0321	HDAH	0.646300	0.009	0.009000	0.039527	From reported p	Significant
drHDAC10	HDAC5	0.711200	0.01	0.010000	0.042763	From reported p	Significant
PA0321	HDAC10	0.661100	0.01	0.010000	0.042763	From reported p	Significant
HDAC8 C125S	HDAC10	0.498000	0.011	0.011000	0.045833	From reported p	Significant
PA1409	PA0321	0.632500	0.011	0.011000	0.045833	From reported p	Significant
HDAC8 C125S	HDAC7	0.565400	0.012	0.012000	0.049367	From reported p	Significant
HDAC4 P809A	HDAC10	0.490100	0.013	0.013000	0.052813	From reported p	Not significant

HDAC10	HDAC5	0.685800	0.014	0.014000	0.054819	From reported p	Not significant
HDAC8 C125S	HDAC5	0.685800	0.014	0.014000	0.054819	From reported p	Not significant
HDAC4 H976Y	HDAC10	0.484500	0.014	0.014000	0.054819	From reported p	Not significant
HDAC8 C102S	HDAC4	0.470500	0.015	0.015000	0.057353	From reported p	Not significant
HDAC8 C125S	HDAC4	0.470400	0.015	0.015000	0.057353	From reported p	Not significant
PA0321	HDAC5	0.733200	0.016	0.016000	0.060465	From reported p	Not significant
HDAC4 L728A	HDAC8 S39E	0.458500	0.018	0.018000	0.067241	From reported p	Not significant
HDAC8 C153S	HDAC3	0.457500	0.019	0.019000	0.070170	From reported p	Not significant
drHDAC10	HDAC1	0.445600	0.023	0.023000	0.083056	From reported p	Not significant
HDAC8 C102S	HDAC5	0.648000	0.023	0.023000	0.083056	From reported p	Not significant
HDAC5	HDAC8	0.643200	0.024	0.024000	0.085714	From reported p	Not significant
PA0321	HDAC8 C153S	0.574700	0.025	0.025000	0.088315	From reported p	Not significant
HDAC4 L728A	HDAC8 C102S	0.434400	0.027	0.027000	0.094355	From reported p	Not significant
PA0321	HDAC8 C125S	0.558400	0.03	0.030000	0.103723	From reported p	Not significant
HDAC8 C125S	HDAC1	0.419100	0.033	0.033000	0.112895	From reported p	Not significant
HDAC8	HDAC3	0.418100	0.034	0.034000	0.115104	From reported p	Not significant
PA0321	HDAC8 S39E	0.539200	0.038	0.038000	0.127320	From reported p	Not significant
PA0321	HDAC8 C102S	0.514600	0.05	0.050000	0.165816	From reported p	Not significant
HDAC4	HDAC3	0.386600	0.051	0.051000	0.167424	From reported p	Not significant
HDAC4 H976Y	HDAC8 C125S	0.385800	0.052	0.052000	0.169000	From reported p	Not significant
HDAC8 C125S	HDAC3	0.381700	0.054	0.054000	0.170388	From reported p	Not significant
HDAC8 S39E	HDAC1	0.382000	0.054	0.054000	0.170388	From reported p	Not significant
HDAC11 4A	HDAC8 C153S	0.382800	0.054	0.054000	0.170388	From reported p	Not significant
drHDAC10	HDAC7	0.445800	0.056	0.056000	0.175000	From reported p	Not significant
HDAC4 L728A	drHDAC10	0.375100	0.059	0.059000	0.182619	From reported p	Not significant
HDAC8 C153S	HDAC10	0.364800	0.073	0.073000	0.221729	From reported p	Not significant
HDAC4 H976A	HDAC8 S39E	0.356900	0.073	0.073000	0.221729	From reported p	Not significant
HDAC4 L728A	HDAC8 C125S	0.352800	0.077	0.077000	0.231713	From reported p	Not significant
HDAC8 S39E	HDAC3	0.349200	0.08	0.080000	0.237162	From reported p	Not significant
HDAC4 H976A	drHDAC10	0.348200	0.081	0.081000	0.237162	From reported p	Not significant
HDAC11 8A	HDAC6	0.348800	0.081	0.081000	0.237162	From reported p	Not significant
drHDAC10	HDAC3	0.345500	0.084	0.084000	0.243750	From reported p	Not significant
HDAC11 8A	HDAC8 C153S	0.342800	0.086	0.086000	0.245798	From reported p	Not significant
HDAC4	HDAC1	0.340600	0.089	0.089000	0.245798	From reported p	Not significant
HDAC8 C102S	HDAC1	0.340200	0.089	0.089000	0.245798	From reported p	Not significant
HDAC4 H976Y	HDAC8 S39E	0.339900	0.089	0.089000	0.245798	From reported p	Not significant
HDAC11 4A	HDAC6	0.339800	0.089	0.089000	0.245798	From reported p	Not significant

HDAC11 4A	HDAC8 C125S	0.339600	0.09	0.090000	0.245798	From reported p	Not significant
HDAH	HDAC6	0.453300	0.09	0.090000	0.245798	From reported p	Not significant
HDAH	HDAC4 H976Y	0.441900	0.099	0.099000	0.265909	From reported p	Not significant
PA0321	HDAC1	0.441800	0.099	0.099000	0.265909	From reported p	Not significant
PA0321	HDAC4 H976Y	0.441100	0.1	0.100000	0.266393	From reported p	Not significant
HDAC4 H976A	HDAC8 C102S	0.325200	0.105	0.105000	0.277439	From reported p	Not significant
HDAC11 8A	HDAC4 H976Y	0.318300	0.113	0.113000	0.296169	From reported p	Not significant
HDAC11 4A	HDAC4 H976Y	0.313800	0.119	0.119000	0.309400	From reported p	Not significant
HDAC4	HDAC8	0.309700	0.124	0.124000	0.317323	From reported p	Not significant
HDAC7	HDAC8	0.365200	0.124	0.124000	0.317323	From reported p	Not significant
HDAC4 H976Y	HDAC7	0.364700	0.125	0.125000	0.317383	From reported p	Not significant
HDAC8 C153S	HDAC4	0.308000	0.126	0.126000	0.317442	From reported p	Not significant
HDAC11 4A	HDAC1	0.307500	0.127	0.127000	0.317500	From reported p	Not significant
HDAC11 8A	HDAC1	0.297900	0.139	0.139000	0.344549	From reported p	Not significant
HDAC6	HDAC4	0.296800	0.141	0.141000	0.344549	From reported p	Not significant
HDAC8 C153S	HDAC5	0.451300	0.141	0.141000	0.344549	From reported p	Not significant
drHDAC10	HDAC6	0.294900	0.144	0.144000	0.349254	From reported p	Not significant
HDAC4 P809A	drHDAC10	0.286900	0.155	0.155000	0.372101	From reported p	Not significant
HDAC11 4A	HDAC8 C102S	0.285000	0.158	0.158000	0.372101	From reported p	Not significant
HDAC11 4A	HDAC8 S39E	0.285000	0.158	0.158000	0.372101	From reported p	Not significant
HDAH	HDAC1	0.383500	0.158	0.158000	0.372101	From reported p	Not significant
HDAC8 S39E	HDAC2	0.283500	0.161	0.161000	0.375709	From reported p	Not significant
HDAC4 L728A	HDAC4 H976Y	0.282600	0.162	0.162000	0.375709	From reported p	Not significant
PA1409	HDAC6	0.379500	0.163	0.163000	0.375709	From reported p	Not significant
PA1409	HDAC8 C153S	0.375900	0.167	0.167000	0.382218	From reported p	Not significant
PA0321	HDAC6	0.371100	0.173	0.173000	0.393182	From reported p	Not significant
HDAC11 8A	HDAC2	0.273300	0.177	0.177000	0.396233	From reported p	Not significant
PA1409	HDAC4 H976Y	0.367800	0.177	0.177000	0.396233	From reported p	Not significant
PA0321	HDAC3	0.367700	0.178	0.178000	0.396233	From reported p	Not significant
HDAC10	HDAC2	0.276100	0.182	0.182000	0.402381	From reported p	Not significant
HDAC4 P809A	HDAC8 S39E	0.267800	0.186	0.186000	0.407886	From reported p	Not significant
HDAC8	HDAC1	0.267300	0.187	0.187000	0.407886	From reported p	Not significant
PA1409	HDAC1	0.357600	0.191	0.191000	0.413833	From reported p	Not significant
HDAC4 H976Y	HDAC8 C102S	0.262400	0.195	0.195000	0.419702	From reported p	Not significant
HDAC10	HDAC1	0.266600	0.198	0.198000	0.423355	From reported p	Not significant
HDAC4 H976A	HDAC4 H976Y	0.256200	0.207	0.207000	0.438961	From reported p	Not significant
HDAC8 C102S	HDAC3	0.255500	0.208	0.208000	0.438961	From reported p	Not significant

HDAC11 4A	HDAC7	0.300500	0.211	0.211000	0.442419	From reported p	Not significant
HDAC8 C125S	HDAC2	0.251700	0.215	0.215000	0.447917	From reported p	Not significant
HDAC5	HDAC3	0.381400	0.221	0.221000	0.457484	From reported p	Not significant
HDAC11 4A	HDAC2	0.245200	0.227	0.227000	0.466930	From reported p	Not significant
HDAH	HDAC8 C153S	0.327900	0.233	0.233000	0.476258	From reported p	Not significant
HDAC11 4A	drHDAC10	0.239700	0.238	0.238000	0.483438	From reported p	Not significant
HDAC4 P809A	HDAC8 C102S	0.238300	0.241	0.241000	0.485494	From reported p	Not significant
HDAC4 P809A	HDAC4 H976Y	0.237600	0.242	0.242000	0.485494	From reported p	Not significant
PA1409	HDAC3	0.315000	0.253	0.253000	0.502273	From reported p	Not significant
HDAH	HDAC3	0.314500	0.254	0.254000	0.502273	From reported p	Not significant
PA0321	HDAC8	0.313400	0.255	0.255000	0.502273	From reported p	Not significant
HDAC11 7A	HDAC8 C102S	0.230000	0.258	0.258000	0.505120	From reported p	Not significant
drHDAC10	HDAC8	0.227400	0.264	0.264000	0.513772	From reported p	Not significant
HDAC8 C102S	HDAC2	0.224900	0.269	0.269000	0.520387	From reported p	Not significant
HDAC4 H976A	HDAC8 C125S	0.221300	0.277	0.277000	0.532692	From reported p	Not significant
HDAC4	HDAC2	0.219000	0.282	0.282000	0.538517	From reported p	Not significant
HDAC11 7A	HDAC7	0.258600	0.285	0.285000	0.538517	From reported p	Not significant
HDAC11 8A	HDAC8 C125S	0.217700	0.285	0.285000	0.538517	From reported p	Not significant
HDAC8 C153S	HDAC11	0.207200	0.31	0.310000	0.579023	From reported p	Not significant
HDAC11 7A	HDAC8 C125S	0.207300	0.31	0.310000	0.579023	From reported p	Not significant
HDAC4 H976Y	HDAC11	0.204500	0.316	0.316000	0.586857	From reported p	Not significant
HDAC10	HDAC3	0.204200	0.327	0.327000	0.600424	From reported p	Not significant
HDAC4 H976Y	HDAC5	0.309900	0.327	0.327000	0.600424	From reported p	Not significant
HDAC11 8A	drHDAC10	0.198600	0.331	0.331000	0.603315	From reported p	Not significant
HDAC11	HDAC1	0.197400	0.334	0.334000	0.603315	From reported p	Not significant
HDAC11 7A	HDAC8 S39E	0.196800	0.335	0.335000	0.603315	From reported p	Not significant
HDAC11 4A	HDAC10	0.200800	0.336	0.336000	0.603315	From reported p	Not significant
HDAC7	HDAC3	0.232000	0.339	0.339000	0.604324	From reported p	Not significant
HDAC11 8A	HDAC10	0.197800	0.343	0.343000	0.604324	From reported p	Not significant
HDAC11	HDAC6	0.193300	0.344	0.344000	0.604324	From reported p	Not significant
HDAH	HDAC11 8A	0.262600	0.344	0.344000	0.604324	From reported p	Not significant
HDAC11 4A	HDAC5	0.296500	0.349	0.349000	0.609812	From reported p	Not significant
HDAH	HDAC7	0.294600	0.353	0.353000	0.610239	From reported p	Not significant
PA3774	drHDAC10	-0.258200	0.353	0.353000	0.610239	From reported p	Not significant
PA3774	HDAC4 H976A	0.255700	0.358	0.358000	0.615608	From reported p	Not significant
HDAH	HDAC4 H976A	0.251500	0.366	0.366000	0.624609	From reported p	Not significant
HDAH	HDAC5	0.319500	0.368	0.368000	0.624609	From reported p	Not significant

HDAC8 C153S	HDAC7	0.218600	0.369	0.369000	0.624609	From reported p	Not significant
HDAC11 7A	HDAC1	0.178300	0.384	0.384000	0.644974	From reported p	Not significant
HDAC4 H976Y	HDAC8	0.177600	0.385	0.385000	0.644974	From reported p	Not significant
HDAC7	HDAC1	0.205200	0.399	0.399000	0.665000	From reported p	Not significant
PA1409	HDAC4 H976A	0.233500	0.402	0.402000	0.666497	From reported p	Not significant
HDAC10	HDAC8	0.174700	0.404	0.404000	0.666497	From reported p	Not significant
HDAC8 C125S	HDAC11	0.168600	0.41	0.410000	0.672980	From reported p	Not significant
HDAC8 C125S	HDAC6	0.166500	0.416	0.416000	0.679250	From reported p	Not significant
PA3774	HDAC8 C153S	0.226100	0.418	0.418000	0.679250	From reported p	Not significant
HDAC11 7A	HDAC8 C153S	0.160700	0.433	0.433000	0.700124	From reported p	Not significant
HDAC4 P809A	HDAC8 C125S	0.157500	0.442	0.442000	0.709236	From reported p	Not significant
drHDAC10	HDAC11	0.157300	0.443	0.443000	0.709236	From reported p	Not significant
HDAC5	HDAC1	0.242700	0.447	0.447000	0.712132	From reported p	Not significant
PA3774	HDAC5	0.270400	0.45	0.450000	0.712500	From reported p	Not significant
HDAC11	HDAC2	0.153300	0.455	0.455000	0.712500	From reported p	Not significant
HDAC11 7A	HDAC6	0.152800	0.456	0.456000	0.712500	From reported p	Not significant
PA3774	HDAC1	0.208300	0.456	0.456000	0.712500	From reported p	Not significant
HDAC8 C102S	HDAC11	0.147400	0.472	0.472000	0.727169	From reported p	Not significant
PA0321	HDAC11 4A	0.200100	0.475	0.475000	0.727169	From reported p	Not significant
PA3774	HDAC6	0.199100	0.477	0.477000	0.727169	From reported p	Not significant
PA3774	HDAC7	0.225400	0.481	0.481000	0.727169	From reported p	Not significant
HDAH	HDAC4 L728A	0.196100	0.484	0.484000	0.727169	From reported p	Not significant
HDAC11 8A	HDAC8 C102S	0.142800	0.486	0.486000	0.727169	From reported p	Not significant
HDAH	HDAC11 4A	0.195200	0.486	0.486000	0.727169	From reported p	Not significant
HDAC11 8A	HDAC8 S39E	0.142500	0.487	0.487000	0.727169	From reported p	Not significant
HDAC10	HDAC6	0.145500	0.488	0.488000	0.727169	From reported p	Not significant
PA0321	drHDAC10	0.194200	0.488	0.488000	0.727169	From reported p	Not significant
HDAC4 L728A	HDAC8	0.141700	0.49	0.490000	0.727169	From reported p	Not significant
HDAC11 4A	HDAC4	0.140500	0.494	0.494000	0.729773	From reported p	Not significant
HDAH	HDAC4	0.189900	0.498	0.498000	0.732353	From reported p	Not significant
HDAH	HDAC8 C102S	-0.188000	0.502	0.502000	0.734910	From reported p	Not significant
PA1409	drHDAC10	-0.186100	0.507	0.507000	0.738901	From reported p	Not significant
PA1409	HDAC5	0.230800	0.521	0.521000	0.752416	From reported p	Not significant
PA3774	HDAC11 4A	0.180000	0.521	0.521000	0.752416	From reported p	Not significant
HDAC11 4A	HDAC3	0.130700	0.525	0.525000	0.752416	From reported p	Not significant
HDAC8 S39E	HDAC6	0.129500	0.528	0.528000	0.752416	From reported p	Not significant
HDAC4 L728A	HDAC6	0.127200	0.536	0.536000	0.752416	From reported p	Not significant

PA3774	HDAC4 P809A	0.173600	0.536	0.536000	0.752416	From reported p	Not significant
HDAC6	HDAC7	0.151100	0.537	0.537000	0.752416	From reported p	Not significant
HDAC8 S39E	HDAC11	0.126800	0.537	0.537000	0.752416	From reported p	Not significant
PA0321	HDAC11 8A	0.171800	0.54	0.540000	0.752416	From reported p	Not significant
PA3774	HDAC4 L728A	0.169000	0.547	0.547000	0.752416	From reported p	Not significant
HDAH	HDAC8	-0.168700	0.548	0.548000	0.752416	From reported p	Not significant
HDAH	HDAC2	0.168300	0.549	0.549000	0.752416	From reported p	Not significant
HDAC11 7A	drHDAC10	0.122400	0.551	0.551000	0.752416	From reported p	Not significant
HDAH	drHDAC10	-0.167300	0.551	0.551000	0.752416	From reported p	Not significant
PA3774	HDAC11 8A	0.167500	0.551	0.551000	0.752416	From reported p	Not significant
PA1409	HDAC2	0.164500	0.558	0.558000	0.754752	From reported p	Not significant
PA3774	HDAC3	0.163800	0.56	0.560000	0.754752	From reported p	Not significant
PA1409	HDAC7	0.186600	0.561	0.561000	0.754752	From reported p	Not significant
PA0321	HDAC11 7A	0.163000	0.562	0.562000	0.754752	From reported p	Not significant
HDAH	HDAC11	0.160800	0.567	0.567000	0.758333	From reported p	Not significant
HDAC11 8A	HDAC3	0.116800	0.57	0.570000	0.759221	From reported p	Not significant
HDAC6	HDAC5	0.181100	0.573	0.573000	0.760102	From reported p	Not significant
HDAC11 8A	HDAC4 P809A	-0.113800	0.58	0.580000	0.766260	From reported p	Not significant
HDAC4 L728A	HDAC8 C153S	0.112300	0.585	0.585000	0.769737	From reported p	Not significant
PA1409	HDAC4	0.152200	0.588	0.588000	0.770565	From reported p	Not significant
PA1409	HDAC4 L728A	0.149600	0.595	0.595000	0.776606	From reported p	Not significant
HDAC4 H976A	HDAC6	0.107400	0.601	0.601000	0.778187	From reported p	Not significant
PA3774	HDAC4 H976Y	0.147000	0.601	0.601000	0.778187	From reported p	Not significant
PA3774	HDAC11	0.145700	0.604	0.604000	0.778968	From reported p	Not significant
PA1409	HDAC11 7A	-0.143400	0.61	0.610000	0.782549	From reported p	Not significant
HDAC11 8A	HDAC4 H976A	-0.104400	0.612	0.612000	0.782549	From reported p	Not significant
HDAC11	HDAC7	0.123800	0.614	0.614000	0.782549	From reported p	Not significant
HDAC11 8A	HDAC5	-0.160300	0.619	0.619000	0.785840	From reported p	Not significant
HDAC4 P809A	HDAC11	-0.100500	0.625	0.625000	0.790370	From reported p	Not significant
HDAC11 7A	HDAC10	0.100300	0.633	0.633000	0.795000	From reported p	Not significant
HDAC4 P809A	HDAC8 C153S	-0.098110	0.634	0.634000	0.795000	From reported p	Not significant
PA3774	HDAC4	0.133400	0.636	0.636000	0.795000	From reported p	Not significant
PA3774	HDAC10	0.135100	0.645	0.645000	0.803161	From reported p	Not significant
HDAC11 7A	HDAC4 H976Y	0.093440	0.65	0.650000	0.806298	From reported p	Not significant
HDAC11 7A	HDAC2	0.092230	0.654	0.654000	0.808175	From reported p	Not significant
HDAC11	HDAC10	0.092230	0.661	0.661000	0.813731	From reported p	Not significant
PA0321	HDAC11	0.121800	0.666	0.666000	0.816792	From reported p	Not significant

HDAC4 P809A	HDAC6	0.084290	0.682	0.682000	0.833271	From reported p	Not significant
HDAH	HDAC4 P809A	0.112900	0.689	0.689000	0.838670	From reported p	Not significant
PA3774	HDAC11 7A	0.111500	0.692	0.692000	0.839179	From reported p	Not significant
HDAC11 8A	HDAC4 L728A	-0.079110	0.701	0.701000	0.846933	From reported p	Not significant
HDAC8	HDAC2	0.076780	0.709	0.709000	0.853426	From reported p	Not significant
HDAH	HDAC10	0.107800	0.714	0.714000	0.854320	From reported p	Not significant
HDAC11 4A	HDAC8	0.075180	0.715	0.715000	0.854320	From reported p	Not significant
HDAH	HDAC8 S39E	-0.099030	0.725	0.725000	0.863095	From reported p	Not significant
PA1409	HDAC8	0.095940	0.734	0.734000	0.865489	From reported p	Not significant
PA3774	HDAC2	-0.095750	0.734	0.734000	0.865489	From reported p	Not significant
HDAC4 H976A	HDAC11	-0.069730	0.735	0.735000	0.865489	From reported p	Not significant
HDAC11 8A	HDAC8	-0.068280	0.74	0.740000	0.868231	From reported p	Not significant
PA0321	HDAC2	0.091970	0.744	0.744000	0.869784	From reported p	Not significant
PA1409	HDAC8 C102S	-0.089520	0.751	0.751000	0.874018	From reported p	Not significant
HDAC11	HDAC5	-0.101700	0.753	0.753000	0.874018	From reported p	Not significant
HDAC11 7A	HDAC4	0.063960	0.756	0.756000	0.874377	From reported p	Not significant
HDAC11 4A	HDAC4 P809A	-0.061350	0.766	0.766000	0.882801	From reported p	Not significant
HDAC11	HDAC4	0.060370	0.77	0.770000	0.884276	From reported p	Not significant
HDAC8 C102S	HDAC6	0.059290	0.774	0.774000	0.885739	From reported p	Not significant
HDAC11 8A	HDAC4	0.057740	0.779	0.779000	0.886364	From reported p	Not significant
HDAC4 P809A	HDAC1	-0.057630	0.78	0.780000	0.886364	From reported p	Not significant
HDAC4 L728A	HDAC11	-0.053950	0.794	0.794000	0.899129	From reported p	Not significant
HDAC7	HDAC2	0.060790	0.805	0.805000	0.908420	From reported p	Not significant
HDAC11	HDAC8	-0.049180	0.811	0.811000	0.912024	From reported p	Not significant
HDAC4 H976A	HDAC8	0.046710	0.821	0.821000	0.920086	From reported p	Not significant
HDAC11 7A	HDAC4 P809A	-0.045620	0.825	0.825000	0.921392	From reported p	Not significant
PA1409	HDAC4 P809A	0.058780	0.835	0.835000	0.929366	From reported p	Not significant
HDAC11 7A	HDAC5	0.061610	0.849	0.849000	0.937795	From reported p	Not significant
HDAC11 4A	HDAC4 L728A	0.039010	0.85	0.850000	0.937795	From reported p	Not significant
HDAC11	HDAC3	0.037630	0.855	0.855000	0.937795	From reported p	Not significant
HDAC11 7A	HDAC4 H976A	-0.037650	0.855	0.855000	0.937795	From reported p	Not significant
HDAC4 P809A	HDAC2	-0.037170	0.857	0.857000	0.937795	From reported p	Not significant
PA3774	HDAC8 C125S	0.049490	0.861	0.861000	0.938043	From reported p	Not significant
HDAC5	HDAC2	0.055860	0.863	0.863000	0.938043	From reported p	Not significant
HDAC4 L728A	HDAC3	0.032740	0.874	0.874000	0.944767	From reported p	Not significant
HDAC11 8A	HDAC7	0.038830	0.875	0.875000	0.944767	From reported p	Not significant
PA1409	HDAC8 S39E	-0.040520	0.886	0.886000	0.951480	From reported p	Not significant

PA1409	HDAC10	0.041670	0.888	0.888000	0.951480	From reported p	Not significant
HDAH	HDAC8 C125S	-0.039220	0.89	0.890000	0.951480	From reported p	Not significant
HDAH	HDAC11 7A	0.034440	0.903	0.903000	0.959121	From reported p	Not significant
PA1409	HDAC11 8A	0.034000	0.904	0.904000	0.959121	From reported p	Not significant
HDAC11 7A	HDAC4 L728A	-0.024450	0.906	0.906000	0.959121	From reported p	Not significant
HDAC4 H976A	HDAC3	0.022640	0.913	0.913000	0.963393	From reported p	Not significant
PA3774	HDAC8 C102S	-0.028830	0.919	0.919000	0.964516	From reported p	Not significant
HDAC11 7A	HDAC8	-0.020840	0.92	0.920000	0.964516	From reported p	Not significant
HDAC6	HDAC8	-0.018820	0.927	0.927000	0.968730	From reported p	Not significant
PA1409	HDAC11	-0.021060	0.941	0.941000	0.972956	From reported p	Not significant
PA3774	HDAC8 S39E	-0.020110	0.943	0.943000	0.972956	From reported p	Not significant
HDAC4 L728A	HDAC1	0.014570	0.944	0.944000	0.972956	From reported p	Not significant
PA1409	HDAC11 4A	0.019140	0.946	0.946000	0.972956	From reported p	Not significant
HDAC4 H976A	HDAC1	-0.013370	0.948	0.948000	0.972956	From reported p	Not significant
HDAC4 L728A	HDAC2	0.012480	0.952	0.952000	0.972956	From reported p	Not significant
HDAC11 4A	HDAC4 H976A	-0.012430	0.952	0.952000	0.972956	From reported p	Not significant
HDAC4 P809A	HDAC8	-0.011310	0.956	0.956000	0.973981	From reported p	Not significant
HDAC4 H976A	HDAC2	0.008546	0.967	0.967000	0.977019	From reported p	Not significant
PA3774	HDAC8	0.011810	0.967	0.967000	0.977019	From reported p	Not significant
HDAC11 7A	HDAC3	0.008257	0.968	0.968000	0.977019	From reported p	Not significant
HDAC4 H976A	HDAC8 C153S	0.004591	0.982	0.982000	0.988040	From reported p	Not significant
HDAC4 P809A	HDAC3	0.003988	0.985	0.985000	0.988040	From reported p	Not significant
PA1409	HDAC8 C125S	0.003260	0.991	0.991000	0.991000	From reported p	Not significant

Omit HDAC1

Recovered reference branches: 23/23

Recalculated average-linkage hierarchy

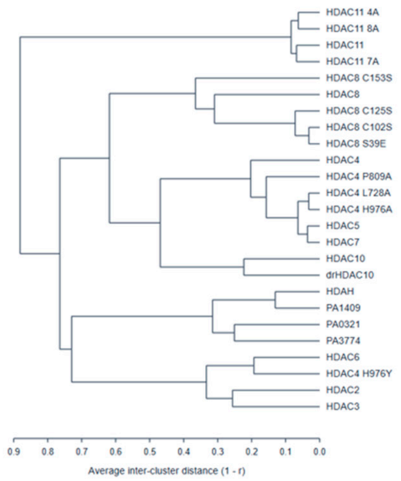

Omit HDAC2

Recovered reference branches: 23/23

Recalculated average-linkage hierarchy

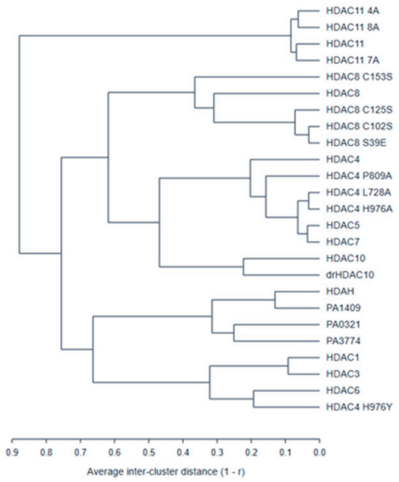

Omit HDAC3

Recovered reference branches: 23/23

Recalculated average-linkage hierarchy

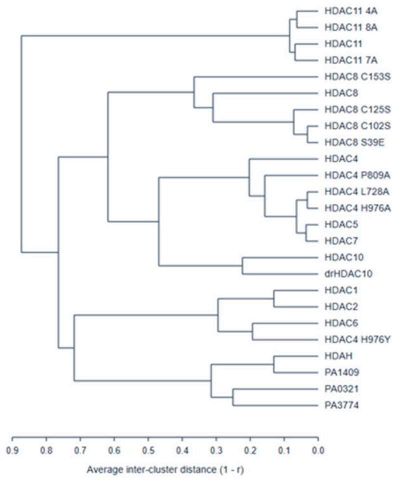

Omit HDAC8

Recovered reference branches: 23/23

Recalculated average-linkage hierarchy

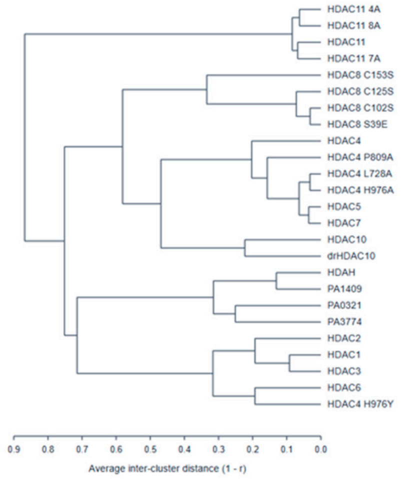

Omit HDAC4

Recovered reference branches: 23/23

Recalculated average-linkage hierarchy

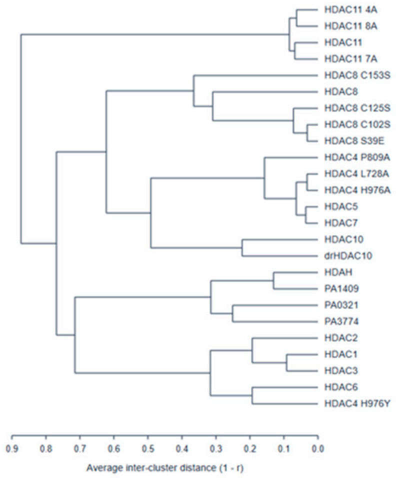

Omit HDAC5

Recovered reference branches: 23/23

Recalculated average-linkage hierarchy

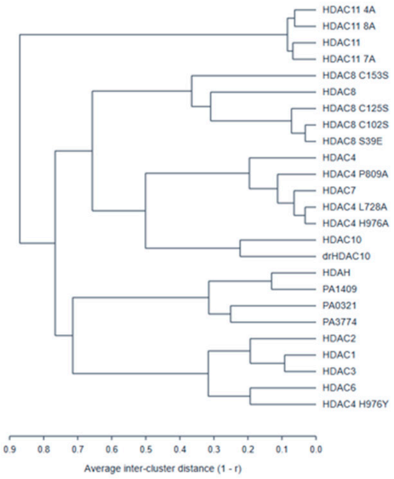

Omit HDAC7

Recovered reference branches: 23/23

Recalculated average-linkage hierarchy

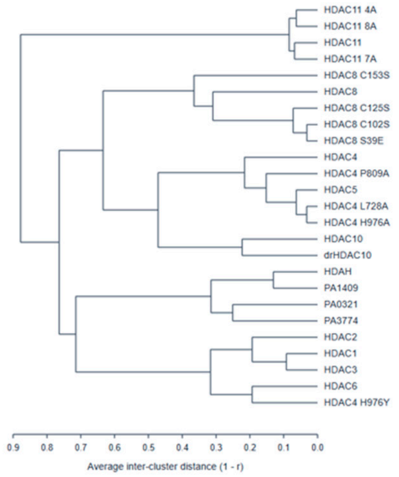

Omit HDAC6

Recovered reference branches: 22/23

Recalculated average-linkage hierarchy

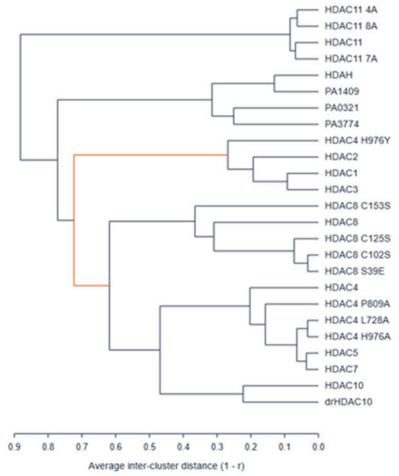

Omit HDAC10

Recovered reference branches: 22/23

Recalculated average-linkage hierarchy

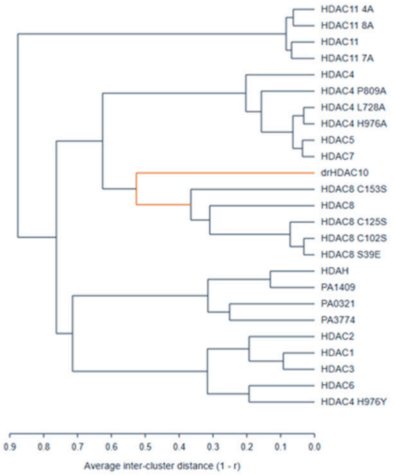

Omit HDAC11

Recovered reference branches: 23/23

Recalculated average-linkage hierarchy

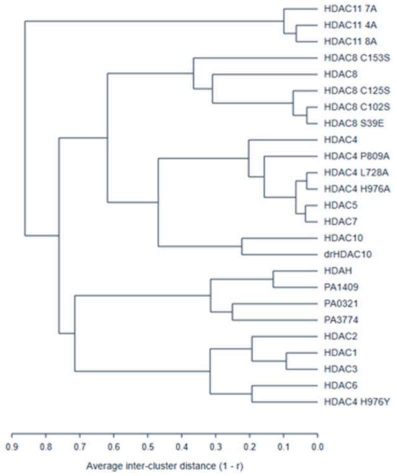

Omit drHDAC10

Recovered reference branches: 23/23

Recalculated average-linkage hierarchy

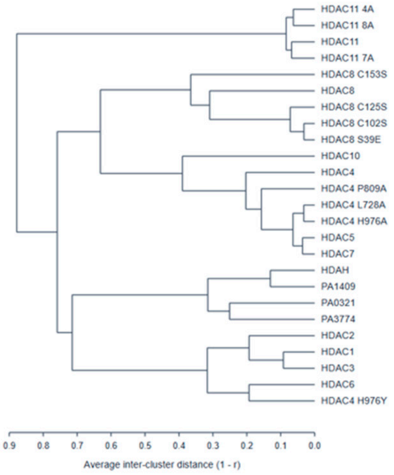

Omit HDAC8 C102S

Recovered reference branches: 23/23

Recalculated average-linkage hierarchy

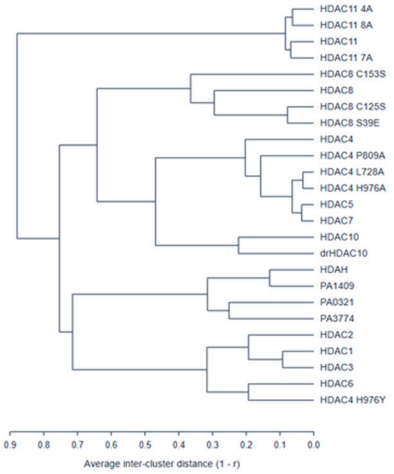

Omit HDAC8 C153S

Recovered reference branches: 23/23

Recalculated average-linkage hierarchy

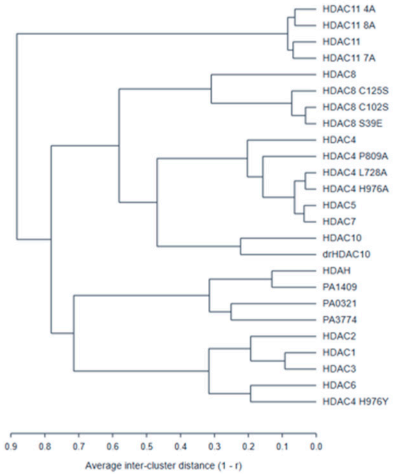

Omit HDAC8 C125S

Recovered reference branches: 23/23

Recalculated average-linkage hierarchy

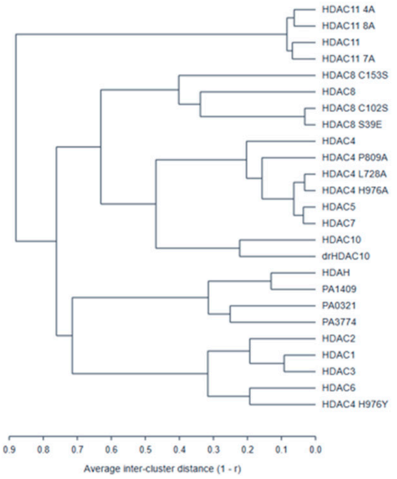

Omit HDAC8 S39E

Recovered reference branches: 23/23

Recalculated average-linkage hierarchy

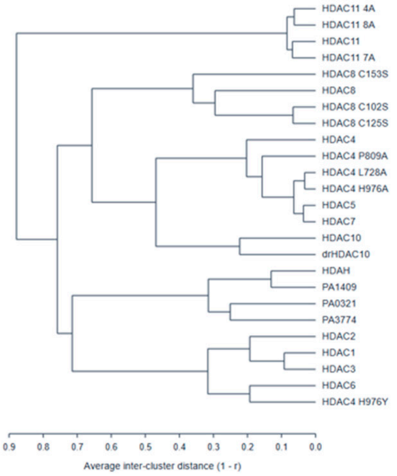

Omit HDAC4 H976Y

Recovered reference branches: 23/23

Recalculated average-linkage hierarchy

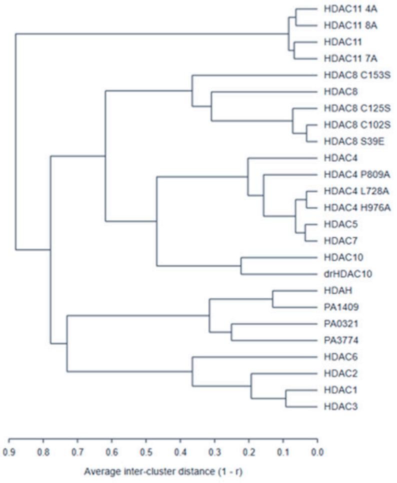

Omit HDAC4 L728A

Recovered reference branches: 22/23

Recalculated average-linkage hierarchy

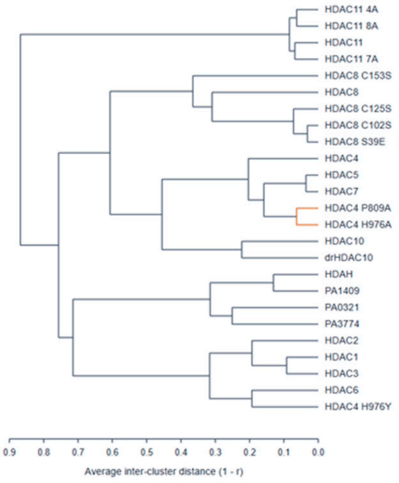

Omit HDAC4 P809A

Recovered reference branches: 23/23

Recalculated average-linkage hierarchy

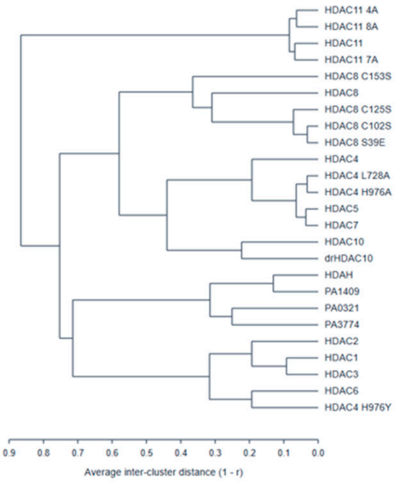

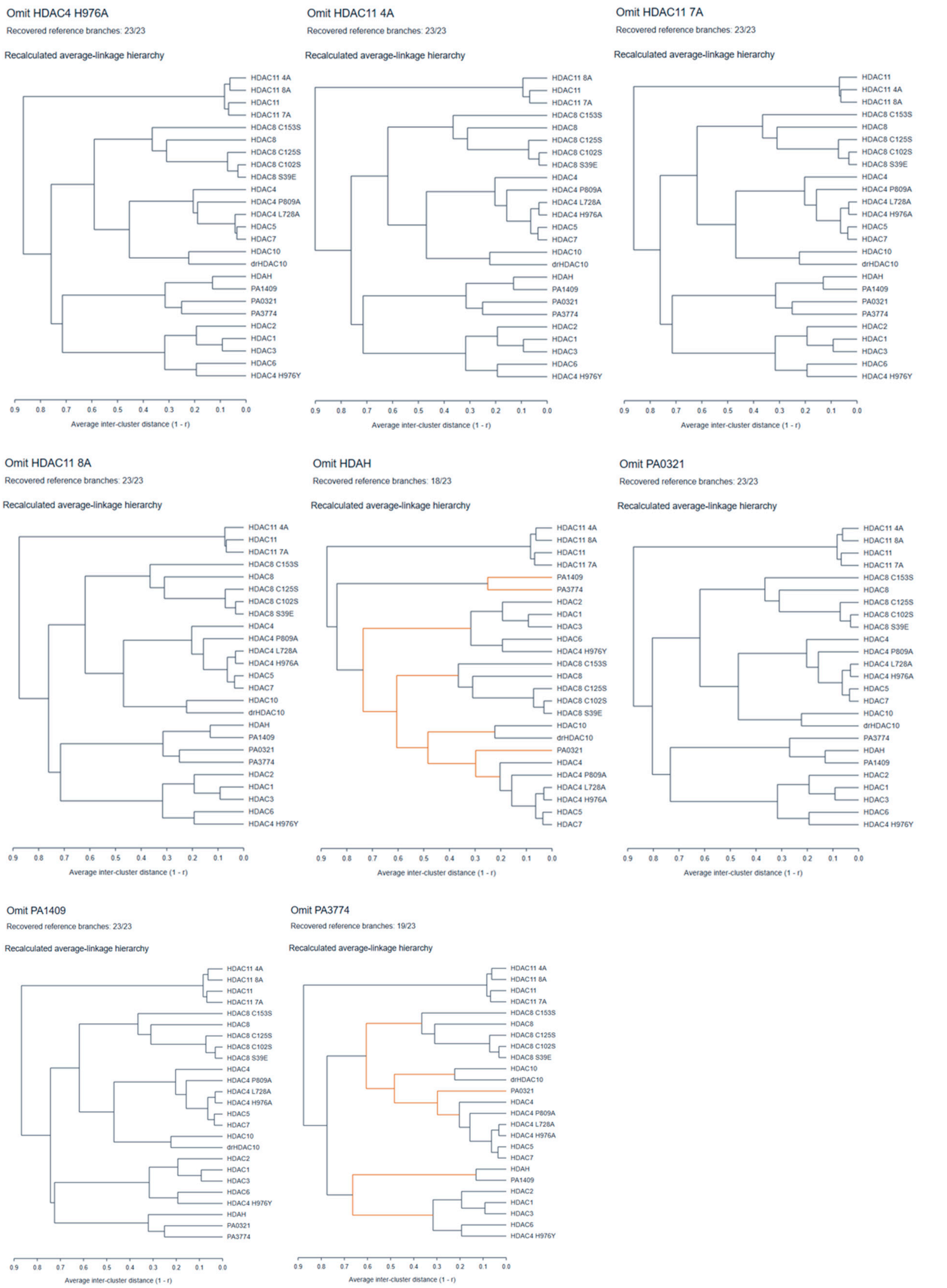

Figure S8: Leave-one-enzyme-out trees resulting from hierarchical clustering using average linkage and distance 1-r. Black lines mark branches that are preserved and orange lines those that are not preserved with respect to the original tree after pruning the omitted enzyme.

Table S5: Descriptive statistics of pIC50 data:

Descriptive Statistics

	HDAC1	HDAC2	HDAC3	HDAC8	HDAC4	HDAC5	HDAC7	HDAC6	HDAC10	HDAC11	drHDAC10	HDAC8 C102S	HDAC8 C153S	HDAC8 C125S	HDAC8 S39E	HDAC4 H976Y	HDAC4 L728A	HDAC4 P809A	HDAC4 H976A	HDAC11 4A	HDAC11 7A	HDAC11 8A	HDAH	PA0321	PA1409	PA3774
Valid	26	26	26	26	26	12	19	26	25	26	26	26	26	26	26	26	26	26	26	26	26	26	15	15	15	15
Missing	0	0	0	0	0	14	7	0	1	0	0	0	0	0	0	0	0	0	0	0	0	0	11	11	11	11
Mean (arithmetic)	6.190	5.832	6.226	6.025	5.420	5.129	5.389	6.205	5.016	4.991	5.084	4.892	5.660	5.247	5.062	6.064	5.473	5.134	5.365	5.301	4.970	5.261	6.059	5.867	6.343	6.737
Std. Deviation	1.397	1.327	1.434	1.052	1.183	0.9782	0.9083	1.364	1.109	0.9014	1.024	0.7045	0.8419	0.9254	0.8926	1.347	1.385	1.316	1.404	1.084	0.9646	0.9590	0.9194	1.079	1.481	1.487
Minimum	4.300	4.300	4.300	4.460	4.300	4.300	4.300	4.460	4.460	4.460	4.460	4.460	4.460	4.460	4.460	4.460	4.460	4.460	3.840	4.460	4.460	4.460	4.520	4.300	4.300	4.300
Maximum	9.000	8.400	8.700	8.100	8.300	7.230	7.330	9.000	8.400	8.520	8.050	6.520	7.000	7.280	7.390	9.000	8.700	9.000	9.000	9.000	9.000	8.520	7.260	8.380	8.550	8.820

Table S6: Docking scores for the indicated HDACs and their respective ligands. The compounds were docked into the crystal structures of HDAC2 (PDB ID 7KGB) or HDAC6 (PDB ID 5EDU) using MOE 2024.0601 with the induced fit setting, and the Amber:EHT force field was applied. The GBVI/WSA dG score of the optimal refined docking pose is provided.

Target	Ligand	GBVI/WSA dG Score	pIC50
HDAC2	Trichostatin A	-12.3	8.4
HDAC2	Panobinostat	-12.4	8.4
HDAC2	MB-463	-12.7	6.6
HDAC6	Trichostatin A	-12.4	9.0
HDAC6	Panobinostat	-12.4	8.2
HDAC6	MB-463	-12.6	6.7

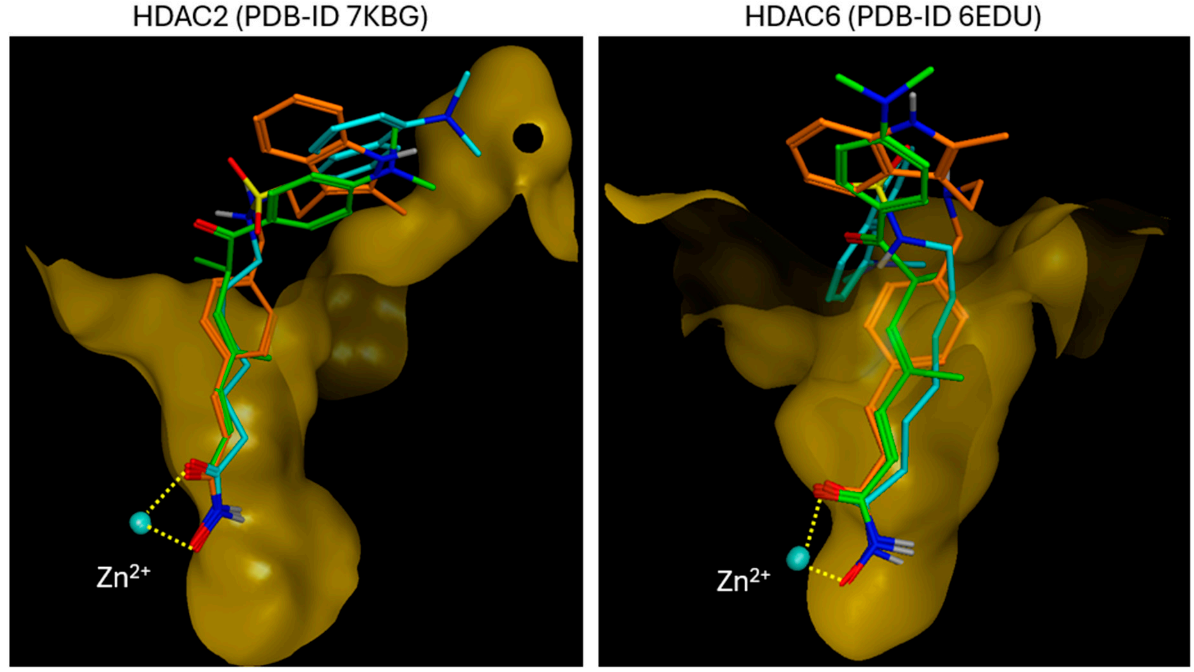

Figure S9: Predicted binding modes for trichostatin A, panobinostat, and MB-463 in the active sites of HDAC2 (PDB-ID 7KGB; left panel) and HDAC6 (PDB-ID 5DEU; right panel).

Table S7: K_M-values and substrate concentrations used in the enzyme activity assays

Enzyme	K_M (µM)	Substrate (µM)
HDAC1	78	20
HDAC2	51	20
HDAC3	36	20
HDAC4	36	20
HDAC5	33	20
HDAC6	88	20
HDAC7	61	20
HDAC8	99	50
HDAC10	55	20
HDAC11	115	80

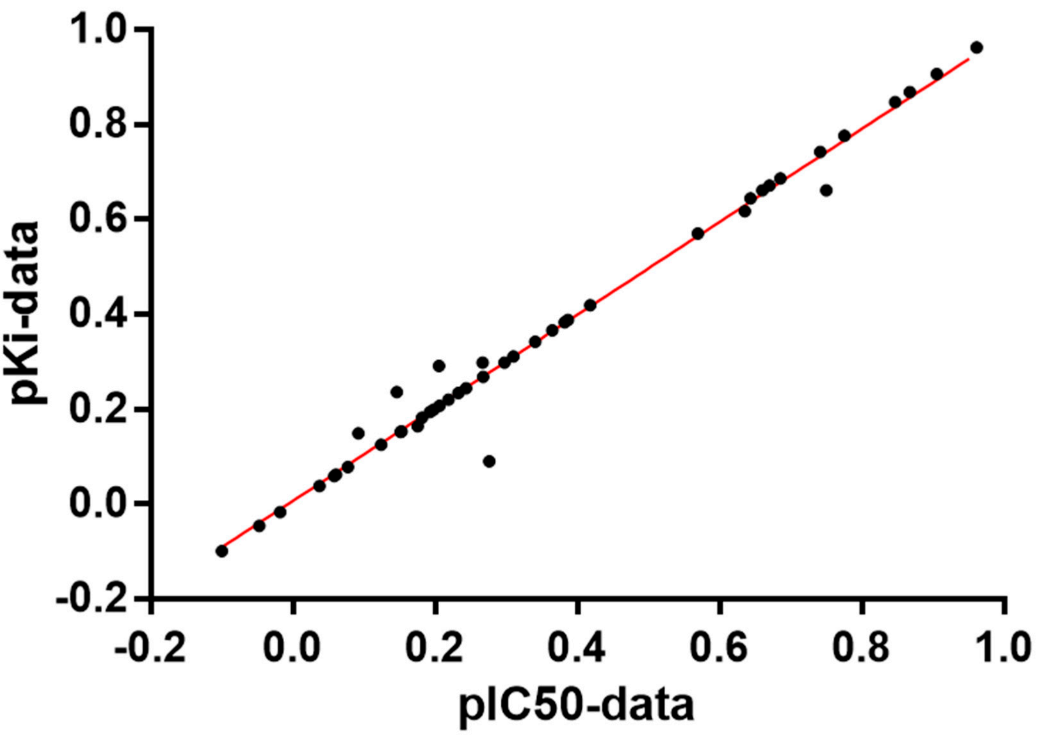

Figure S10: Korrelation of Pearson's r from pIC_{50} and pK_i (Cheng-Prusoff corrected using KM-values and substrate concentrations provided in Table S7). Each data point represents a pair of human HDACs.

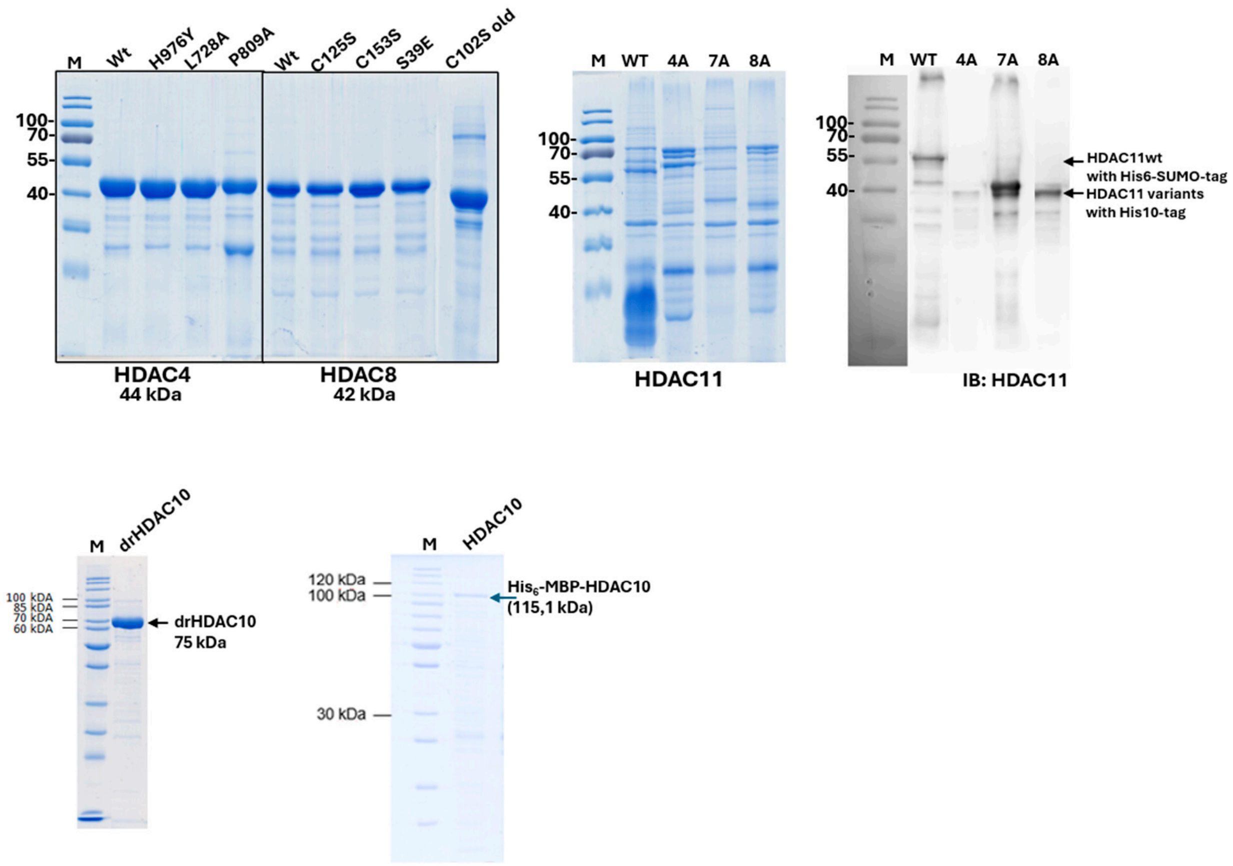

Figure S11: Protein gels and Western Blots of self-produced HDACs.
